# The synthesis and investigation of novel 3-benzoylbenzofurans and pyrazole derivatives for anti-HIV activity†

**DOI:** 10.1039/d4md00844h

**Published:** 2025-02-05

**Authors:** Sinothile S. Khuzwayo, Mamoalosi A. Selepe, Debra Meyer, Ntombenhle H. Gama

**Affiliations:** a Department of Biochemistry, Genetics and Microbiology, University of Pretoria 2 Lynnwood Road Pretoria 0002 South Africa snothilesementha81@gmail.com; b Chemistry Department, University of Pretoria 2 Lynnwood Road Pretoria 0002 South Africa; c School of Natural and Applied Sciences, Sol Plaatje University Kimberley 8300 South Africa

## Abstract

The emergence of drug-resistant viruses continues to be an obstacle to effectively controlling the HIV/AIDS pandemic. The development of novel drugs with high potency and the ability to fully eradicate HIV-1 infections is therefore of critical importance. Novel pyrazole derivatives were synthesized from 3-benzoylbenzofurans and characterized by mass spectrometry (MS) and nuclear magnetic resonance (NMR) spectroscopy. The 3-benzoyl benzofurans were determined to be highly cytotoxic in TZM-bl cells, while their pyrazole derivatives were mild to non-cytotoxic. Evaluation of anti-HIV activities in pseudoviruses revealed two 3-benzoyl benzofurans (3g and 4b) and pyrazoles (5f and 5h) as the most potent inhibitors. The IC_50_ values of 4b and 5f were 0.49 ± 0.11 μM and 0.39 ± 0.13 μM in Q23 and 0.12 ± 0.05 μM and 1.00 ± 0.15 μM in the CAP210 pseudovirus, respectively. Further evaluations for mechanism of action involved the time of addition assay and direct enzyme inhibition, which showed that 3g and 4b were non-nucleotide reverse transcriptase inhibitors while 5f and 5h inhibited HIV entry. Additionally, 3g, 4b, and 5h were found to be mild inhibitors of HIV-1 protease, while 5f was the most active protease inhibitor. The IC_50_ value of 5f was 31.59 ± 3.83 μM, and it displayed interactions with the active site of HIV-1 PR, suggesting competitive inhibition using molecular docking. The promising anti-HIV activities of 5f in pseudoviruses and HIV-PR motivate its further development for antiretroviral drugs.

## Abstract 2

The emergence of drug-resistant viruses continues to be an obstacle to effectively controlling the HIV/AIDS pandemic.

## Graphical abstract

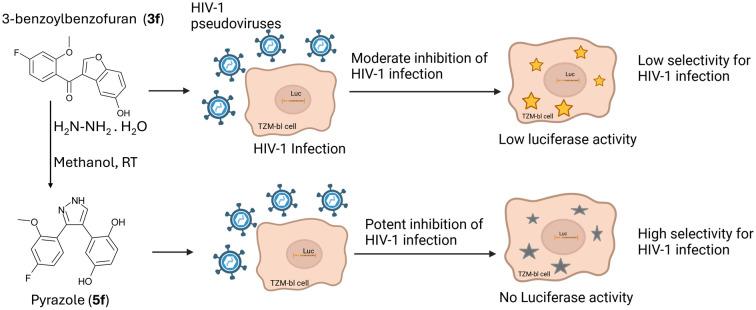

## 1. Introduction

Four decades after its discovery, infection by the human immunodeficiency virus (HIV-1) remains one of the world's leading global epidemics. UNAIDS estimated 39 million people were living with HIV in 2022, with 630 000 AIDS-related deaths reported globally in that same year.^1,2^ Eastern and southern Africa have been most severely affected by the HIV-1 epidemic, with 53.3% of HIV-infected people and 38.5% of new infections reported in 2022.^1^ South Africa reported 7.6 million people living with HIV in 2022, which makes up a total of 17.8% of adults.^3^ The number of newly infected people was 160 000, and a total of 45 000 people died from AIDS-related illnesses in South Africa alone in 2022.^3^

Rapid HIV replication and the virus' propensity for replication errors, resulting in numerous genetic variations in its offspring, provide the greatest challenges to eradicating HIV. Candidate HIV-1 vaccines have shown considerable promise, including ALVAC-HIV, a DNA vaccine made up of gp120 plasmids from five different HIV-1 subtypes;^4^ VRC01, an anti-gp120 monoclonal antibody now undergoing phase II clinical trials;^5^ and PDPHV, an envelope and gp120 vaccine.^6^ However, none of these vaccines have been approved. Therefore, the use of combination antiretroviral therapy (cART) for controlling HIV-1 infections remains a significant necessity^7^ for disease management. Approximately 78% of HIV-1-infected people in sub-Saharan Africa were receiving treatment in 2022.^8^ Unfortunately, cART also has drawbacks, including drug toxicity, which leads to treatment non-adherence, and the emergence of drug resistance.^9^

**Fig. 1:**
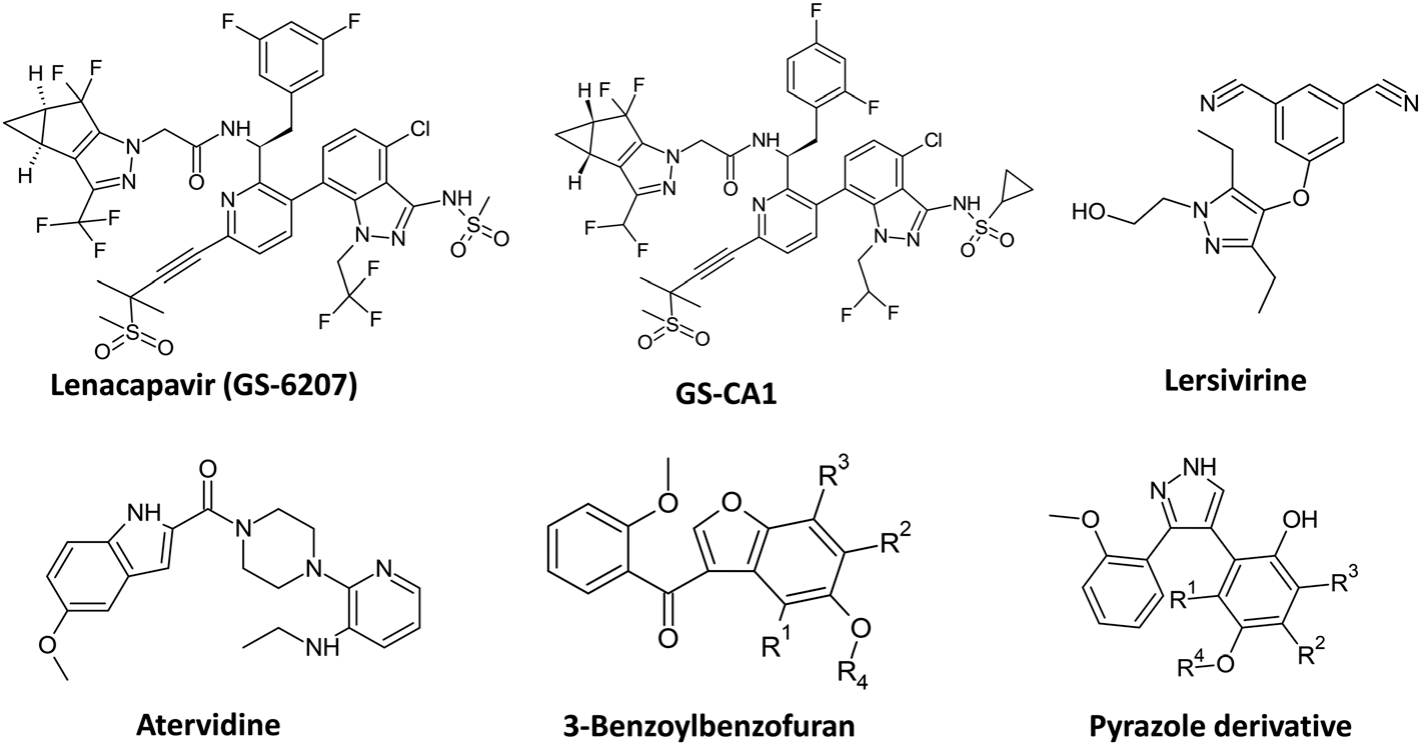
Comparison of the structures of potent HIV inhibitors with the generic structures of benzoylbenzofurans and pyrazole derivatives prepared in the current study. The pyrazole scaffold found in lenacapavir, the most potent HIV drug, was mimicked with the derivatives synthesized in this study. The benzoylbenzofurans contain acetyl and methoxyphenyl groups found in atervidine.

Recently, new drug targets for HIV have been discovered, including the inhibition of the HIV-1 capsid.^10^ The capsid protein has been identified as a promising target for HIV treatment because it not only houses the HIV genome but also aids in virus uncoating in the host cytoplasm, nuclear export, and virion assembly.^11^ Compounds GS-CA1 and its analogue GS-6207 (Fig. 1) are capsid inhibitors that have been shown to have a better potency than all the existing HIV drugs,^12^ while lenacapavir (GS-6207) was the first capsid inhibitor to be FDA-approved for HIV treatment in 2022.^13^ Additionally, lenacapavir is the first pyrazole-based drug to be approved for the treatment of HIV-1 multi-drug-resistant infections.^13^ Numerous experimental pyrazole compounds are potent inhibitors of HIV infections and HIV-1 enzymes.^14–16^ Lersivirine, a pyrazole derivative, was demonstrated to be a potent inhibitor of HIV-1 reverse transcriptase (RT). However, it was discontinued in 2013 during its phase III clinical trials due to its lack of improved efficacy over already available drugs.^17^ A pyrazole-piperidine was shown to inhibit both HIV-RT and viral entry with IC_50_ of 9 μM, 0.8 μM, and 3.8 μM for HIV-RT, CCR5, and CXCR4 co-receptors, respectively.^18^ More potent pyrazole compounds have been shown to inhibit HIV-1 RT with IC_50_ values of 0.93 and 1.28 μM.^19^ In addition to the potent anti-HIV activity, the minimal cytotoxicity of the pyrazoles makes these compounds attractive candidates for the development of antiretrovirals.^20^

Benzofurans are well-studied due to their distinguished pharmacological applications, and some of these compounds are FDA-approved drugs against various human diseases, including cancer, malaria, and various bacterial infections.^21^ Similarly to the pyrazoles, benzofuran compounds were shown to be potent inhibitors of HIV-1 RT, and their activity was higher compared to atervidine, an HIV drug that closely resembles benzofuran compounds as shown in Fig. 1.^22^ More benzofuran derivatives have been identified as HIV-1 protease (PR) inhibitors,^23,24^ These compounds include benzofuran-carboxamide derivatives, which demonstrated dual activities against both RT and PR, which further motivates the potential of the benzofuran scaffold for exploration for antiretroviral development.^25^

In the current study, the pyrazoles were synthesized from the 3-benzoyl benzofuran precursors, and both compound classes were investigated for cytotoxicity and their inhibitory effects on the HIV life cycle.

## 2. Results and discussion

### 2.1 Synthesis of 3-benzoylbenzofurans and pyrazole derivatives

**Scheme 1:**
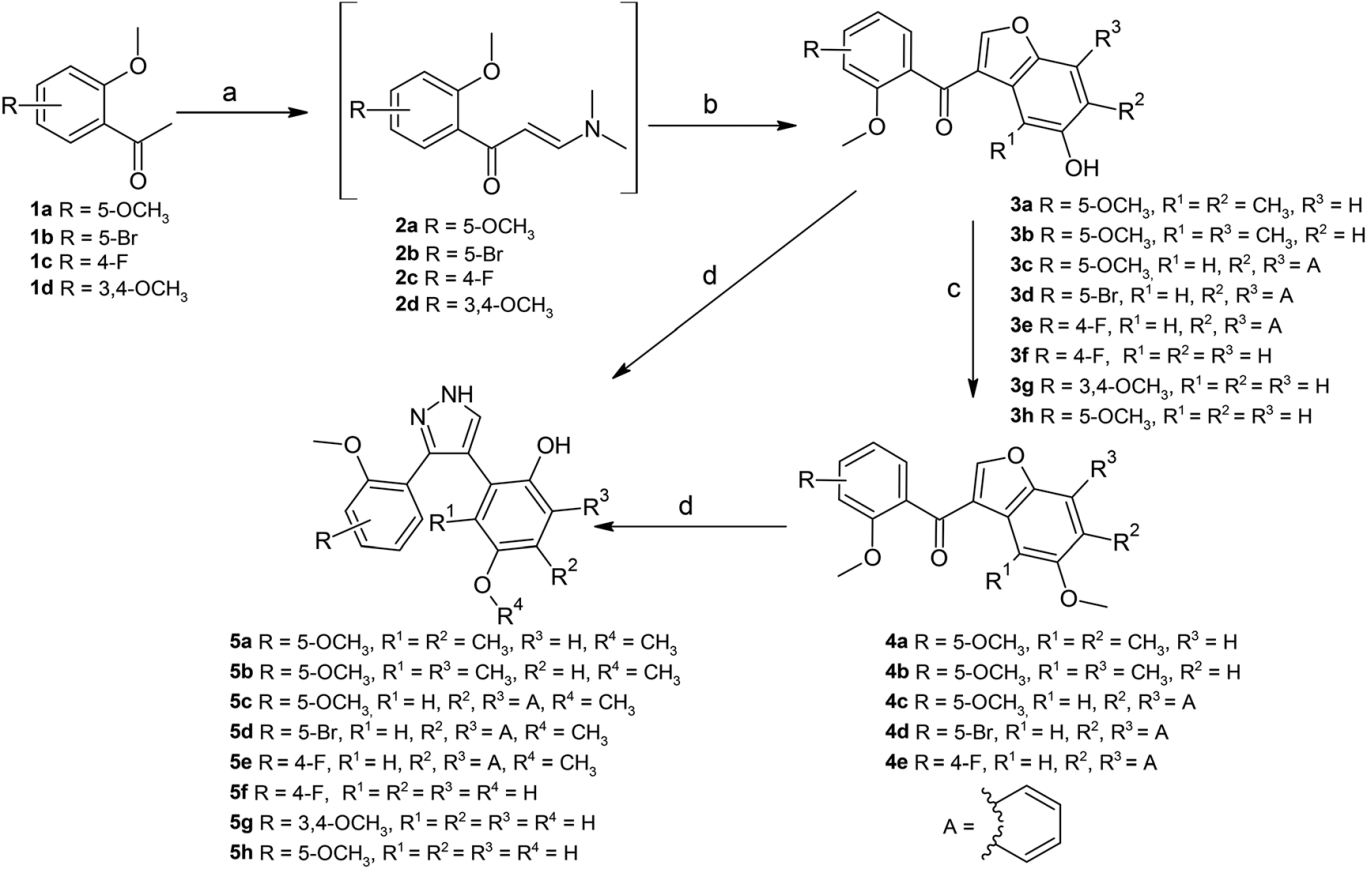
Synthesis of 3-benzoylbenzofurans and pyrazole derivatives. Reagents and conditions: (a) DMF-DMA, 90–190 °C, Ar, 12–72 h. (b) 1,4-benzoquinone/1,4-naphthoquinone, acetic acid, rt, 12 h. (c) CH_3_I, K_2_CO_3_, DMF, 100 °C, Ar, 5–12 h. (d) Hydrazine hydrate, methanol, rt, 2–5 h.

The synthesis of 3-benzoylbenzofurans 3a–h, 4a–e, and their pyrazole derivatives 5a–h is demonstrated in Scheme 1. The synthesis of 3-benzoylbenzofurans involved the condensation of 2-methoxyacetophenones 1a–d and *N*,*N*-dimethylformamide dimethyl acetal (DMF-DMA) producing enaminoketones 2a–d, which were reacted without further purification with 1,4-benzoquinone or 1,4-naphthoquinone derivatives to form the 3-benzoyl benzofurans 3a–h.^26,27^ The 3-benzoylbenzofurans were obtained in moderate to low percentage yields over two steps. Numerous methods for the synthesis of benzofurans have been reported in the literature.^28,29^ The current study produced the benzofurans using the most common method called the Nenitzescu reaction, which involves the condensation of an enaminoketone and 1,4-benzoquinone.^30^ The reaction produces two common products, the 5-hydroxyindoles and benzofuran derivatives, depending on the reaction conditions.^30,31^ The first step of the reaction is the Michael Addition of an enaminoketone to the benzoquinone, which is followed by the production of a zwitter ion, which is then O-protonated and results in a hydroquinone adduct, which under acidic conditions (acetic acid) forms a benzofuran.^30,31^ However, oxidation could also occur in less or non-acidic conditions, which results in the formation of the 5-hydroxyindole.^30,31^ The low yields of 3-benzoylbenzofurans obtained in the present study could be attributed to the production of byproducts (observed from the thin layer chromatography); however, the byproducts were not characterized.

The pyrazole derivatives were synthesized from the 3-benzoylbenzofurans using a method described by Abdel-Aziz *et al.*,^32^ which treated the 3-benzoylbenzofurans with hydrazine hydrate in the presence of methanol, forming the pyrazole derivatives. The 3-benzoylbenzofurans 3f, 3g, and 3h spontaneously reacted with hydrazine hydrate to form the pyrazole derivatives 5f, 5g, and 5h in percentage yields of 31, 69, and 16%, respectively. However, the substituted 3-benzoylbenzofurans presented difficulties when synthesizing the pyrazole derivatives. Multiple byproducts were observed when reacting these 3-benzoylbenzofurans (3a–3e) with hydrazine hydrate, and stringent purification methods were applied; unfortunately, the products were not free of impurities. The pyrazole derivatives 5a–5e were rather formed smoothly from the 5-methoxybenzoylbenzofurans 4a–4e. The compounds 4a–4e were prepared by treatment of 3-benzoylbenzofurans 3a–3e with methyl iodide (CH_3_I) in the presence of potassium carbonate (K_2_CO_3_) and dry DMF (Scheme 1). Methylation reactions have been reported to occur rapidly in the presence of K_2_CO_3_ as a base and dry DMF at high temperatures.^33^ The methylation reactions were conducted at 100 °C and the compounds were obtained in high percentage yields ranging from 71 to 96%. The methylated 3-benzoylbenzofurans (4a–4e) readily reacted with hydrazine hydrate to produce the pyrazole derivatives (5a–5e) which did not require further chromatographic purification steps. The pyrazole derivatives (5a–5e) were obtained at yields of 72–95% (Scheme 1). The obtained percentage yields and the rapidness of the reactions corresponded to other studies that obtained high yields of pyrazole compounds under similar conditions.^32,34^

The current study synthesized the pyrazole derivatives from the α,β-unsaturated ketone of 3-benzoylbenzofurans and hydrazine hydrate in methanol, which produced the 3,4-substituted pyrazole derivatives at room temperature. Numerous methods have been reported for the synthesis of pyrazoles.^35–37^ Examples include refluxing chromones and chalcones with hydrazine derivatives in ethanol.^35,36^ The 1, 3, and 5 substituted pyrazoles^36^ and the 3,5-substituted pyrazoles^35^ were obtained at high percentage yields ranging from 51–98% after recrystallization from ethanol. Contrary to other researchers,^34–36^ the current study followed a method that transformed the benzofuran moiety into the pyrazole scaffold.^32^ In this study, the yields of the pyrazole compounds depended largely on the benzofuran precursors, as it has been shown that 5-methoxybenzoylbenzofurans resulted in high yields of pyrazole products that did not require further chromatographic purification.

### 2.2 Cytotoxicity evaluations

The synthesized compounds were evaluated for cytotoxicity in TZM-bl cells using an MTT assay. Investigating the cytotoxicity of compounds in this cell line was crucial since these cells were further used for infections with pseudoviruses in the evaluation of potential anti-HIV activity. The current study demonstrated that the derivatization of cytotoxic 3-benzoylbenzofurans to pyrazoles resulted in a general decrease in cytotoxicity, as shown in Table 1. Only the pyrazole derivative 5f demonstrated increased cytotoxicity from its precursor 3f; however, this difference was not significant with a *P* value of 0.9918. There was a significant decrease in cytotoxicity of the pyrazole derivatives 5g and 5h compared to their precursors 3g and 3h respectively (*P* = 0.0005). The methylated 3-benzoylbenzofurans 4a–4e had the highest cytotoxicity, with CC_50_ values ranging from 7.77 to 59.15 μM. There was a significant decrease in the cytotoxicity upon derivatization of 4b, 4c, 4d, and 4e to pyrazoles 5b, 5c, 5d, and 5e. The decrease in the cytotoxicity for pyrazole derivatives 5b, 5c, 5d, and 5e ranged from 3 to 10-fold. The cytotoxicity of 5a was only 1-fold lower than that of the precursor 4a; this was not significantly different. Overall, these results show that converting the benzofuran scaffold to a pyrazole decreases the cytotoxicity of the compounds which is an excellent characteristic of potential HIV treatment.

**Table 1:** Evaluation of cytotoxicity of compounds in TZM-bl cells by MTT assay

Benzoylbenzofurans	CC_50_ (μM) ± SEM (TZM-bl cells)	Pyrazole derivatives	CC_50_ (μM) ± SEM (TZM-bl cells)
4a	59.15 ± 6.56	5a	88.85 ± 22.10
4b	8.42 ± 1.16	5b	46.45 ± 6.66*
4c	9.65 ± 2.37	5c	47.69 ± 17.65*
4d	7.77 ± 1.54	5d	74.96 ± 6.40
4e	35.19 ± 2.32	5e	103.40 ± 11.14*
3f	41.94 ± 6.33	5f	36.07 ± 7.95
3g	79.85 ± 7.25	5g	140.10 ± 17.13*
3h	49.98 ± 10.47	5h	142.30 ± 13.24*
Control (cisplatin)	56.67 ± 5.20		

Pyrazole compounds are known for their minimal cytotoxicity in human cells,^19,20^ while benzofurans are well known for their cytotoxicity and are more common in cancer treatment.^38,39^ The compound cytotoxicity data obtained in this study agreed with available literature about benzofuran and pyrazole cytotoxicity, except for the fluorinated pyrazole derivative (5f) which was more cytotoxic than its benzofuran precursor. However, fluorine has been well-documented to increase the toxicity of benzofuran compounds.^40^ Similar to benzofurans, the addition of single or multiple fluorine atoms to the pyrazole derivatives has been shown to increase the lipophilicity of the drugs, thus allowing for better absorption, but unfortunately also increasing cytotoxicity.^41^ The current study observed that methylating 3-benzoylbenzofurans increased their lipophilicity (Mlog *P*) by a value greater than 0.2 (Table 6). This was expected, given that the methoxy functional group has previously been demonstrated to contribute to lipophilicity more than the hydroxy group.^42^

The mechanisms of apoptosis for chemotherapy drugs, including cisplatin, involve the induction of reactive oxygen species,^43^ DNA intercalation,^44^ and increased expression of caspases 3/7 and p53, which are tumour suppressor proteins.^45^ Benzofurans^46,47^ and pyrazoles^48,49^ have been demonstrated to cause cytotoxicity through similar mechanisms. The low cytotoxicity of pyrazole derivatives has been reported by many researchers^16^ and this may be attributed to the physicochemical properties of the pyrazole scaffold. Researchers demonstrated that drugs with topological polar surface area (TPSA) values lower than 75 Å have significantly higher toxicity than drugs with larger TPSA values.^50^ Similar phenomena applied to the 3-benzoylbenzofurans, and their pyrazole derivatives were investigated in this study. The 3-benzoylbenzofurans had lower TPSA values, with the most toxic compound 4b having a TPSA of 57.90 Å and its pyrazole derivative 5b having a TPSA value of 76.60 Å (Table S1†). All the pyrazole derivatives had TPSA values that were higher by 18.70 Å compared to their precursors, showing the modification caused by the pyrazole scaffold. Therefore, the remarkably low cytotoxicity observed with the pyrazole derivatives prepared in this study was due to their physicochemical properties. Additionally, the mechanisms of apoptosis were not explored in this study. Therefore, it is inconclusive that mechanism of apoptosis for 3-benzoylbenzofurans may be altered because of derivatization to pyrazoles.

### 2.3 Inhibition of HIV-1 infections by 3-benzoylbenzofurans and pyrazole derivatives

**Fig. 2:**
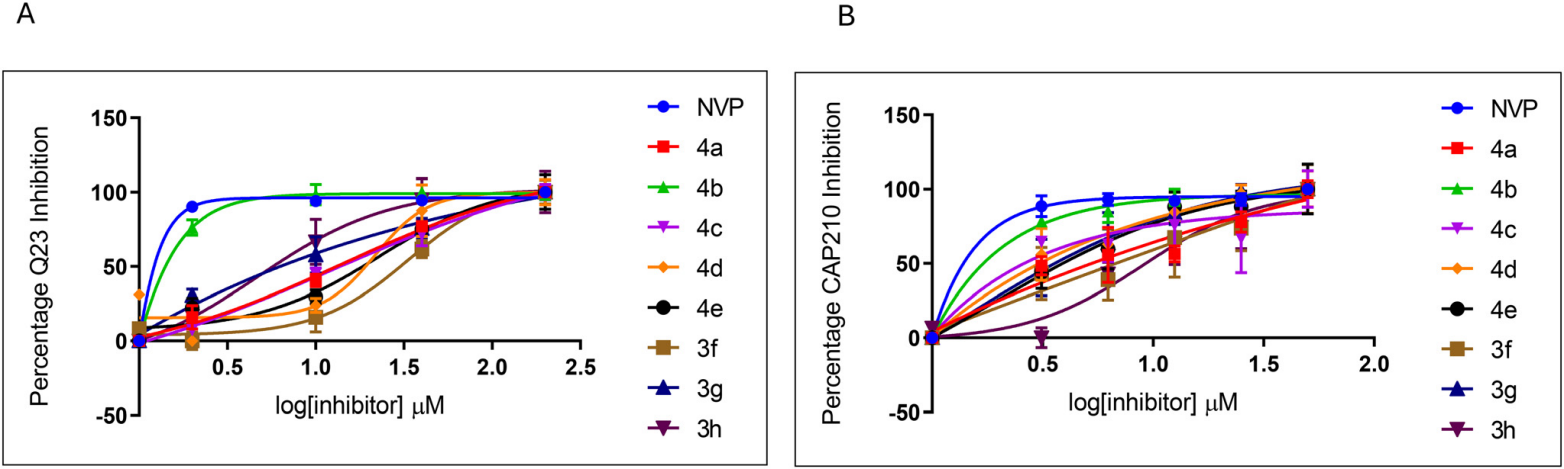
Inhibition of HIV-1 infections by the 3-benzoylbenzofurans. The 3-benzoylbenzofurans were evaluated against Q23 (A) and CAP210 (B). Nevirapine (NVP) was used as a control at similar concentrations, and 99% inhibition was obtained in all tested concentrations. The bars represent the mean ± SEM obtained from three independent experiments.

The 3-benzoylbenzofurans were investigated for the inhibition of HIV-1 Q23 and CAP210 pseudoviruses, and as can be seen in Fig. 2, both Q23 and the CAP210 pseudoviruses were inhibited by the 3-benzoylbenzofurans. The IC_50_ values of the 3-benzoylbenzofurans in the Q23 pseudovirus ranged from 0.49 to 40.58 μM, as shown in Table 2. The 3-benzoylbenzofurans demonstrated higher inhibitory activity to the CAP210 pseudovirus with the IC_50_ values ranging from 0.12 to 11.16 μM (Table 2). 4b, which is a (2,5-dimethoxybenzoyl)-4,7-dimethylbenzofuran, was the most potent inhibitor with IC_50_ values of 0.49 ± 0.11 μM and 0.12 ± 0.05 μM in Q23 and CAP210 viruses, respectively. 3g, a (2,3,4-trimethoxybenzoyl)benzofuran, was the second active 3-benzoylbenzofuran in the Q23 pseudovirus with an IC_50_ of 3.90 ± 1.63 μM. Compound 3f, which is the 4-fluoro-2-methoxybenzoylbenzofuran, was the least active with IC_50_ values of 40.58 ± 7.85 μM in Q23 and 11.16 ± 0.31 μM in the CAP210 pseudovirus.

**Table 2:** Inhibition of HIV-1 Q23 and CAP210 pseudoviruses by the 3-benzoylbenzofurans and their corresponding selectivity indices (SI)

Compounds	IC_50_ (μM) Q23	SI (Q23)	IC_50_ (μM) CAP210	SI (CAP210)
4a	18.12 ± 1.78	3.26	8.12 ± 2.56	7.26
4b	0.49 ± 0.11	17.18	0.12 ± 0.05	70.17
4c	12.56 ± 0.53	0.76	2.43 ± 1.28	3.97
4d	22.44 ± 3.47	0.35	3.28 ± 1.48	2.36
4e	20.95 ± 2.20	1.68	4.27 ± 0.19	8.24
3f	40.58 ± 7.85	1.03	11.16 ± 0.31	3.76
3g	3.90 ± 1.63	20.47	4.91 ± 1.18	16.26
3h	5.74 ± 1.51	8.71	4.45 ± 0.82	11.23
NVP	0.11 ± 0.004	>500	0.10 ± 0.002	>500

Selectivity indices (SI) represent the ratio of the CC_50_ of drugs against healthy cells to the IC_50_ of compounds against pathogens, and an SI value higher than 1 suggests favourable inhibition.^51^ In Q23, the 3-benzoylbenzofurans 4c, 4d, and 3f displayed low SI values ranging from 0.35 to 1.03, indicating general toxicity and non-specificity. However, in CAP210 pseudoviruses, these 3-benzoylbenzofurans had higher SI values ranging from 2.36 to 3.97 (Table 2). The 3-benzoylbenzofurans 4a, 4b, 3h, and 3g had high SI values that ranged from 3.26 to 20.47 and 7.26 to 70.17 in Q23 and CAP210 pseudoviruses, respectively (Table 2). Therefore, only compounds 4a, 4b, 3g, and 3h were considered to be inhibitors of the Q23 pseudovirus, while all the 3-benzoylbenzofurans inhibited the CAP210 pseudovirus, with SI values of above 1 for all the compounds. The CAP210 pseudovirus belongs to the HIV-1 subtype C strain, which accounts for 46.6% of infections globally and 98% of HIV infections in South Africa.^52^ Research has shown that the subtype C HIV viruses maintain intact proviruses, which gives them an advantage for better adaptation in a new host and, therefore, increased transmission.^53^ This study reported 3-benzoylbenzofurans with potent inhibitory activities against HIV subtype C, the most prevalent HI-virus in South Africa. Additionally, while the 3-benzoylbenzofurans are active against subtype C HIV infections, they represent medication that potentially won't be effective against the other eight HIV subtypes of group M, which are prevalent throughout Europe, Asia, East and West Africa, and the Caribbean. In contrast to subtype C, these HIV subtypes are less virulent.^54^ Among the 3-benzoylbenzofurans, 4b was the most effective inhibitor found; however, it was still far less potent than the common non-nucleotide reverse transcriptase inhibitors (NNRTIs) zidovudine (DZV) and nevirapine (NVP), which have been demonstrated to inhibit HIV-1 subtype C pseudoviruses with IC_50_ values of 0.05 uM and 0.03 uM, respectively.^55^

**Fig. 3:**
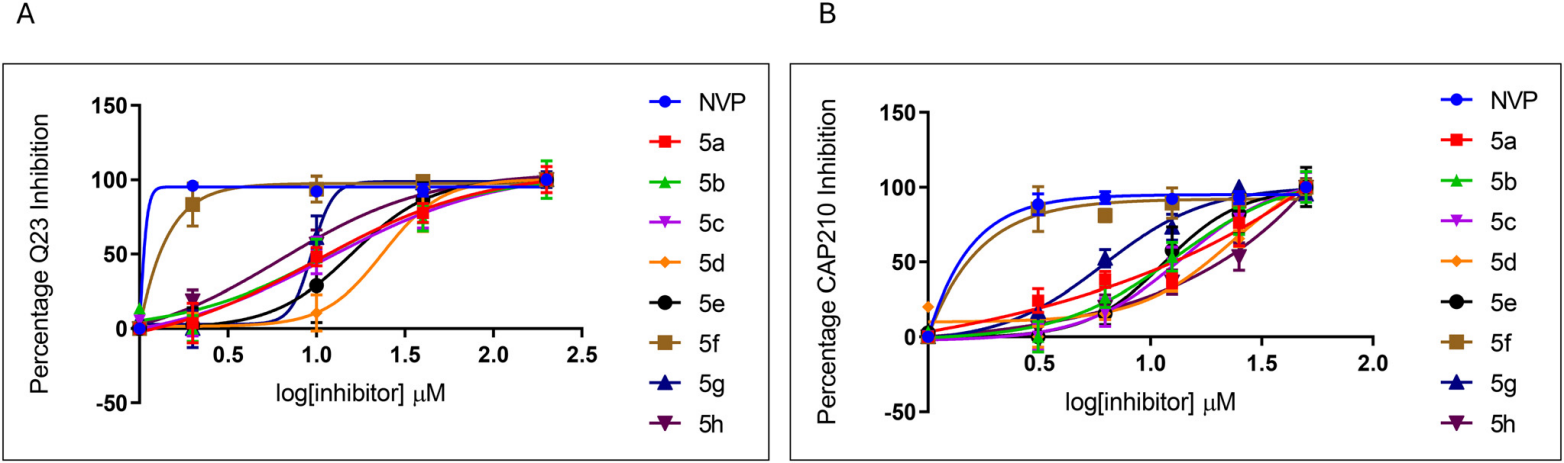
Inhibition of HIV-1 infections by the pyrazole derivatives. The pyrazoles were evaluated against Q23 (A) and CAP210 (B). NVP was used as a control at similar concentrations, and 99% inhibition was obtained in all tested concentrations. The bars represent the mean ± SEM obtained from three independent experiments.

Pyrazole derivatives of the 3-benzylbenzofurans were successfully synthesized and these were also evaluated against Q23 and CAP210 pseudoviruses, as shown in Fig. 3. The pyrazole derivatives inhibited HIV infections by both pseudoviruses. Compound 5f was the most potent pyrazole derivative, with IC_50_ values of 0.39 ± 0.13 μM in Q23 and 1.00 ± 0.15 μM in CAP210 pseudovirus, as shown in Table 3. The pyrazoles 5g and 5h also demonstrated good activities with both compounds having IC_50_ values lower than 10 μM in Q23 and 13.80 ± 1.70 μM and 6.31 ± 0.31 μM in CAP210, respectively. As shown in Fig. 3, the pyrazole derivative 5f exhibited over 90% anti-HIV activity at the lowest concentrations tested, similar to NVP. However, the current study did not test lower concentrations of NVP to determine its actual IC_50_. Therefore, it can be assumed that the IC_50_ for NVP is much lower than 0.11 μM. Literature reports typically show IC_50_ values around 0.03 μM or lower against various HIV-1 subtypes.^55^ Thus, NVP and other HIV drugs are significantly more potent (in the nano and picomolar range) than 5f. Nonetheless, compound 5f remains the most potent inhibitor among the pyrazoles investigated in this study. Due to the pyrazole scaffold in 5f, which has been shown to reduce toxicity, 5f is the most promising compound identified in this study for further development as an antiretroviral agent.

**Table 3:** Inhibition of HIV-1 Q23 and CAP210 pseudoviruses by pyrazole derivatives and their corresponding selectivity indices (SI)

Compounds	IC_50_ (μM) Q23	SI (Q23)	IC_50_ (μM) CAP210	SI (CAP210)
5a	13.32 ± 1.12	6.67	18.38 ± 3.20	4.83
5b	10.09 ± 1.66	4.60	7.87 ± 0.91	5.90
5c	12.96 ± 3.75	3.68	20.49 ± 6.80	2.33
5d	16.21 ± 2.55	4.62	20.77 ± 1.00	3.61
5e	15.68 ± 8.17	6.60	29.09 ± 0.18	3.55
5f	0.39 ± 0.13	92.49	1.00 ± 0.15	36.43
5g	9.93 ± 0.69	14.11	13.80 ± 1.70	10.15
5h	8.14 ± 2.63	17.48	6.31 ± 0.31	22.55
NVP	0.11 ± 0.04	>500	0.10 ± 0.02	>500

Pyrazoles 5f, 5g, and 5h have hydroquinone substituents on carbon 4 but differ in the carbon 3 substituents which are 4-fluoro-2-methoxyphenyl, 2,3,4-trimethoxyphenyl and 2,5-dimethyphenyl respectively. Amongst these compounds, 5f, which is a fluorinated pyrazole, had the highest anti-HIV activities (Fig. 3). 5g and 5h had activities that were significantly lower than NVP. Fluorinated pyrazoles have been well documented for their high potency against HIV;^4,56,57^ therefore, 5f was expected to demonstrate the highest activity. Based on these findings, it can be deduced that the fluorine of 5f influenced the anti-HIV activities more than the hydroquinone substituent, which is also found in 5g and 5h, which had lower anti-HIV activities. Additionally, these derivatives (5f, 5g, and 5h) demonstrated high selectivity indices, which ranged from 3.68 to 92.49 in Q23 and 2.33 to 36.43 in the CAP210 pseudovirus, making them all favourable inhibitors of HIV-1 infections (Table 3). The difference in IC_50_ values of pyrazole derivatives in Q23 and CAP210 viruses was not significant; therefore, these compounds could potentially be developed for the treatment of both subtype A and C infections. Subtypes A and C have been documented to have approximately 15 to 20% differences in their genetic sequences; therefore, it is plausible that similar drugs would inhibit both viruses.^58^

**Fig. 4:**
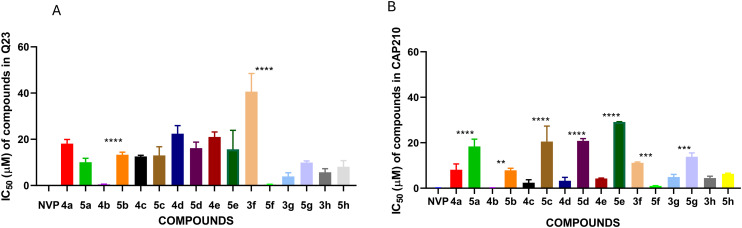
Comparison of inhibition of Q23 (A) and CAP210 (B) infections by 3-benzoylbenzofurans and pyrazole derivatives. The superscripts *, **, ***, and **** represent the compound pair with significantly higher activity than their precursors with *P* ≤ 0.05, *P* ≤ 0.01, *P* ≤ 0.001, and *P* ≤ 0.0001, respectively. 4b and 5f had significantly higher activity than their derivative and precursor in Q23, respectively. Only 5f had a significantly higher activity in CAP210, while all benzofurans except 3h were significantly more active than their pyrazole derivatives in CAP210. The bars represent the mean ± SEM obtained from three independent experiments.

The anti-HIV activities of 3-benzoylbenzofurans and their pyrazole derivatives were compared in Q23 and CAP210, as shown in Fig. 4. There was no significant difference between the anti-HIV activity of 3-benzoylbenzofurans (4a, 4c, 4d, 4e, 3g, and 3h) and their pyrazole derivatives (5a, 5c, 5d, 5e, 5g, and 5h) in Q23 (Fig. 4A), while the anti-HIV activity of 3-benzoylbenzofuran 4b was significantly higher than that of its pyrazole derivative 5b. The pyrazole derivative 5f also had a significantly higher activity than its precursor 3f. There was no significant difference between the IC_50_ values of NVP, 4b, and 5f, which were 0.11 ± 0.04, 0.49 ± 0.11, and 0.39 ± 0.13 μM, respectively. Even though derivatization to pyrazoles did not significantly increase the anti-HIV activities of the majority of the compounds, there were noticeable differences in specificity for HIV strains. The 3-benzoylbenzofurans were more specific for CAP210, while the pyrazoles had general activity against both subtype A and C viruses (Fig. 4). Therefore, cART formulated from these pyrazole derivatives would be equally effective against various HIV strains, while 3-benzoylbenzofurans would produce cART that could only be effective in certain strains, thus impending efforts to eradicate the HIV epidemic.

**Fig. 5:**
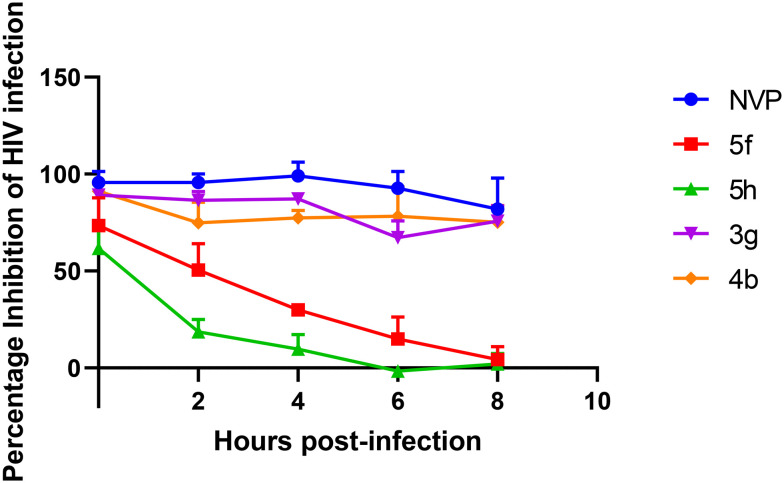
Time of addition assay of CAP210 HIV infection treated with active compounds. TZM-bl cells were infected with CAP210 pseudoviruses and treated with compounds at various hours post-infection. NVP, 3g, and 4b were used at 3 μM, while 5f and 5h were used at 25 μM. The bars represent the mean ± SEM obtained from three independent experiments.

A time of addition assay was conducted in an attempt to elucidate the target of the most potent 3-benzoylbenzofurans (3g and 4b) and pyrazoles (5f and 5h). The compounds were added from the onset of infections and every two hours for 8 hours. 3g and 4b were observed to inhibit HIV infections for up to 8 hours similarly to NVP, a reverse transcriptase inhibitor (Fig. 5). 5f and 5h only inhibited HIV infections when added on the onset of infection, then the inhibition significantly decreased from 2 hours post-infection. These findings suggest that the pyrazoles target the entry stage of infections, while the 3-benzoylbenzofurans potentially target reverse transcription, which occurs 5 to 8 hours post-infection.^59^ Since the structure of the 3-benzoylbenzofurans does not closely resemble the DNA nucleotides, which are substrates for HIV RT, it can be deduced that these compounds are NNRTIs like NVP.

Pyrazoles 5f and 5h were demonstrated to exhibit inhibition against the early stages of infection; therefore, they could either target the viral receptors gp120 and CD4, host cell receptors like CD4 and CCR5, or the fusion, which all occur within the first two hours of HIV infection.^59^ Previous studies have identified pyrazole derivatives that inhibit HIV entry through binding to CCR5 and CXCR4, which are potential targets for 5f and 5h.^18^ Pyrazole 5f was six times more active than this pyrazole-piperidine compound at inhibiting HIV infections. Moreover, the pyrazole-piperidine compounds had dual targets, including HIV entry and HIV RT, which was not observed with 5f. 5f is a fluorophenylpyrazole, and fluorinated pyrazoles are more potent inhibitors of HIV-1 infection.^57^ Evidence of this is the recently approved long-acting capsid inhibitor lenacapavir, which inhibits more than 23 HIV-1 isolates with an IC_50_ ranging from 20 to 160 pM,^12^ making it significantly more potent compared to 5f, which has an IC_50_ ranging from 0.39 to 1.00 μM. Furthermore, 5f has demonstrated no selectivity for HIV subtypes A and C, suggesting that it may inhibit a variety of HIV isolates. Therefore, 5f holds potential for development as an anti-HIV drug. Future studies could focus on performing structural modifications to enhance the anti-HIV activity of compound 5f. Modifications to 5f, such as incorporating active functional groups like trifluoromethyl (CF_3_) and sulfonyl (SO_2_) groups, may prove beneficial. These groups are known to enhance lipophilicity, thereby improving membrane absorption; increase stability, leading to a longer half-life and potential for long-acting formulations; and enhance binding affinity to viral targets. Furthermore, these functional groups are present in several HIV drugs, including protease inhibitors (darunavir, atazanavir), integrase inhibitors (raltegravir, dolutegravir), and capsid inhibitors (lenacapavir), and their inclusion in 5f could enhance its anti-HIV activity.^60–62^ However, these modifications would need to be validated in lead optimization studies, which would include target-based validation.

### 2.4 Inhibition of HIV-1 protease (PR) inhibition

**Fig. 6:**
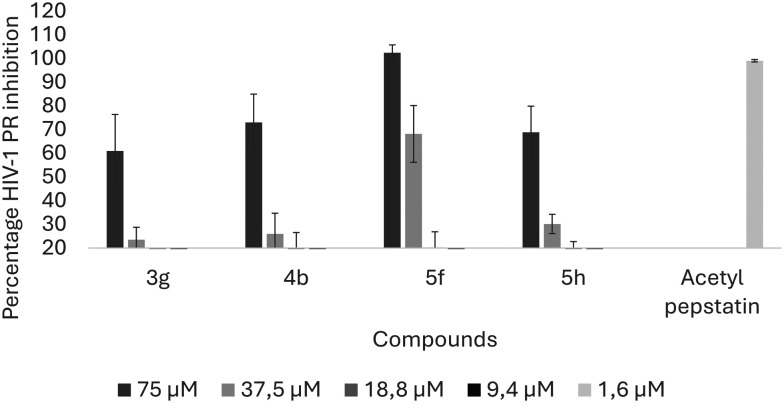
Inhibition of HIV-1 PR by active compounds. All the compounds resulted in percentage inhibition above 50% at the highest concentration with 5f demonstrating the highest activity at most of the tested concentrations. Acetyl pepstatin (AP) was used at 1.6 μM and afforded 99.99% PR inhibition. The four compounds were screened at four decreasing concentrations. The bars represent the mean ± SEM obtained from three independent experiments.

Previous studies reported 3-benzoylbenzofurans with dual inhibitory activities against HIV RT and PR.^25^ The current study screened the most potent 3-benzoylbenzofurans and pyrazoles in a direct enzyme inhibition assay against HIV PR. Dose-dependent inhibition of the enzyme was observed against PR (Fig. 6). All the compounds demonstrated more than 50% inhibition of HIV-1 PR at the highest tested concentration.

The pyrazole derivatives were the most potent inhibitors of PR, with 5f having the highest IC_50_ value of 31.59 μM and 5h having an IC_50_ value of 50.68 μM (Table 4). The cytotoxicity of pyrazoles was evaluated in HIV-negative peripheral blood mononuclear cells (PBMCs) and demonstrated low to mild cytotoxicity as demonstrated in Table 4. The selectivity indices of 5f and 5h were 3.1 and 1.6 respectively, demonstrating that they are true inhibitors of HIV-1 PR.

**Table 4:** Inhibitory activities of active compounds against HIV-1 PR, toxicity in PBMCs and their corresponding selectivity indices

Compound	IC_50_ (μM) HIV-1 PR	CC_50_ (μM) PBMCs	SI (HIV-1 PR)
3g	70.90 ± 12.41	89.54 ± 11.60	1.3
4b	57.48 ± 10.13	41.29 ± 6.78	0.7
5f	31.59 ± 3.83	98.21 ± 1.30	3.1
5h	50.68 ± 0.78	83.22 ± 9.70	1.6
AP	>1.6	—	—

Among the 3-benzoylbenzofurans, only 3g inhibited HIV PR activity with an IC_50_ of 70.90 μM, while 4b did not inhibit HIV-1 PR due to its low selectivity index, which demonstrated its lack of selectivity for the virus against healthy human cells (Table 4). These results demonstrate that the pyrazole derivatives inhibit HIV PR activity, while the 3-benzoylbenzofurans show mild to no inhibition of the enzyme.

The compounds were docked against HIV-1 PR to evaluate whether the observed inhibition was attributed to the competitive interactions of the compounds with the active site of the enzyme. Docking of compounds allows for the identification of the potential mechanism of action as well as identifying biological targets. Adeniyi *et al.* (2013) reported that docking scores lower than −5 kcal mol^−1^ represent the highest binding energies, making them acceptable docking scores for potential inhibitors.^63^ HIV-1 PR is a homodimer, and between these two subunits is a hydrophobic core and two Asp25 residues, which make up the active site of the enzyme (Fig. S2†). For the inhibition to take place, the drug binds to the active site, and the flaps enclose, trapping the inhibitor inside the active site interface, which consequently prevents further incoming and exiting of substrates.^64^ HIV-1 PR active site residues include Arg8, Asp25, Gly27, Asp29, Asp30, Gly48, Ile50, and Thr80, and protease Inhibitors (PIs) have been demonstrated to interact with these residues.^65^ The compounds were docked into the active site, with saquinavir (SQV), a potent PI, and acetyl pepstatin (AP) as docking controls to confirm the docking method. Docking scores of −10.06 and −9.42 were obtained for SQV and AP, respectively; and as expected, these ligands were shown to interact with active site residues, including Asp25, from both subunits, which are critical for enzyme activity (Fig. 6).

Only the pyrazole derivatives (5f and 5h) demonstrated good docking scores of −5.62 and −6.18 kcal mol^−1^ (Table 5). Compounds 5f and 5h interacted with two active site residues (Asp30 and Ile50) (Fig. 6). However, unlike SQV, AP and the 3-benzoylbenzofurans, the pyrazoles did not interact with Asp25 in the enzyme's active site. Importantly, like all the competitive inhibitors of HIV-1 PR, both pyrazoles interacted with Ile50, which is the flap residue, and at least one active site residue, which suggests these compounds would still occupy the active site, enclose the flaps and prevent the incoming protease substrate.^66^ Han *et al.* (1998) reported a pyrazole derivative that interacted with Asp30 through the NH group of the pyrazole scaffold.^67^ The pyrazoles in the current study interacted with Asp30 through the hydroxyl group of the hydroquinone substituents (Fig. 6). Additional interaction of another OH group from the hydroquinone with Ile50 residues and H_2_O in the active site was also observed (Fig. 6).

**Table 5:** Docking of compounds against the HIV-1 protease (1HIV) active site

Compounds	Docking scores (kcal mol^−1^)
3g	−4.87 (Asp25 (A), Asp25 (B), Ile50)
4b	−4.35 (Asp25 (A), Ile50)
5f	−5.62 (Asp30 (B), Ile50)
5h	−6.18 (Asp30 (B), Ile50)
Saquinavir	−10.06 (Asp25 (A), Asp25 (B), Gly27 (A), Arg8 (A), Ile50)
AP	−9.42 (Asp25 (A), Asp25 (B), Gly27(A), Gly48 (B), Ile50)

**Fig. 7:**
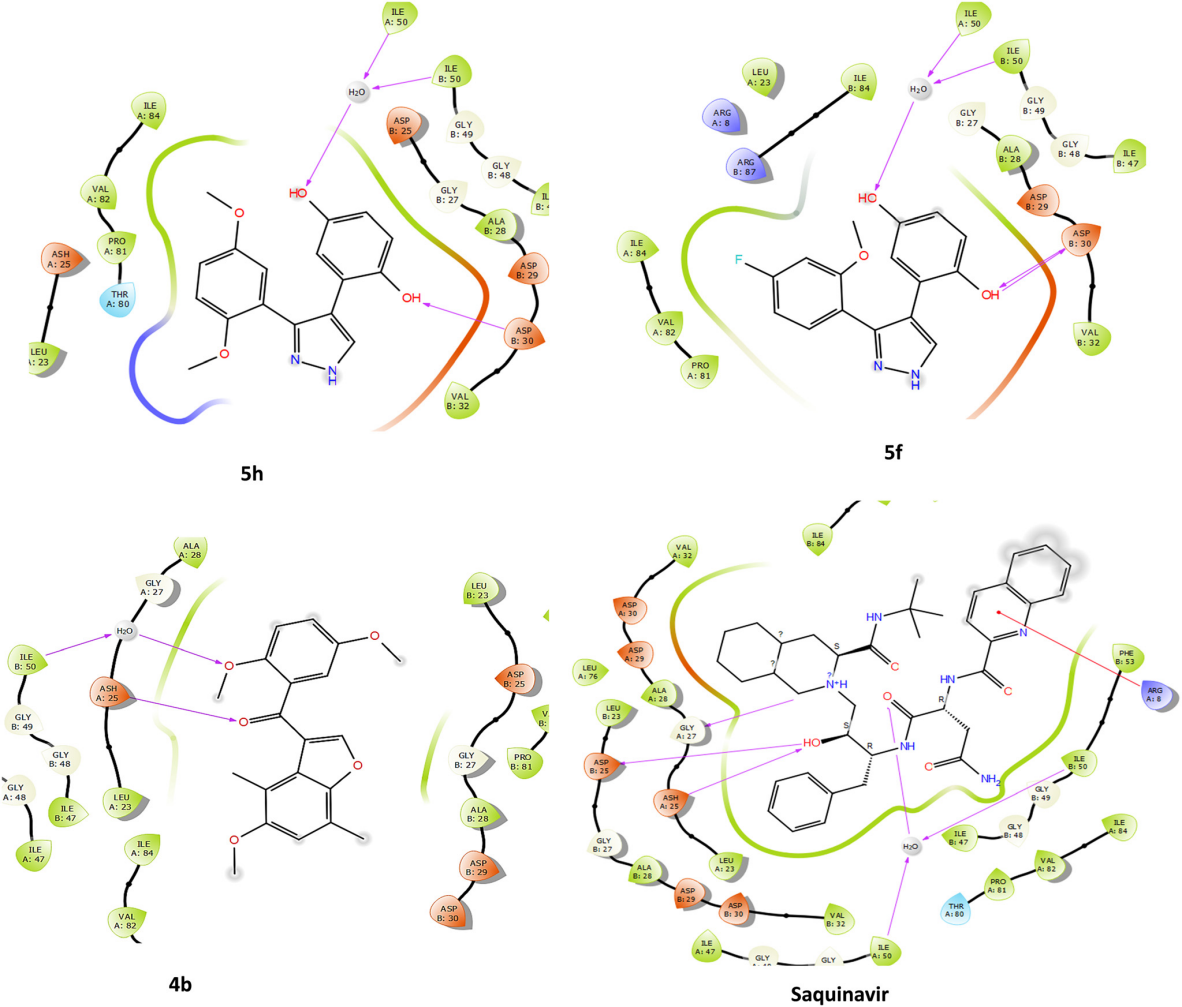
Comparison of the interactions of active compounds and controls with the active site of HIV-1 PR crystal structure (PDB: 1HIV). The figure was generated using Maestro 13.1 ligand interaction.

The 3-benzoylbenzofurans showed poor docking scores (−4.35 and −4.87 kcal mol^−1^ for 4b and 3g), indicating weak interactions with the active site (Table 5). Surprisingly, the 3-benzoylbenzofurans interacted with Asp25 through the carbonyl oxygen, and the OCH_3_ of the benzoyl group interacted with H_2_O and Ile50 of the enzyme (Fig. 7). Therefore, the 3-benzoylbenzofurans competitively inhibit HIV-1 PR, even though they are mild inhibitors due to their smaller size, which limits their interactions with other active site residues.

The 3-benzoylbenzofurans and pyrazole derivatives investigated in this study exhibited mild HIV protease inhibition, with acceptable selectivity, as shown in Table 5. These compounds could be further modified to enhance their inhibition of HIV protease, thereby improving both their potency and selectivity. Potential modifications could include the incorporation of functional groups that form key interactions with the protease active site. Such groups include hydroxy, amine, and amide, which can form hydrogen bonds with the Asp25 and Asp30 residues. Additionally, crucial π–π interactions between the aromatic groups and the flap residues (Ile50 of both subunits) could further strengthen binding. These modifications would enhance the compounds' binding affinity for the HIV protease active site, reducing off-target interactions and minimizing potential toxicity.

### 2.5 Analysis of drug-likeness of active compounds

Numerous drug compounds have been observed to fail during drug development due to poor absorbed, distributed, metabolized, excreted, and toxic (ADMET) profiles. Excellent oral bioavailability is crucial for the drug to proceed with clinical trials. Lipinski's rule of five describes the lead compounds' physicochemical properties, which determine their bioavailability potential.^68,69^ These physicochemical properties include a molecular weight of less than 500 g mol^−1^, the number of H-bond donors (OH and NH) of less than 5, the number of H-bond acceptors (O and N atoms) of less than 10, and a partition coefficient (log *P*) of less than 5.^68^ Additionally, the Pfizer 3/75 rule predicts the toxicity of drug compounds with constraints of log *P* greater than 3 and TPSA lower than 75 Å^2^.^70^

All the 3-benzoylbenzofurans and pyrazole derivatives analyzed in this study were within the parameters of Lipinski's rule of five, as shown in Table 6. Surprisingly, lenacapavir and SQV did not obey Lipinski's rule, although the drugs are FDA-approved for HIV treatment. Additionally, adherence to the Pfizer rule was investigated, 3-benzoylbenzofuran (4b) was the only compound that did not obey the Pfizer rule, which was attributed to its low TPSA of 57.90 Å^2^. This also proves the validity of the *in vitro* cytotoxicity results, which demonstrated 4b as the most toxic compound (Table 1). Compound 3g and the pyrazole derivatives obeyed the Pfizer rule and were demonstrated to be mild to non-cytotoxic *in vitro*. Lenacapavir and saquinavir agreed with the Pfizer rule, which was attributed to their large TPSA, which makes these drugs non-cytotoxic. The lack of cytotoxicity is important for novel HIV drugs since toxicity poses a threat to adherence to treatment and, consequently, the emergence of drug resistance.^71^ The pyrazole derivatives gained popularity in HIV research due to their low cytotoxicity in human cells.^16^ This study has confirmed both *in vitro* and *in silico* that derivatizing benzofurans to pyrazoles indeed decreases the toxicity of compounds, thus making them better HIV drug candidates. No PAINS alerts were predicted with both the 3-benzoylbenzofurans and pyrazoles investigated in this study, which suggests the compounds are not promiscuous and are suitable to be in a drug discovery pipeline.^72^

**Table 6:** Analysis of drug-likeness of 3-benzoylbenzofurans and pyrazole derivatives according to the Lipinski and Pfizer rule

Compound	MW (g mol^−1^)	MLog *P*	nHA	nHD	TPSA Å^2^	Lipinski rule	Pfizer rule	PAINS alerts
3g	328.32	0.74	6	1	78.13	Yes	Yes	0
4b	340.37	1.75	5	0	57.90	Yes	No	0
5f	300.28	1.78	5	3	78.37	Yes	Yes	0
5h	342.35	0.79	6	3	96.83	Yes	Yes	0
Saquinavir	670.84	1.40	7	5	166.75	No	Yes	0
Lenacapavir	968.30	4.86	12	2	174.70	No	Yes	0

This study synthesized novel 3-benzoylbenzofurans and their pyrazole derivatives that inhibited HIV-1 pseudoviruses and HIV-1 proteases and exhibited good physiochemical properties, including low cytotoxicity. These novel compounds, therefore, have the potential to be further investigated for development into antiretroviral treatments. Further studies on the active compounds could include investigation for additional targets such as screening for their potential as HIV entry inhibitors through a time-of-addition study and inhibition of HIV capsid, especially pyrazole derivatives since their scaffold is similar to that of lenacapavir. Since the pseudovirus experiments are based on the triggering of the luciferase gene following integration,^73^ and HIV RT inhibition has been ruled out (Fig. S1†), the compounds could potentially inhibit HIV integration, which could be further investigated.

## 3. Conclusion

Overall, novel 3-benzoylbenzofurans and pyrazole derivatives were successfully synthesized. Four compounds, including two 3-benzoylbenzofurans (3g and 4b) and two pyrazole derivatives (5f and 5h), were the most potent inhibitors of HIV-1 pseudovirus infections (Q23 and CAP210) with IC_50_ values less than 10 μM. Compounds 4b and 5f were the most potent inhibitors among the 3-benzoylbenzofurans and pyrazole derivatives, respectively. Compounds 3g and 5h were mild inhibitors of HIV-1 protease, while pyrazole (5f) was the most potent HIV PR inhibitor. The 3-benzoylbenzofurans produced in this study were shown to be cytotoxic, and derivatizing them to pyrazoles decreased their cytotoxicity while maintaining their potent anti-HIV activity. Additionally, there was no significant difference in anti-HIV activities against subtype A and C infections, which makes pyrazoles ideal HIV inhibitor since they have the potential to be effective against various HIV isolates. Together, these observations are compelling for the further development of these compounds for HIV management.

## 4. Experimental procedure

### 4.1 General procedures

The chemicals and reagents used for synthesis were bought from Sigma-Aldrich/Merck (Pty) Ltd. Radchem (Pty) Ltd. was used to source all the solvents. Ethyl acetate and hexanes, the solvents employed for chromatographic purification, were first distilled before use. Both the deuterated solvents for NMR and the reagent-grade solvents for synthesis were bought from Sigma-Aldrich. All reactions that required inert conditions were carried out under a nitrogen or argon atmosphere. Using suitable solvent mixtures, the progression of the reactions was monitored on the aluminium-backed silica gel 60 F_254_ TLC plates (Sigma Aldrich/Merck). The UV lamp (254 nm) and fluorescent lamp (365 nm) were used to view the TLC plates. The purification of the major intermediates and final products was conducted by column chromatography using silica gel (230–400 mesh with a particle size of 40–63 μM) and hexanes/ethyl acetate mixtures as eluent. The intermediates and final products were characterized using techniques that included mass spectrometry (MS), and Nuclear Magnetic Resonance (NMR) spectroscopy. The HRMS (ESI-QTOF) analysis was carried out using a Waters UPLC coupled to a QTOF Synapt G2 mass spectrometry. LRMS and the purity of the final compounds were measured on an Agilent 1260 Infinity HPLC with a PDA detector coupled to a Bruker AmaZon SL ion trap mass spectrometry. The NMR spectra of the compounds were recorded at room temperature using Bruker Avance III and Avance III HD spectrometers operating at 300, 400 and 500 MHz for ^1^H and 75, 100 and 125 MHz for ^13^C analyses. The spectra were processed with Bruker TopSpin 3.6.5 software and the chemical shifts were referenced against solvent peaks at *δ*_H_ 2.50 and *δ*_C_ 39.5 for DMSO-*d*_6_, *δ*_H_ 3.31 and *δ*_C_ 49.05 for methanol-*d*_4_ and, *δ*_H_ 7.26 and *δ*_C_ 77.16 for chloroform-*d*_1_.

#### 4.1.1 The synthesis of the 3-benzoylbenzofurans (GP1)

The 3-benzoylbenzofurans were synthesized as previously described,^26^ through the condensation of the 2-methoxyacetophenones (1 molar equiv) and excess DMF-DMA under inert conditions at 90 to 200 °C for 12 to 72 hours. Upon completion, the reaction mixture (enaminoketone) was cooled to room temperature and concentrated *in vacuo*. 1,4-Benzoquinone or 1,4-naphthoquinone derivatives (1.2 molar equiv) and excess acetic acid were added, and the reaction mixture was stirred at room temperature for 12 hours producing the 3-benzoylbenzofurans. The reaction was quenched using ice-cold water and the mixture was extracted using ethyl acetate (3 × 50 mL). The combined organic layers were washed with sodium chloride and dried with anhydrous magnesium sulfate or sodium sulfate. The crude products were purified using silica gel column chromatography and mixtures of hexanes/ethyl acetate as eluent. The benzoylbenzofurans 3a–3h were synthesized and characterized previously by Kunyane *et al.*^26^ The compounds 3g and 3h were re-synthesized in this study.

##### 4.1.1.1 (5-Hydroxybenzofuran-3-yl)(2,3,4-trimethoxyphenyl)methanone (3g)

The benzofuran 3g was synthesized from 2,3,4-trimethoxyacetophenone (0.61 g, 2.90 mmol) and 1,4-benzoquinone (0.38 g, 3.48 mmol) following GP1. Compound 3g was purified with column chromatography using hexanes/ethyl acetate (7 : 3). It was obtained as a white powder with a yield of 0.37 g (39%); mp 218–225 °C. ^1^H NMR (400 MHz, DMSO-*d*_6_) *δ* 3.74 (s, 3H), 3.79 (s, 3H), 3.87 (s, 3H), 6.85 (dd, *J* = 2.6 and 8.9 Hz, 1H), 6.92 (d, *J* = 8.7 Hz, 1H), 7.23 (d, *J* = 8.7 Hz, 1H), 7.47 (d, *J* = 2.6 Hz, 1H), 7.51 (d, *J* = 8.9 Hz, 1H), 8.36 (s, 1H), and 9.50 (s, 1H). ^13^C NMR (125 MHz, DMSO-*d*_6_) *δ* 56.0, 60.5, 61.7, 106.4, 107.4, 112.1, 114.3, 121.8, 124.0, 125.2, 127.2, 141.7, 149.2, 151.4, 154.8, 155.4, 155.6, and 188.5. HRMS (ESI/Q-TOF) *m*/*z* calcd. for C_18_H_17_O_6_ 329.1020 [M + H]^+^, found 329.1020.

##### 4.1.1.2 (5-Hydroxybenzofuran-3-yl)(2,5-dimethoxyphenyl)methanone (3h)

The benzofuran 3h was synthesized from 2,5-dimethoxyacetophenone (3.00 g, 16.65 mmol) and 1,4-benzoquinone (2.16 g, 19.98 mmol) following GP1. Compound 3h was purified with column chromatography using hexanes/ethyl acetate (7 : 3). It was obtained as a white powder with a yield of 0.61 g (12%); mp 173–180 °C. The ^1^H NMR (400 MHz, DMSO-*d*_6_) *δ* 3.69 (s, 3H), 3.74 (s, 3H), 6.85 (dd, *J* = 2.6 and 8.9 Hz, 1H), 6.97 (d, *J* = 2.9 Hz, 1H), 7.08 (dd, *J* = 2.9 and 9.0 Hz, 1H) 7.12 (d, *J* = 9.0 Hz, 1H), 7.46 (d, *J* = 2.6 Hz, 1H), 7.49 (d, *J* = 8.9 Hz, 1H), 8.33 (s, 1H) and 9.50 (s, 1H). The ^13^C NMR (125 MHz, DMSO-*d*_6_) *δ* 55.5, 56.0, 106.3, 112.0, 113.5, 113.6, 114.3, 116.8, 121.6, 124.9, 129.9, 149.1, 150.2, 152.8, 154.8, 155.9, and 189.3. HRMS (ESI/Q-TOF) *m*/*z* calcd. for C_17_H_15_O_5_ 299.0914 [M + H]^+^, found 299.0916.

#### 4.1.2 Methylation of 3-benzoylbenzofurans (**GP2**)

The 3-benzoylbenzofuran (1 molar equiv), methyl iodide (3 molar equiv), and potassium carbonate (3 molar equiv) were stirred at 100 °C and refluxed in DMF for 5–12 hours under inert conditions. Upon completion, the reaction was stopped with ice-cold water, followed by extraction and purification.

##### 4.1.2.1 (2,5-Dimethoxyphenyl)(5-methoxy-4,6-dimethyl-1-benzofuran-3-yl)methanone (4a)

Compound 4a was synthesized by methylating (2,5-dimethoxyphenyl)(5-hydroxy-4,6-dimethyl-1-benzofuran-3-yl)methanone (3a) (0.37 g, 1.15 mmol) according to GP2. Compound 4a was purified using column chromatography using hexanes/ethyl acetate (7 : 3). It was obtained as a yellow solid with a yield of 0.28 g (71%); mp 80–85 °C. ^1^H NMR (400 MHz, methanol-*d*_4_), *δ* 2.39 (s, 3H), 2.62 (s, 3H), 3.70 (s, 3H), 3.74 (s, 3H), 3.78 (s, 3H), 7.01 (d, *J* = 3.2 Hz, 1H), 7.06–7.11 (m, 2H), 7.23 (s, 1H), and 7.87 (s, 1H). ^13^C NMR (125 MHz, methanol-*d*_4_), *δ* 15.2, 17.5, 56.8, 57.3, 61.2, 112.1, 115.1, 116.3, 119.3, 124.3, 126.9, 127.1, 131.7, 132.5, 153.6, 154.6, 155.4, 155.9, 156.7, and 192.0. HRMS (ESI/Q-TOF) *m*/*z* calcd. for C_20_H_21_O_5_ 341.1384 [M + H]^+^, found *m*/*z* was 341.1389.

##### 4.1.2.2 (2,5-Dimethoxyphenyl)(5-methoxy-4,7-dimethyl-1-benzofuran-3-yl)methanone (4b)

The benzofuran 4b was synthesized by methylating (2,5-dimethoxyphenyl)(5-hydroxy-4,7-dimethyl-1-benzofuran-3-yl)methanone (3b) (0.30 g, 0.92 mmol) according to GP2. Compound 4b was purified using column chromatography using hexanes/ethyl acetate (7 : 3). It was obtained as a yellow sticky solid with a yield of 0.30 g (96%); mp 59–61 °C. ^1^H NMR (400 MHz, methanol-*d*_4_), *δ* 2.44 (s, 3H), 2.49 (s, 3H), 3.67(s, 3H), 3.76 (s, 3H), 3.84 (s, 3H), 6.87 (s, 1H), 6.99 (d, *J* = 2.8 Hz, 1H), 7.02–7.08 (m, 2H) and 7.88 (s, 1H). The ^13^C NMR (125 MHz, methanol-*d*_4_), *δ* 13.9, 14.9, 56.3, 56.8, 57.5, 112.9, 114.6, 115.9, 118.8, 119.1, 120.2, 125.2, 126.6, 132.0, 151.5, 153.1, 154.8, 156.0, 156.8, and 191.7. HRMS (ESI/Q-TOF) *m*/*z* calcd. for C_20_H_21_O_5_ 341.1384 [M + H] ^+^, found 341.1385.

##### 4.1.2.3 (2,5-Dimethoxyphenyl)(5-methoxynaphtho[1,2-*b*]furan-3-yl)methanone (4c)

The benzofuran 4c was synthesized by methylating compound 3c (0.30 g, 0.86 mmol) according to GP2. Compound 4c was purified using column chromatography using hexanes/ethyl acetate (7 : 3). It was obtained as a cream white solid with a yield of 0.29 g (94%); mp 140–149 °C. ^1^H NMR (400 MHz, methanol-*d*_4_), *δ* 3.76 (s, 3H), 3.81 (s, 3H), 4.07 (s, 3H), 7.03–7.04 (m, 1H), 7.09–7.13 (m, 2H), 7.53–7.57 (m, 2H), 7.66 (brt, *J* = 7.7 Hz, 1H), 8.19–8.22 (m, 2H), and 8.34 (brd, *J* = 8.3 Hz, 1H). ^13^C NMR (125 MHz, methanol-*d*_4_), *δ* 56.36, 56.42, 56.8, 97.9, 114.6, 115.4, 118.3, 120.6, 121.5, 122.7, 124.2, 125.0, 125.9, 126.5, 128.6, 131.7, 147.6, 152.5, 154.9, 155.0, 155.1, and 192.6. HRMS (ESI/Q-TOF) *m*/*z* calcd. for C_22_H_19_O_5_ 363.1227 [M + H] ^+^, found 363.1249.

##### 4.1.2.4 (5-Bromo-2-methoxyphenyl)(5-methoxynaphtho[1,2-*b*]furan-3-yl)methanone (4d)

Compound 4d was synthesized by methylating (5-bromo-2-methoxyphenyl)(5-hydroxynaphtho[1,2-*b*]furan-3-yl)methanone (3d) (0.09 g, 0.227 mmol) according to GP2. Compound 4d was purified using column chromatography using hexanes/ethyl acetate (7 : 3). It was obtained as white crystals with a yield of 0.09 g (96%); mp 156–162 °C. ^1^H NMR (400 MHz, methanol-*d*_4_), *δ* 3.81 (s, 3H), 4.08 (s, 3H), 7.16 (d, *J* = 8.8 Hz, 1H), 7.56–7.58 (m, 3H), 7.65–7.69 (m, 2H), 8.21–8.24 (m, 2H) and 8.33 (d, *J* = 8.3 Hz, 1H). ^13^C NMR (125 MHz, methanol-*d*_4_), *δ* 56.4, 56.5, 97.8, 113.6, 115.2, 120.6, 121.4, 122.6, 124.2, 124.8, 125.9, 126.6, 128.6, 132.4, 132.8, 135.9, 147.7, 155.0, 155.1, 157.6, and 191.0. HRMS (ESI/Q-TOF) *m*/*z* calcd. for C_21_H_16_B_r_O_4_ 411.0227 [M + H]^+^, found 411.0239.

##### 4.1.2.5 (4-Fluoro-2-methoxyphenyl)(5-methoxynaphtho[1,2-*b*]furan-3-yl)methanone (4e)

Compound 4e was synthesized by methylating (4-fluoro-2-methoxyphenyl)(5-hydroxynaphtho[1,2-*b*]furan-3-yl)methanone (3e) (0.15 g, 0.45 mmol) according to GP2. Compound 4e was purified using column chromatography using hexanes/ethyl acetate (7 : 3). It was obtained as white crystals with a yield of 0.15 g (95%); mp 133–138 °C. ^1^H NMR (300 MHz, methanol-*d*_4_), *δ* 3.78 (s, 3H), 4.04 (s, 3H), 6.93 (td, *J* = 8.4, 2.2 Hz, 1H), 7.14 (dd, *J* = 11.6, 2.2 Hz, 1H), 7.52–7.57 (m, 2H), 7.59–7.64 (m, 1H), 7.70–7.76 (m, 1H), and 8.21 (brd, *J* = 8.4 Hz, 1H), 8.28 (brd, *J* = 8.5 Hz, 1H), 8.53 (s, 1H). ^13^C NMR (75 MHz, methanol-*d*_4_), *δ* 56.0, 56.2, 96.9, 100.5–100.9 (d, ^2^*J*_CF_ = 26.1 Hz), 107.0–107.2 (d, ^2^*J*_CF_ = 21.8 Hz), 119.5, 120.2, 120.7, 122.9, 123.1, 123.7, 125.8, 125.9 (d, ^4^*J*_CF_ = 3.1 Hz), 128.0, 130.7–130.8 (d, ^3^*J*_CF_ = 11.0 Hz), 145.4, 152.9, 154.4, 158.6–158.7 (d, ^3^*J*_CF_ = 10.8 Hz), 162.9–166.1 (d, ^1^*J*_CF_ = 247.2 Hz), and 189.0. HRMS (ESI/Q-TOF) *m*/*z* calcd. for C_21_H_16_FO_4_ 351.1027 [M + H]^+^, found 351.1029.

#### 4.1.3 Synthesis of the pyrazole derivatives from the 3-benzoylbenzofurans (**GP3**)

The 3-benzoylbenzofuran (1 molar equiv) and hydrazine hydrate (4 molar equiv) were dissolved in methanol and stirred at room temperature for 2 to 5 hours.^32^ Upon completion, the product was concentrated to remove methanol, and washed with distilled hexanes or ethyl acetate.

##### 4.1.3.1 2-[3-(2,5-Dimethoxyphenyl)-1*H*-pyrazol-4-yl]-4-methoxy-3,5-dimethylphenol (5a)

The pyrazole 5a was synthesized by reacting compound 4a (0.20 g, 0.59 mmol) with hydrazine hydrate (0.12 g, 2.35 mmol) according to GP3. Compound 5a was obtained as white crystals with a yield of 0.15 g (72%); mp 111–123 °C. ^1^H NMR (400 MHz, methanol-*d*_4_), *δ* 1.81 (s, 3H), 2.22 (s, 3H), 3.46 (s, 3H), 3.57 (s, 3H), 3.74 (s, 3H), 6.57 (s, 1H), 6.76 (d, *J* = 3.2 Hz, 1H), 6.79 (dd, *J* = 3.2 and 8.9 Hz, 2H), 6.93 (d, *J* = 8.9 Hz, 1H) and 7.45 (s, 1H). ^13^C NMR (100 MHz, methanol-*d*_4_), *δ* 13.8, 16.3, 55.7, 56.4, 60.4, 113.6, 114.8, 115.7, 115.8, 116.0, 116.2, 119.6, 120.5, 131.7, 132.4, 141.3, 151.3, 152.1, 152.6, and 154.8. HRMS (ESI/Q-TOF) *m*/*z* calcd. for C_20_H_23_N_2_O_4_ was 355.1653 [M + H]^+^, found 355.1655.

##### 4.1.3.2 2-[3-(2,5-Dimethoxyphenyl)-1*H*-pyrazol-4-yl]-4-methoxy-3,6-dimethylphenol (5b)

The pyrazole 5b was synthesized by reacting benzofuran 4b (0.20 g, 0.59 mmol) with hydrazine hydrate (0.12 g, 2.35 mmol) according to GP3. Compound 5b was obtained as white crystals with a yield of 0.18 g (86%); mp 133–146 °C. ^1^H NMR (300 MHz, methanol-*d*_4_), *δ* 1.72 (s, 3H), 2.22 (s, 3H), 3.45 (brs, 3H), 3.72 (s, 6H), 6.70 (s, 1H), 6.76 (brs, 1H), 6.80 (dd, *J* = 8.9, 3.0 Hz, 1H), 6.94 (brd, *J* = 8.9 Hz, 1H) and 7.45 (brs, 1H). ^13^C NMR (75 MHz, methanol-*d*_4_), *δ* 13.3, 16.8, 55.7, 56.4, 56.6, 113.6, 114.2, 114.6, 115.2, 116.6, 119.9, 122.9, 123.2, 125.4, 138.9, 141.4, 148.1, 152.0, 152.5, and 154.8. HRMS (ESI/Q-TOF) *m*/*z* calcd. for C_20_H_23_N_2_O_4_ was 355.1653 [M + H]^+^, found 355.1653.

##### 4.1.3.3 2-[3-(2,5-Dimethoxyphenyl)-1*H*-pyrazol-4-yl]-4-methoxynaphthalen-1-ol (5c)

The pyrazole 5c was synthesized by reacting benzofuran 4c (0.20 g, 0.55 mmol) with hydrazine hydrate (0.11 g, 2.21 mmol) according to GP3. Compound 5c was obtained as brownish-red crystals with a yield of 0.18 g (88%); mp 175–179 °C. ^1^H NMR (400 MHz, methanol-*d*_4_), *δ* 3.49 (brs, 3H), 3.56 (brs, 3H), 3.64 (s, 3H), 6.47 (s, 1H), 6.85–6.95 (m, 3H), 7.39–7.48 (m, 2H), 7.89 (brs, 1H), 8.09 (brd, *J* = 8.3 Hz, 1H), and 8.18 (brd, *J* = 8.3 Hz, 1H). ^13^C NMR (100 MHz, methanol-*d*_4_), *δ* 55.8, 55.9, 56.3, 107.6, 113.7, 116.0, 116.2, 117.0, 118.0, 122.6, 123.1, 126.0, 126.7, 126.8, 128.2, 133.3, 134.9, 139.4, 144.0, 150.0, 152.7, and 154.9. HRMS (ESI/Q-TOF) *m*/*z* calcd. for C_22_H_21_N_2_O_4_ 377.1496 [M + H]^+^, found 377.1496.

##### 4.1.3.4 2-[3-(5-Bromo-2-methoxyphenyl)-1*H*-pyrazol-4-yl]-4-methoxynaphthalen-1-ol (5d)

The pyrazole 5d was synthesized by reacting compound 4d (0.06 g, 0.15 mmol) with hydrazine hydrate (0.03 g, 0.58 mmol) according to GP3. The product was obtained as a brownish-red powder with a yield of 0.06 g (91%); mp 155–161 °C. ^1^H NMR (400 MHz, methanol-*d*_4_), *δ* 3.48 (s, 3H), 3.63 (s, 3H), 6.42 (s, 1H), 6.91 (d, *J* = 8.7 Hz, 1H), 7.37–7.49 (m, 4H), 7.95 (s, 1H), 8.07 (brd, *J* = 8.3 Hz, 1H) and 8.17 (brd, *J* = 8.7 Hz, 1H). ^13^C NMR (75 MHz, methanol-*d*_4_), *δ* 55.9, 56.0, 107.3, 113.4, 114.3, 115.9, 118.5, 122.7, 123.1, 126.0, 126.8, 128.3, 133.3, 133.5, 133.6, 134.4, 134.6, 143.8, 150.1, and 158.0. HRMS (ESI/Q-TOF) *m*/*z* calcd. for C_20_H_18_BrN_2_O_3_, 425.0494 [M + H]^+^, found 425.0493.

##### 4.1.3.5 2-[3-(4-Fluoro-2-methoxyphenyl)-1*H*-pyrazol-4-yl]-4-methoxynaphthalen-1-ol (5e)

The pyrazole 5e was synthesized by reacting compound 4e (0.10 g, 0.29 mmol) with hydrazine hydrate (0.12 g, 2.35 mmol) according to GP3. Compound 5e was washed with hexanes and recrystallized from methanol and the pure product was obtained as a yellow powder with a yield of 0.10 g (95%); mp 123–127 °C. ^1^H NMR (300 MHz, chloroform-*d*_1_), *δ* 3.84 (s, 3H), 3.86 (s, 3H), 6.48 (td, *J* = 8.1, 2.5 Hz, 1H), 6.53 (s, 1H), 6.69 (dd, *J* = 10.2, 2.5 Hz, 1H), 6.98 (brs, 2H), 7.51–7.52 (m, 2H), 7.72 (s, 1H) and 8.18–8.22 (m, 2H). ^13^C NMR (125 MHz, chloroform-*d*_1_), *δ* 55.9, 56.2, 99.8–100.0 (d, ^2^*J*_CF_ = 26.4 Hz), 106.2, 108.1–108.2 (d, ^2^*J*_CF_ = 21.4 Hz), 111.9, 114.0–114.1 (d, ^4^*J*_CF_ = 3.4 Hz), 114.6, 121.9, 122.4, 125.4, 126.0, 126.1, 126.2, 130.6 (d, ^3^*J*_CF_ = 10.2 Hz), 137.8, 139.8, 143.0, 149.2, 157.6–157.7 (d, ^2^*J*_CF_ = 9.8 Hz) and 162.5–164.5 (d, ^1^*J*_CF_ = 249.0 Hz). HRMS (ESI/Q-TOF) *m*/*z* calcd. for C_21_H_18_FN_2_O_3_ 365.1295 [M + H]^+^, found 365.1297.

##### 4.1.3.6 2-[3-(4-Fluoro-2-methoxyphenyl)-1*H*-pyrazol-4-yl]benzene-1,4-diol (5f)

The pyrazole 5f was synthesized by reacting (4-fluoro-2-methoxyphenyl)(5-hydroxy-1-benzofuran-3-yl)methanone (3f) (0.2 g, 0.70 mmol) with hydrazine hydrate (0.14 g, 2.79 mmol) according to GP3. The product was obtained as cream white crystals with a yield of 0.13 g (61%); mp 255–258 °C. ^1^H NMR (400 MHz, chloroform-*d*_1_), *δ* 3.96 (s, 3H), 6.65 (ddd, *J* = 2.4, 7.7 and 8.8 Hz, 1H), 6.65–6.76 (m, 1H), 6.82 (dd, *J* = 2.4 and 10.4 Hz, 1H), 6.83–6.93 (m, 2H), 7.29 (dd, *J* = 6.4 and 8.9, 1H), and 8.14 (brs, 1H). ^13^C NMR (100 MHz, chloroform-*d*_1_), *δ* 56.3, 100.4–100.7 (d, ^2^*J*_CF_ = 26.6 Hz), 108.8–109.0 (d, ^2^*J*_CF_ = 22.1 Hz), 109.6 (d, ^4^*J*_CF_ = 4.2 Hz), 117.0, 117.4, 118.4, 118.6, 131.7–131.8 (d, ^3^*J*_CF_ = 10.9 Hz), 134.5, 141.2, 147.4, 148.4, 148.8, 158.8–158.9 (d, ^3^*J*_CF_ = 10.3 Hz), and 164.7–167.7 (d, ^1^*J*_CF_ = 255 Hz). HRMS (ESI/Q-TOF) *m*/*z* calcd. for C_16_H_14_FN_2_O_3_ 301.0983 [M + H]^+^, found 301.0984.

##### 4.1.3.7 2-[3-(2,3,4-Trimethoxyphenyl)-1*H*-pyrazol-4-yl]benzene-1,4-diol (5g)

The pyrazole (5g) was synthesized by reacting compound 3g (0.10 g, 0.31 mmol) with hydrazine hydrate (0.06 g, 1.22 mmol) according to GP3. Compound 5g was purified using column chromatography using hexanes/ethyl acetate (4 : 6). It was obtained as pale pink crystals with a yield of 0.02 g (16%); 141–148 °C. ^1^H NMR (400 MHz, methanol-*d*_4_), *δ* 3.57 (s, 3H), 3.81 (s, 3H), 3.85 (s, 3H), 6.46 (d, *J* = 3.0 Hz, 1H), 6.52 (dd, *J* = 8.7 and 3.0 Hz, 1H), 6.67 (d, *J* = 8.7 Hz, 1H), 6.76 (d, *J* = 8.7 Hz, 1H), 6.97 (d, *J* = 8.7 Hz, 1H) and 7.80 (s, 1H). ^13^C NMR (125 MHz, methanol-*d*_4_), *δ* 56.5, 61.2, 61.3, 108.6, 115.3, 117.3, 117.6, 118.0, 122.5, 126.8, 137.8, 141.4, 143.5, 148.8, 150.9, 153.1, and 155.5. HRMS (ESI/Q-TOF) *m*/*z* calcd. for C_18_H_19_N_2_O_5_ 343.1289 [M + H]^+^, found 343.1287.

##### 4.1.3.8 2-[3-(2,5-Dimethoxyphenyl)-1*H*-pyrazol-4-yl]benzene-1,4-diol (5h)

Compound 5h was synthesized by reacting compound 3h (0.20 g, 0.67 mmol) with hydrazine hydrate (0.13 g, 2.68 mmol) according to GP3. 5h was purified and obtained as white crystals with a yield of 0.15 g (69%); mp 210–213 °C. ^1^H NMR (300 MHz, chloroform-*d*_1_), *δ* 3.51 (s, 3H), 3.93 (s, 3H), 6.72–6.73 (m, 1H), 6.86–6.87 (m, 2H), 6.92 (d, *J* = 1.7 Hz, 1H), 7.02–7.03 (m, 2H), and 8.11 (s, 1H). ^13^C NMR (75 MHz, chloroform-*d*_1_), *δ* 55.8, 56.3, 113.0, 113.6, 114.4, 117.1, 117.3, 117.8, 117.9, 118.2, 119.5, 134.2, 140.9, 147.4, 149.4, 151.4 and 153.2. HRMS (ESI/Q-TOF) *m*/*z* calcd. for C_17_H_17_N_2_O_4_ 313.1183 [M + H]^+^, found 313.1188.

### 4.2 Biological assays

#### 4.2.1 Reagents

All cell culture reagents and cell growth medium were purchased from Sigma-Aldrich/Merck. The cell lines are TZM-bl cells (HeLa cells derived cell that is genetically engineered to contain CD4 and CCR5/CXCR4 receptor) were obtained from NIH AIDS Reagent Program and HEK 293T (derived from human embryonic kidney cells) were received as donations from the lab of Dr Patience Mthunzi, Biophotonics laboratory, CSIR. Compound stock solutions were prepared in DMSO, and the working concentrations in media contained less than 1% DMSO therefore the effects of DMSO were negligible. The cells were cultured in T-75 culture flasks (Lasec SA (Pty) Ltd Johannesburg) in Dulbecco's modified Eagle's medium (DMEM) with 3.7 g L^−1^ NaHCO_2_, 0.05% (v/v) gentamicin/mL, 1% penicillin/streptomycin and 10% heat-inactivated fetal bovine serum (Celtic Molecular Diagnostics). All incubations for tissue cell culture were conducted in a 37 °C, 5% CO_2_, and 95% humidity incubator.

#### 4.2.2 Cytotoxicity assay

The cytotoxicity of compounds was evaluated in TZM-bl cells since these cells are susceptible to HIV infections and would be used for the evaluation of HIV entry inhibition by 3-benzoylbenzofurans and pyrazole derivatives.^74^ MTT dye (3-(4,5-dimethylthiazol-2-yl)-2,5-diphenyltetrazolium bromide), which is reduced by viable cells to produce soluble purple formazan, was then measured and correlated with cell viability.^75^ TZM-bl cells were cultured to confluency in DMEM containing 10% FBS and harvested by centrifugation at 1200 × *g* for 5 minutes. The cells were counted at 1 × 10^5^ cells per mL per well and were seeded overnight, followed by treatment with serially diluted compounds from 500 to 0.98 μM for 48 hours. The peripheral blood mononuclear cells (PBMCs) were isolated from the blood of HIV-negative volunteers, treated with phytohaemagglutinin-protein (PHA, 4 μg mL^−1^) to stimulate the cells for 2 hours. PBMCs were diluted in RPMI-1640 medium, counted and 1 × 10^6^ cells per mL were treated with compounds (500 to 0.98 μM) for 48 hours. Cells were harvested by centrifugation at 1200 × *g* for 5 minutes, and the supernatants were removed. For the PBMCs, MTS dye (3-(4,5-dimethylthiazol-2-yl)-5-(3-carboxymethoxyphenylo)-2-(4-sulfophenyl)-2*H*-tetrazolium) was used.^76^ A 100 μL of tetrazolium dye (5 mg mL^−1^ MTT prepared in DMEM or 1 : 9 dilution MTS prepared in RPMI-1640) was added to the wells and incubated for 4 to 24 hours. MTT samples were solubilized with 100 μL solubilization buffer (1 mL HCl: 9 mL isopropanol) for 30 minutes, and the absorbance reading was taken at 550 nm and 690 nm wavelengths. MTS sample readings were taken at 490 nm without a solubilization step using the SpectraMax^R^ Paradigm^R^ Multi-Mode Detection Platform plate reader (Molecular Devices, California, USA).

#### 4.2.3 Pseudovirus production

The HIV pseudoviruses were produced from the plasmids that were kindly donated by Dr Mthunzi-Kufa (CSIR, South Africa). Competent DH5α cells and STBL2 cells were transformed with envelope (CAP210.2.00.E8 and Q23.17) and backbone plasmid (HIV-1 SG3 ΔEnv) through heat-shock at 42 °C for 90 seconds and cooled in ice for 5 minutes. The transformed cells were grown in Luria Bertani medium containing 100 μg mL^−1^ ampicillin and glycerol stocks were prepared. The transformed cells were grown at 37 °C in a 200 rpm shaking incubator overnight and the plasmids were isolated from the cells using a GeneJET plasmid Miniprep Kit (ThermoFisher Scientific Inc). The purity of the isolated plasmids was assessed for purity using Nanodrop. The HEK 293T cells were co-transfected with the backbone (10 μg) and envelope plasmids (5 μg) using the PolyFect Transfection Reagent (QIAGEN N.V) for 8–12 hours and the transfected cells were grown for 48 hours producing pseudoviruses which were collected, and stocks stored at −80 °C.

#### 4.2.4 HIV-1 whole virus inhibition assay

The compounds were serial diluted from 100 to1.56 μM in 96 well plates (Separation Scientific SA (Pty) Ltd) and incubated with either CAP210 or Q23 pseudoviruses (200 TCID_50_) and incubated for 1 hour. The TZM-bl cells (1 × 10^5^ cells per mL containing 7.5 μg mL^−1^ DEAE-dextran) were added and incubated for 48 hours. The 96 well plates were centrifuged, and the supernatant was removed leaving only 50 μL in each well. The Bright-Glo™ Luciferase substrate (Anatech Instruments (Pty) Ltd) (50 μL) was added to each well and incubated for 2 minutes in the dark and chemiluminescence readings were taken in a Corning® 96-well white polystyrene plate using a SpectraMax® Paradigm® Multi-Mode Microplate Reader.

#### 4.2.5 Time of drug addition assay

TZM-bl cells (1 × 10^5^ cells per mL containing 7.5 μg mL^−1^ DEAE-dextran) were infected with CAP210 pseudovirus at 400 TCID_50_ and incubated at 37 °C for 48 hours. The compounds were added to the wells at various time points (0, 2, 4, 6, and 8 hours) post-infection. Compounds 3g and 4b were used at 3 μM, while 5f and 5h were used at 25 μM to ensure 100% inhibition of HIV infections by CAP210 pseudoviruses. After 48 hours, the cells were centrifuged at 1200 × *g* for 5 minutes, culture media was removed, leaving 50 μL, and Bright-Glo substrate (50 μL) was added to detect luciferase activity for 2 minutes at 37 °C. Inhibition of HIV pseudoviruses by active compounds was detected by reading for chemiluminescence using a Corning® 96-well white polystyrene using the SpectraMax® Paradigm® Multi-Mode Microplate Reader.

#### 4.2.6 HIV-1 protease inhibition assay

The inhibition of HIV-1 PR was assessed using HIV protease substrate 1 (Arg-Glu(EDANS)-Ser-Gln-Asn-Tyr-Pro-Ile-Val-Gln-Lys(DABCYL)-Arg) (Sigma-Aldrich/Merck). Compounds were serially diluted in protease assay buffer (0.1 M CH_3_COONa, 1 M NaCl, 1 mM EDTA, 1 mM DTT, and 1 mg mL^−1^ BSA; pH 4.7), making a final concentration of 75 to 9.4 μM, and 49 μL of compounds were added to wells. 49 μL protease substrate 1 (10 μM) was added to each well, and 2 μL recombinant HIV-PR (0.2 μg mL^−1^) was added only to reaction wells except for reaction blanks, where it was replaced with 2 μL assay buffer. The solutions were mixed and incubated at 37 °C for 1 hour. Endpoint fluorescence readings at an excitation wavelength of 370 nm and an emission wavelength of 520 nm. Acetyl-pepstatin (1.6 μM) was used as a positive control since it is a known inhibitor of HIV-PR, and each compound dilution had its blank since it was observed to produce fluorescence.

### 4.3 *In silico* analysis of 3-benzoylbenzofurans and pyrazole derivatives

#### 4.3.1 Docking of active compounds on HIV-1 protease active site

Maestro 13.1 (Schrödinger 2022-1) was used to dock in the HIV-1 PR crystal structure (PDB code: 1HIV). Acetyl pepstatin was used as a control for HIV-1 PR docking to validate the docking method. The crystal structures were downloaded from the protein database and prepared as follows: preprocessing which filled missing loops, deleted water beyond 8 Å, optimised p*K*_α_ to 7.4 ± 2, optimised overlapping hydrogen bonds and cleaned up the crystal structures. The grid was generated on the crystal structures using the grid generator icon which allows for picking the atom on the ligand and creating a grid around that site. The compounds and controls were prepared using ligand preparation on Schrodinger.

#### 4.3.2 Evaluation of drug-likeness of active compounds

The drug-likeness of compounds was evaluated using online bioinformatics tools; SwissADME (https://www.swissadme.ch/index.php#), ADMETlab (https://www.admetmesh.scbdd.com/), and pkCSM (https://www.biosig.lab.uq.edu.au/pkcsm/). ACD/Labs (Chemsketch) was used to draw the structure of compounds convert them to SMILES and insert them into the online bioinformatic tools. The Lipinski rule of 5 (ref. 69) and the Pfizer rule (3/75)^50^ were employed to determine the drug-likeness in this study.

### 4.4 Data analysis and statistics

The graphs were plotted using GraphPad Prism 7.0 software, as was the statistical analysis. The student *t*-test and one-way ANOVA were used for statistical analysis. A *p*-value of <0.05 is considered significantly different.

## Data availability

Data will be made available on request.

## Conflicts of interest

The authors declare that they have no known competing financial interests or personal relationships that could have appeared to influence the work reported in this paper.

## Supplementary Material

MD-OLF-D4MD00844H-s001
