# Supplementary material for: The synthesis and investigation of novel 3-benzoylbenzofurans and pyrazole derivatives for anti-HIV activity
Source: RSC Med Chem. 2025 Feb 5;16(5):2142–58. doi: 10.1039/d4md00844h (PMC11883425; doi:10.1039/d4md00844h)
Supplement: MD-016-D4MD00844H-s001 [file MD-016-D4MD00844H-s001.pdf]

Supplementary Information (SI) for RSC Medicinal Chemistry.
This journal is © The Royal Society of Chemistry 2025

Supplementary document

The synthesis and investigation of novel 3-benzoylbenzofurans and pyrazole derivatives for anti-HIV activity

Sinothile S. Khuzwayo[1], Mamoalosi A. Selepe[2], Debra Meyer[3] and Ntombenhle H. Gama[1]

[1] *Biochemistry Department, University of Pretoria, 2 Lynnwood road, Pretoria, 0002, South Africa.*

[2] *Chemistry Department, University of Pretoria, 2 Lynnwood road, Pretoria, 0002, South Africa.*

[3] *School of Natural and Applied Sciences, Sol Plaatje University, Kimberley, 8300, South Africa.*

*Correspondence to:

Ms Sinothile Khuzwayo: snothilesementha81@gmail.com

Table of Contents

Figures

Fig. S1: Inhibition of HIV-1 RT by active compounds. Doxorubicin (DOX) and nevirapine (NVP) were used as controls in four decreasing concentrations to test the validity of the kit. The bars represent the mean ± SEM obtained from three independent experiments……..….S5

Fig. S2: Crystal structure of HIV-1 PR with an inhibitor. The catalytic site of HIV-1 protease showing a ligand and the amino acid residues that makes up the active site. Created using Discovery Studio 2024…………………………………………………………………..……S5

Tables

Table S1: Analysis of drug-likeness of 3-benzoylbenzofurans and pyrazole derivatives according to the Lipinski and Pfizer rule………………………………………………………..S6

^{1}H and ^{13}C NMR spectrum

Plate 1a: ^{1}H NMR (400 MHz, Methanol-d_4) spectrum for (2,5-dimethoxyphenyl)(5-methoxy-4,7-dimethyl-1-benzofuran-3-yl)methanone (4a) S6
Plate 1b: ^{13}C NMR (125 MHz, Methanol-d_4) spectrum for (2,5-dimethoxyphenyl)(5-methoxy-4,7-dimethyl-1-benzofuran-3-yl)methanone (4a)...S7
Plate 2a: ^{1}H NMR (400 MHz, Methanol-d_4) spectrum of (2,5-dimethoxyphenyl)(5-methoxy-4,6-dimethyl-1-benzofuran-3-yl)methanone (4b)...S7
Plate 2b: ^{13}C NMR (500 MHz, Methanol-d_4) spectrum of (2,5-dimethoxyphenyl)(5-methoxy-4,6-dimethyl-1-benzofuran-3-yl)methanone (4b)...S7
Plate 3a: ^{1}H NMR (400 MHz, Methanol-d_4) spectrum of (2,5-dimethoxyphenyl)(5-methoxynaphtho[1,2-*b*]furan-3-yl)methanone (4c)..S8
Plate 3b: ^{13}C NMR (125 MHz, Methanol-d_4) spectrum of (2,5-dimethoxyphenyl)(5-methoxynaphtho[1,2-*b*]furan-3-yl)methanone (4c)..S8
Plate 4a: ^{1}H NMR (400 MHz, Methanol-d_4) spectrum for (5-bromo-2-methoxyphenyl)(5-methoxynaphtho[1,2-*b*]furan-3-yl)methanone (4d)..S8
Plate 4b: ^{13}C NMR (125 MHz, Methanol-d_4) spectrum for (5-bromo-2-methoxyphenyl)(5-methoxynaphtho[1,2-*b*]furan-3-yl)methanone (4d)..S9
Plate 5a: ^{1}H NMR (300 MHz, Methanol-d_4) spectrum for (4-fluoro-2-methoxyphenyl)(5-methoxynaphtho[1,2-*b*]furan-3-yl)methanone (4e)..S9
Plate 5b: ^{13}C NMR (75 MHz, Methanol-d_4) spectrum for (4-fluoro-2-methoxyphenyl)(5-methoxynaphtho[1,2-b]furan-3-yl)methanone (4e)..S9
Plate 6a: ^{1}H NMR (400 MHz, DMSO-d_6) spectrum of (5-hydroxy-1-benzofuran-3-yl)(2,3,4-trimethoxyphenyl)methanone (3g)..S10
Plate 6b: ^{13}C NMR (100 MHz, DMSO-d_6) spectrum of (5-hydroxy-1-benzofuran-3-yl)(2,3,4-trimethoxyphenyl)methanone (3g)..S10

Plate 7a: ^{1}H NMR (400 MHz, DMSO-d_6) spectrum of (2,5-dimethoxyphenyl)(5-hydroxy-1-benzofuran-3-yl)methanone (3h)..S10
Plate 7b: ^{13}C NMR (125 MHz, DMSO-d_6) spectrum of (2,5-dimethoxyphenyl)(5-hydroxy-1-benzofuran-3-yl)methanone (3h)..S11
Plate 8a: ^{1}H NMR (400 MHz, Methanol-d_4) spectrum of 2-[3-(2,5-dimethoxyphenyl)-1*H*-pyrazol-4-yl]-4-methoxy-3,6-dimethylphenol (5a)..S11
Plate 8b: ^{13}C NMR (125 MHz, Methanol-d_4) spectrum of 2-[3-(2,5-dimethoxyphenyl)-1*H*-pyrazol-4-yl]-4-methoxy-3,6-dimethylphenol (5a)..S11
Plate 9a: ^{1}H NMR (400 MHz, Methanol-d_4) spectrum for 2-[3-(2,5-dimethoxyphenyl)-1*H*-pyrazol-4-yl]-4-methoxy-3,5-dimethylphenol (5b)..S12
Plate 9b: ^{13}C NMR (125 MHz, Methanol-d_4) spectrum of 2-[3-(2,5-dimethoxyphenyl)-1H-pyrazol-4-yl]-4-methoxy-3,5-dimethylphenol (5b)..S12
Plate 10a: ^{1}H NMR (400 MHz, Methanol-d_4) spectrum of 2-[3-(2,5-dimethoxyphenyl)-1H-pyrazol-4-yl]-4-methoxynaphthalen-1-ol (5c)..S12
Plate 10b: ^{13}C NMR (100 MHz, Methanol-d_4) spectrum for 2-[3-(2,5-dimethoxyphenyl)-1H-pyrazol-4-yl]-4-methoxynaphthalen-1-ol (5c)..S13
Plate 11a: ^{1}H NMR (400 MHz, Methanol-d_4) spectrum of 2-[3-(5-bromo-2-methoxyphenyl)-1*H*-pyrazol-4-yl]-4-methoxynaphthalen-1-ol (5d)..S13
Plate 11b: ^{13}C NMR (75 MHz, Methanol-d_4) spectrum of 2-[3-(5-bromo-2-methoxyphenyl)-1*H*-pyrazol-4-yl]-4-methoxynaphthalen-1-ol (5d)…..S13
Plate 12a: The ^{1}H NMR (300 MHz, Chloroform-d_1) spectrum of 2-[3-(4-fluoro-2-methoxyphenyl)-1*H*-pyrazol-4-yl]-4-methoxynaphthalen-1-ol (5e)……..….........................S14
Plate 12b: The ^{13}C NMR (125 MHz, Chloroform-d_1) spectrum of 2-[3-(4-fluoro-2-methoxyphenyl)-1H-pyrazol-4-yl]-4-methoxynaphthalen-1-ol (5e)…………………………..S14
Plate 13a: ^{1}H NMR (400 MHz, Chloroform-d_1) spectrum for 2-[3-(4-fluoro-2-methoxyphenyl)-1H-pyrazol-4-yl]benzene-1,4-diol (5f)…..S14
Plate 13b: ^{13}C NMR (100 MHz, Chloroform-d_1) spectrum for 2-[3-(4-fluoro-2-methoxyphenyl)-1H-pyrazol-4-yl]benzene-1,4-diol (5f). …...S15
Plate 14a: ^{1}H NMR (400 MHz, Methanol-d_4) spectrum for 2-[3-(4-ethyl-2,3-dimethoxyphenyl)-1H-pyrazol-4-yl]benzene-1,4-diol (5g). …...S15
Plate 14b: ^{13}C NMR (100 MHz, Methanol-d_4) spectrum for 2-[3-(2,3,4-trimethoxyphenyl)-1H-pyrazol-4-yl]benzene-1,4-diol (5g)…...S15
Plate 15a: The 1H NMR (300 MHz, Chloroform-d_1) spectrum for 2-[3-(2,5-dimethoxyphenyl)-1H-pyrazol-4-yl]benzene-1,4-diol (5h)……………………………………………………..S16
Plate 15b: ^{13}C NMR (75 MHz, Chloroform-d_1) spectrum for 2-[3-(2,5-dimethoxyphenyl)-1H-pyrazol-4-yl]benzene-1,4-diol (5h)…..S16

Chromatograms

Plate 1c: LC-MS chromatogram of (2,5-dimethoxyphenyl)(5-methoxy-4,7-dimethyl-1-benzofuran-3-yl)methanone (4a) measured at 280 nm..S17
Plate 1d: HR-MS spectrum of (2,5-dimethoxyphenyl)(5-methoxy-4,7-dimethyl-1-benzofuran-3-yl)methanone (4a)…..S17
Plate 2c: LC-MS chromatogram of (2,5-dimethoxyphenyl)(5-methoxy-4,6-dimethyl-1-benzofuran-3-yl)methanone (4b) measured at 280 nm. …..S18
Plate 2d: HR-MS spectrum of (2,5-dimethoxyphenyl)(5-methoxy-4,6-dimethyl-1-benzofuran-3-yl)methanone (4b). …...S18
Plate 3c: LC-MS chromatogram of (2,5-dimethoxyphenyl)(5-methoxynaphtho[1,2-*b*]furan-3-yl)methanone (4c)..…...S18
Plate 3d: HR-MS spectrum of (2,5-dimethoxyphenyl)(5-methoxynaphtho[1,2-*b*]furan-3-yl)methanone (4c)…..…...S19
Plate 4c: LC-MS chromatogram of (5-bromo-2-methoxyphenyl)(5-methoxynaphtho[1,2-*b*]furan-3-yl)methanone (4d) measured at 280 nm. …...S19

Plate 4d: HR-MS spectrum of (5-bromo-2-methoxyphenyl)(5-methoxynaphtho[1,2-*b*]furan-3-yl)methanone (4d). …..S19
Plate 5c: LC-MS chromatogram of (4-fluoro-2-methoxyphenyl)(5-methoxynaphtho[1,2-b]furan-3-yl)methanone (4e) measured at 280 nm...S20
Plate 5d: HR-MS spectrum of (4-fluoro-2-methoxyphenyl)(5-methoxynaphtho[1,2-b]furan-3-yl)methanone (4e)…...S20
Plate 6c: LC-MS chromatogram of (5-hydroxy-1-benzofuran-3-yl)(2,3,4-trimethoxyphenyl)methanone (3g) measured at 280 nm...S20
Plate 6d: HR-MS spectrum of (5-hydroxy-1-benzofuran-3-yl)(2,3,4-trimethoxyphenyl)methanone (3g)……………………………..……………………….S21
Plate 7c: LC-MS chromatogram of (2,5-dimethoxyphenyl)(5-hydroxy-1-benzofuran-3-yl)methanone (3h) measured at 280 nm...S21
Plate 7d: HR-MS spectrum of (2,5-dimethoxyphenyl)(5-hydroxy-1-benzofuran-3-yl)methanone (3h)…...S21
Plate 8c: LC-MS chromatogram of 2-[3-(2,5-dimethoxyphenyl)-1*H*-pyrazol-4-yl]-4-methoxy-3,6-dimethylphenol (5a) measured at 280 nm...S22
Plate 8d: HR-MS spectrum of 2-[3-(2,5-dimethoxyphenyl)-1*H*-pyrazol-4-yl]-4-methoxy-3,6-dimethylphenol (5a). …..S22
Plate 9c: UV chromatogram of 2-[3-(2,5-dimethoxyphenyl)-1H-pyrazol-4-yl]-4-methoxy-3,5-dimethylphenol (5b) measured at 280 nm……………………………………………………S22
Plate 9d: HR-MS spectrum for 2-[3-(2,5-dimethoxyphenyl)-1*H*-pyrazol-4-yl]-4-methoxy-3,5 dimethylphenol (5b)…..S23
Plate 10c: LC-MS chromatogram of 2-[3-(2,5-dimethoxyphenyl)-1*H*-pyrazol-4-yl]-4-methoxynaphthalen-1-ol (5c) measured at 260 nm...S23
Plate 10d: HR-MS spectrum for 2-[3-(2,5-dimethoxyphenyl)-1H-pyrazol-4-yl]-4-methoxynaphthalen-1-ol (5c)…..S23
Plate 11c: LC-MS chromatogram of 2-[3-(5-bromo-2-methoxyphenyl)-1*H*-pyrazol-4-yl]-4-methoxynaphthalen-1-ol (5d) measured at 260 nm..S24
Plate 11d: HR-MS spectrum of 2-[3-(5-bromo-2-methoxyphenyl)-1*H*-pyrazol-4-yl]-4-methoxynaphthalen-1-ol (5d)…..S24
Plate 12c: LC-MS chromatogram of 2-[3-(4-fluoro-2-methoxyphenyl)-1H-pyrazol-4-yl]-4-methoxynaphthalen-1-ol (5e)…..S24
Plate 12d: HR-MS spectrum of 2-[3-(4-fluoro-2-methoxyphenyl)-1*H*-pyrazol-4-yl]-4-methoxynaphthalen-1-ol (5e)…..S25
Plate 13c: LC-MS chromatogram of 2-[3-(4-fluoro-2-methoxyphenyl)-1H-pyrazol-4-5yl]benzene-1,4-diol (5f) measured at 280 nm……..S25
Plate 13d: HR-MS spectrum of 2-[3-(4-fluoro-2-methoxyphenyl)-1H-pyrazol-4-yl]benzene-1,4-diol (5f)…..S25
Plate 14c: LC-MS chromatogram of 2-[3-(2,3,4-trimethoxyphenyl)-1H-pyrazol-4-yl]benzene-1,4-diol (5g) at 280 nm...S26
Plate 14d: HR-MS spectrum of 2-[3-(2,3,4-trimethoxyphenyl)-1H-pyrazol-4-yl]benzene-1,4-diol (5g)…..S26
Plate 15c: LC-MS chromatogram for 2-[3-(2,5-dimethoxyphenyl)-1H-pyrazol-4-yl]benzene-1,4-diol (5h) measured at 280 nm..S26
Plate 15d: HR-MS spectrum for 2-[3-(2,5-dimethoxyphenyl)-1H-pyrazol-4-yl]benzene-1,4-diol (5h) measured at 280 nm..S27

1.1 Results

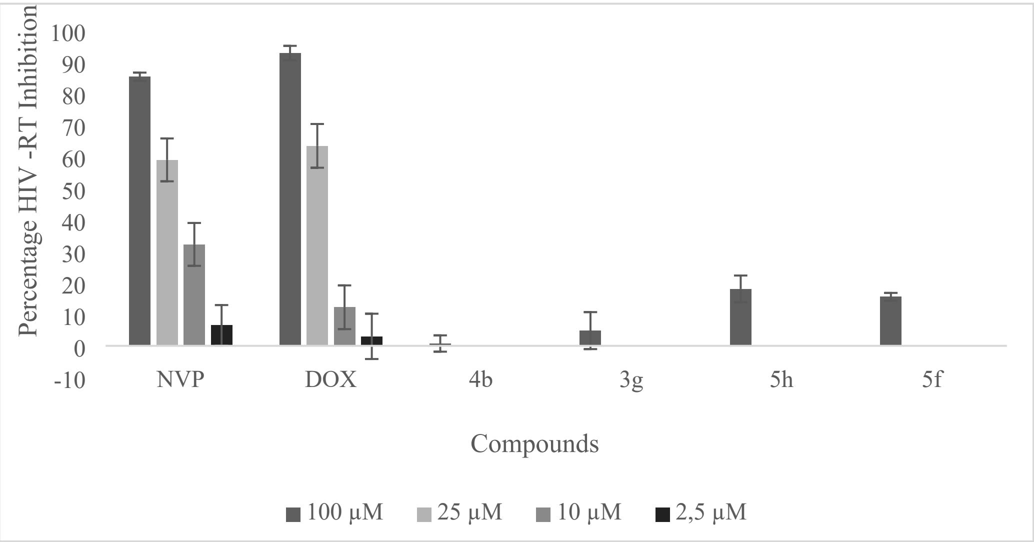

Fig. S1: Inhibition of HIV-1 RT by active compounds. Doxorubicin (DOX) and nevirapine (NVP) were used as controls in four decreasing concentrations to test the validity of the kit. The bars represent the mean ± SEM obtained from three independent experiments.

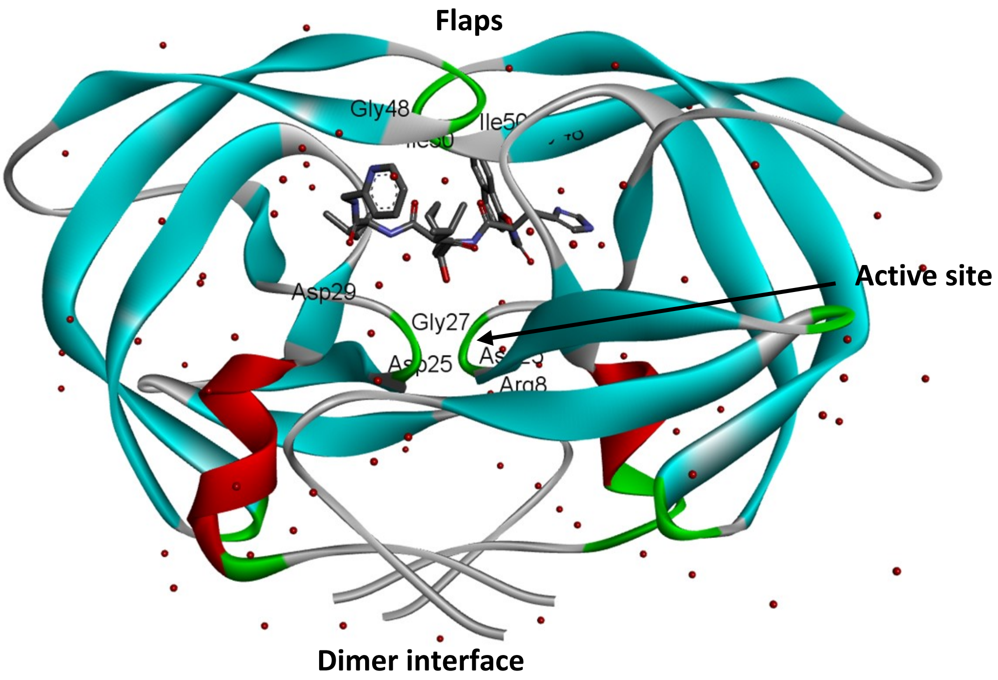

Fig. S2: Crystal structure of HIV-1 PR with an inhibitor. The catalytic site of HIV-1 protease showing a ligand and the amino acid residues that makes up the active site. Created using Discovery Studio 2024.

Table S1: Analysis of drug-likeness of 3-benzoylbenzofurans and pyrazole derivatives according to the Lipinski and Pfizer rule.

Compound	MW (g/mol)	MLogP	nHA	nHD	TPSA Å^2	Lipinski rule	Pfizer rule	PAINS alerts
**4b**	340.37	1.75	5	0	57.90	Yes	No	0
**5b**	354.40	1.78	5	2	76.60	Yes	Yes	0
**4d**	411.25	2.94	4	0	48.67	Yes	No	0
**5d**	425.28	2.96	4	2	67.37	Yes	No	0
**3f**	286.25	1.76	5	1	59.67	Yes	No	0
**5f**	300.28	1.78	5	3	78.37	Yes	Yes	0
**3g**	328.32	0.74	6	1	78.13	Yes	Yes	0
**5g**	342.35	0.79	6	3	96.83	Yes	Yes	0
**3h**	298.29	1.05	5	1	68.90	Yes	No	0
**5h**	312.32	1.08	5	3	87.60	Yes	Yes	0
**Saquenavir**	670.84	1.40	7	5	166.75	No	Yes	0
Lenacapavir	968.30	4.86	12	2	174.70	No	Yes	0

Molecular weight; MW; g/mol, Lipophilicity; MlogP, Number of hydrogen bond acceptors; nHA, Number of hydrogen bond donors; nHD.

1.2 Spectra and analysis of chromatograms

1.2.1 NMR Spectra

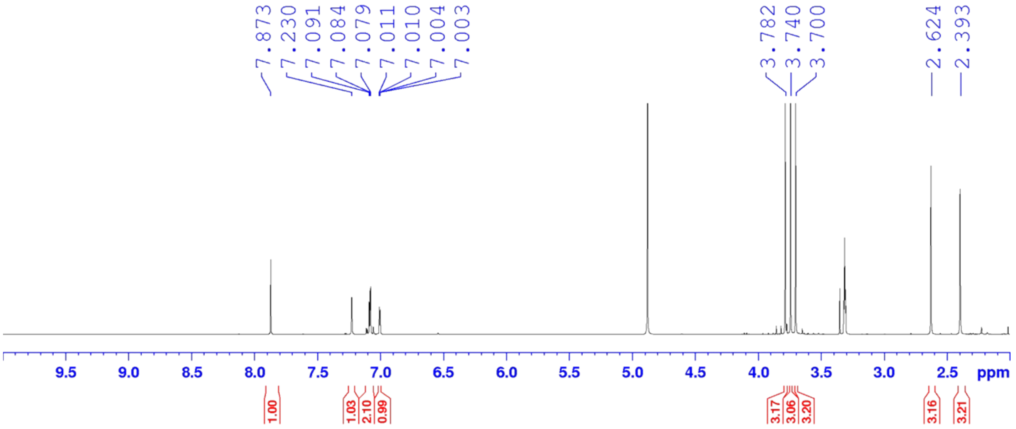

Plate 1a: The ^{1}H NMR (400 MHz, Methanol-*d*$_4$) spectrum for (2,5-dimethoxyphenyl)(5-methoxy-4,7-dimethyl-1-benzofuran-3-yl)methanone (4a).

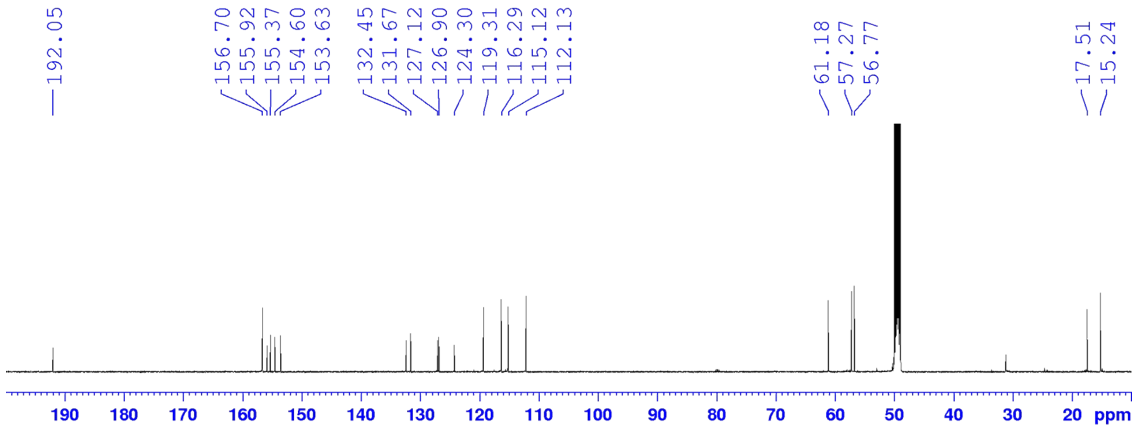

Plate 1b: The ^{13}C NMR (125 MHz, Methanol-d_4) spectrum for (2,5-dimethoxyphenyl)(5-methoxy-4,7-dimethyl-1-benzofuran-3-yl)methanone (4a).

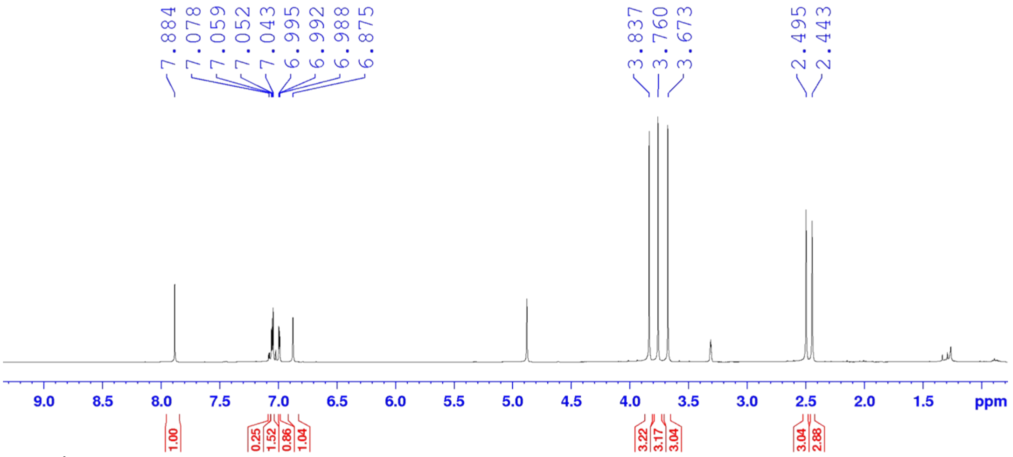

Plate 2a: The ^{1}H NMR (400 MHz, Methanol-d_4) spectrum of (2,5-dimethoxyphenyl)(5-methoxy-4,6-dimethyl-1-benzofuran-3-yl)methanone (4b).

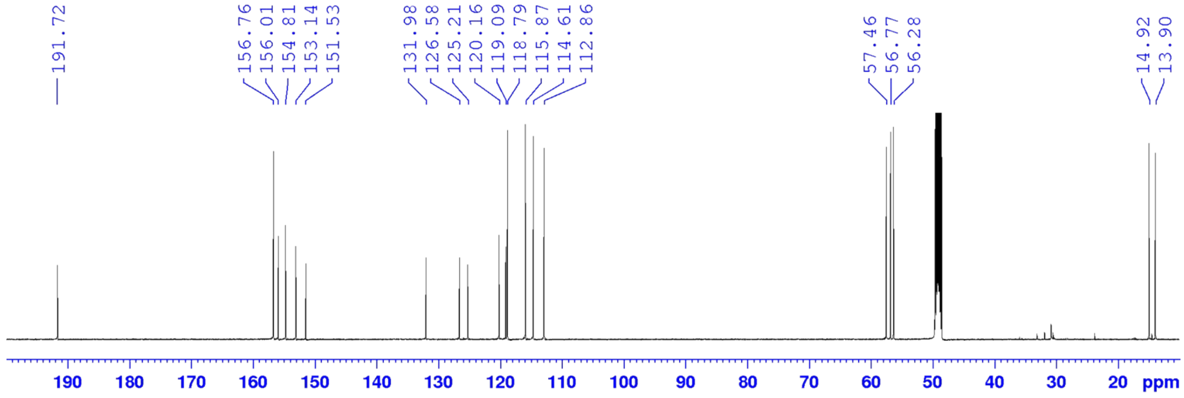

Plate 2b: The ^{13}C NMR (125 MHz, Methanol-d_4) spectrum of (2,5-dimethoxyphenyl)(5-methoxy-4,6-dimethyl-1-benzofuran-3-yl)methanone (4b).

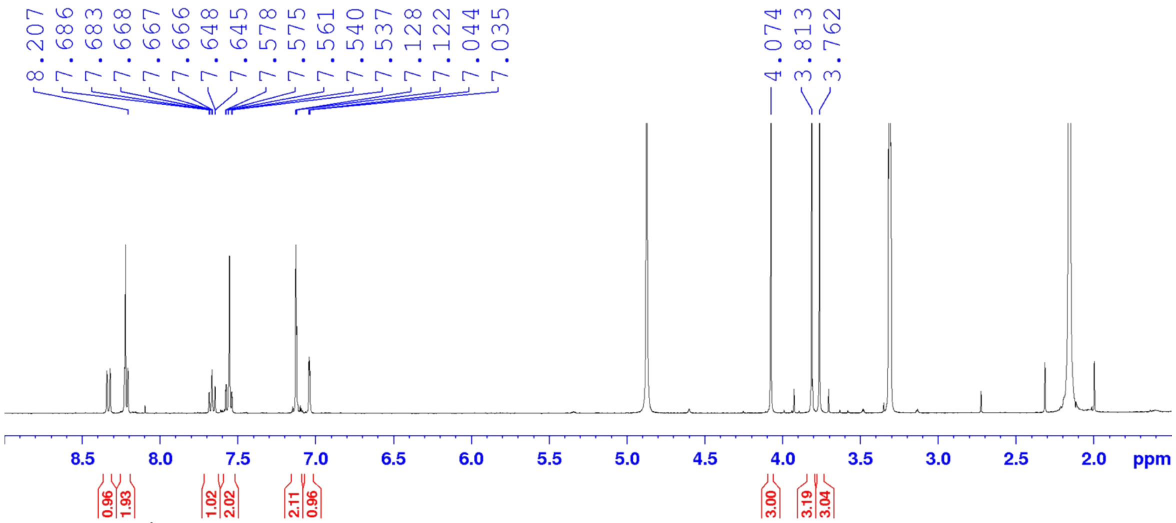

Plate 3a: The ^{1}H NMR (400 MHz, Methanol-d_4) spectrum of (2,5-dimethoxyphenyl)(5-methoxynaphtho[1,2-*b*]furan-3-yl)methanone (4c).

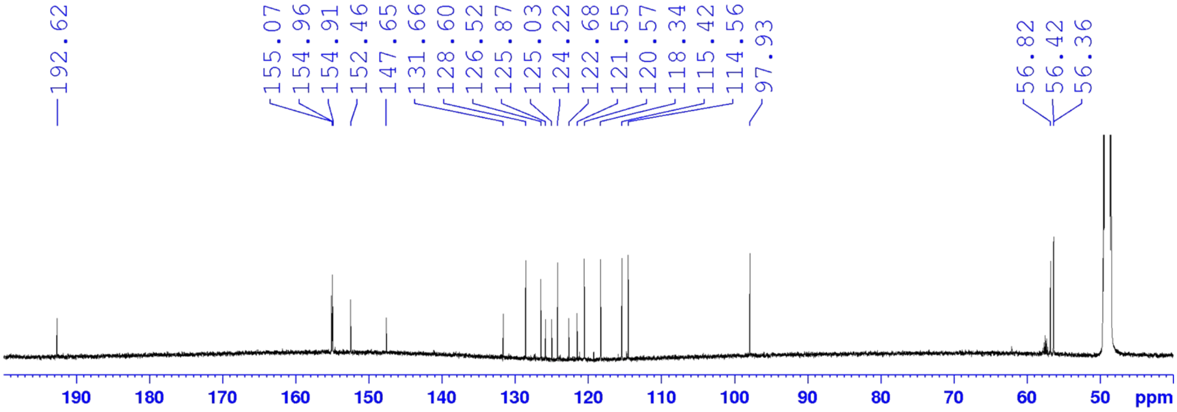

Plate 3b: The ^{13}C NMR (125 MHz, Methanol-d_4) spectrum of (2,5-dimethoxyphenyl)(5-methoxynaphtho[1,2-*b*]furan-3-yl)methanone (4c).

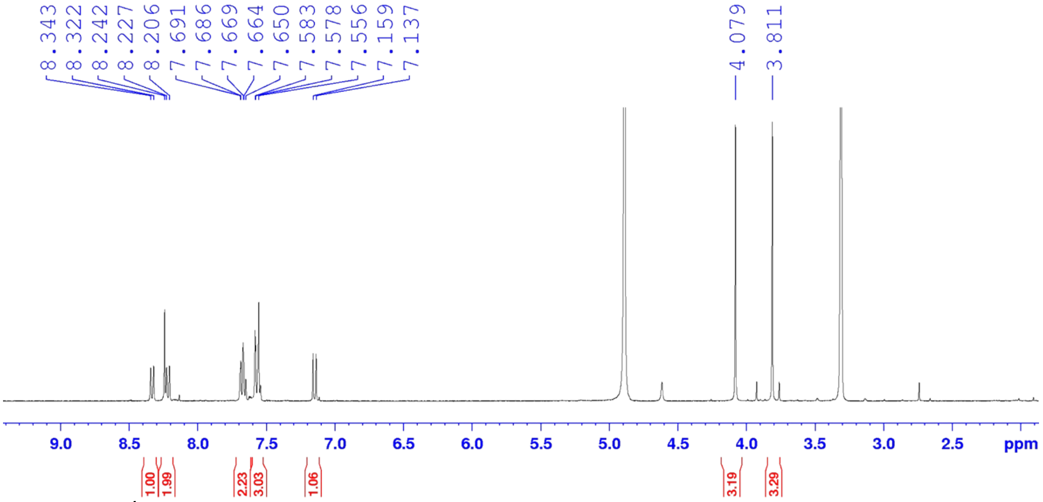

Plate 4a: The ^{1}H NMR (400 MHz, Methanol-d_4) spectrum for (5-bromo-2-methoxyphenyl)(5-methoxynaphtho[1,2-*b*]furan-3-yl)methanone (4d).

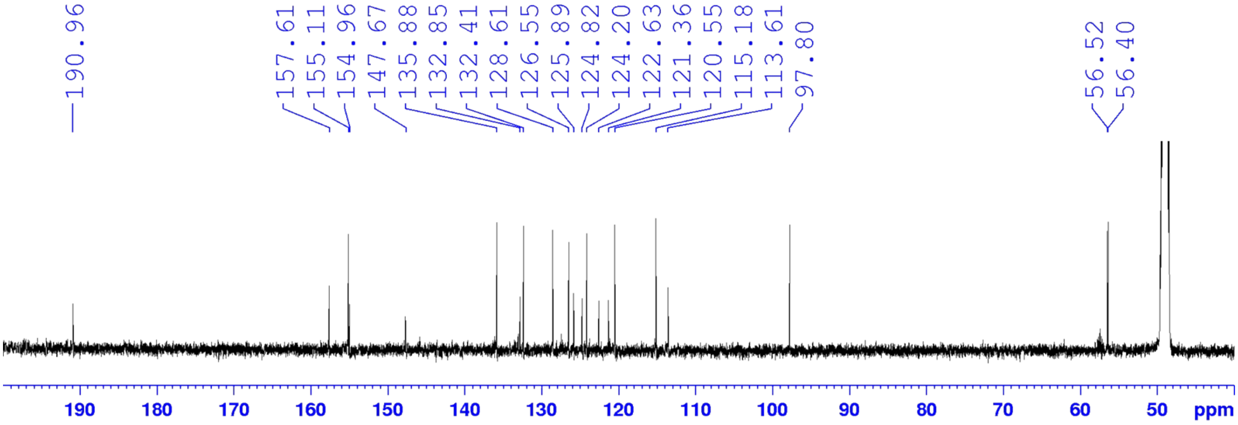

Plate 4b: The ^{13}C NMR (125 MHz, Methanol-d_4) spectrum for (5-bromo-2-methoxyphenyl)(5-methoxynaphtho[1,2-*b*]furan-3-yl)methanone (4d).

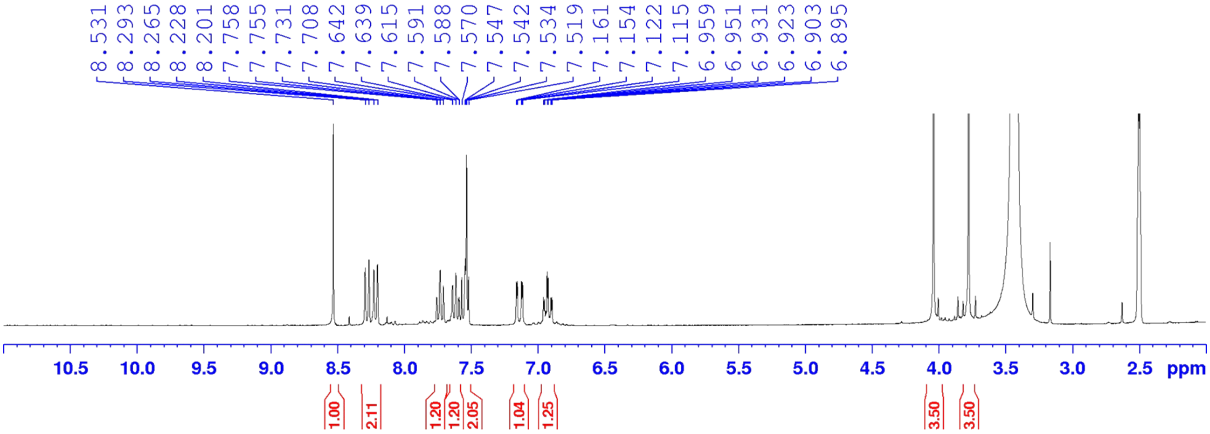

Plate 5a: The ^{1}H NMR (300 MHz, Methanol-d_4) spectrum for (4-fluoro-2-methoxyphenyl)(5-methoxynaphtho[1,2-*b*]furan-3-yl)methanone (4e).

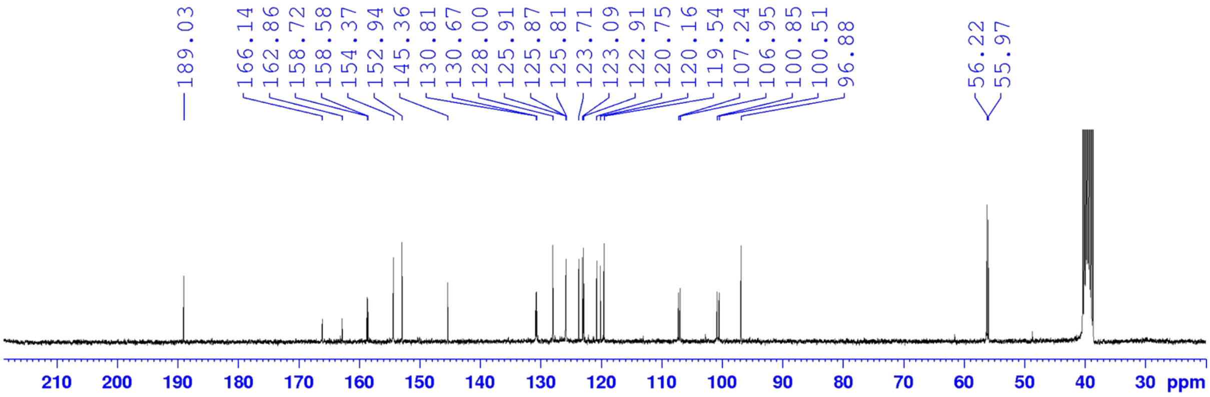

Plate 5b: The ^{13}C NMR (75 MHz, Methanol-d_4) spectrum for (4-fluoro-2-methoxyphenyl)(5-methoxynaphtho[1,2-b]furan-3-yl)methanone (4e).

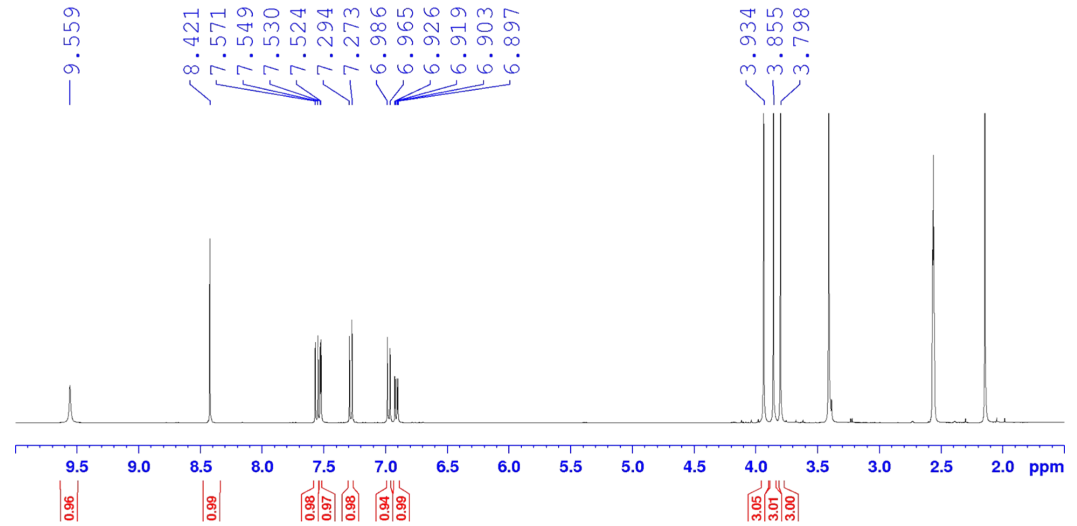

Plate 6a: The ^{1}H NMR (400 MHz, DMSO-d_6) spectrum of (5-hydroxy-1-benzofuran-3-yl)(2,3,4-trimethoxyphenyl)methanone (3g).

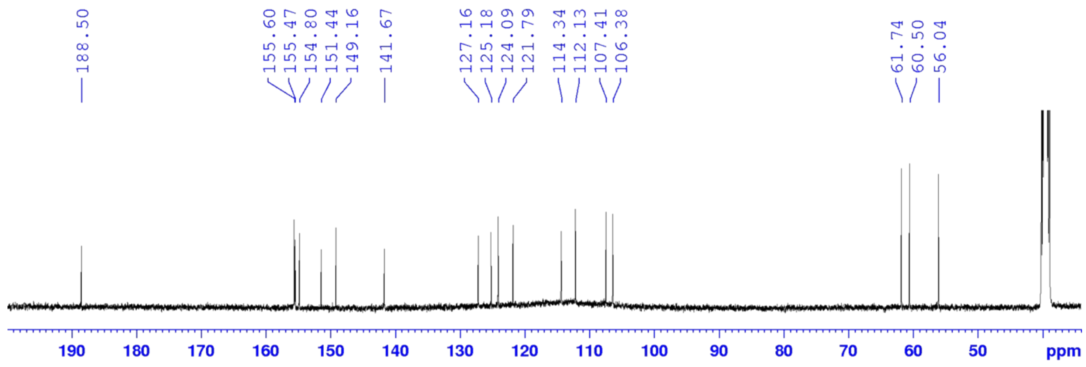

Plate 6b: The ^{13}C NMR (100 MHz, DMSO-d_6) spectrum of (5-hydroxy-1-benzofuran-3-yl)(2,3,4-trimethoxyphenyl)methanone (3g).

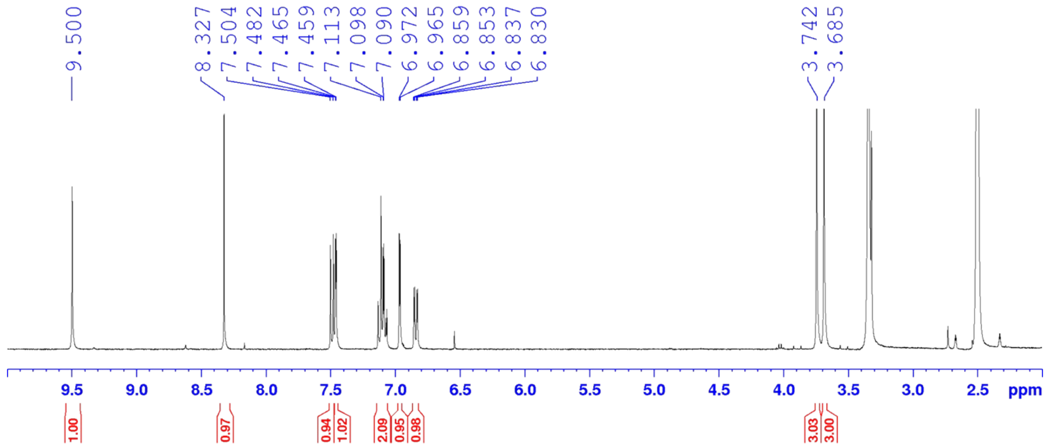

Plate 7a: The ^{1}H NMR (400 MHz, DMSO-d_6) spectrum of (2,5-dimethoxyphenyl)(5-hydroxy-1-benzofuran-3-yl)methanone (3h).

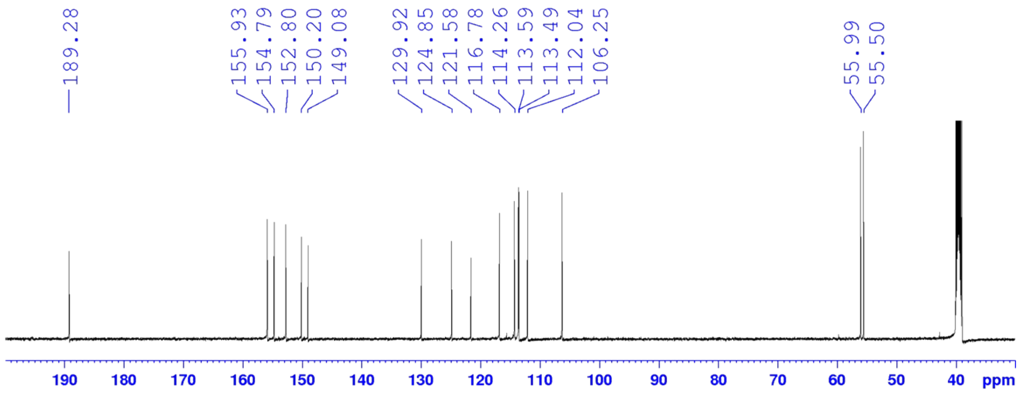

Plate 7b: The ^{13}C NMR (125 MHz, DMSO-d_6) spectrum of (2,5-dimethoxyphenyl)(5-hydroxy-1-benzofuran-3-yl)methanone (3h).

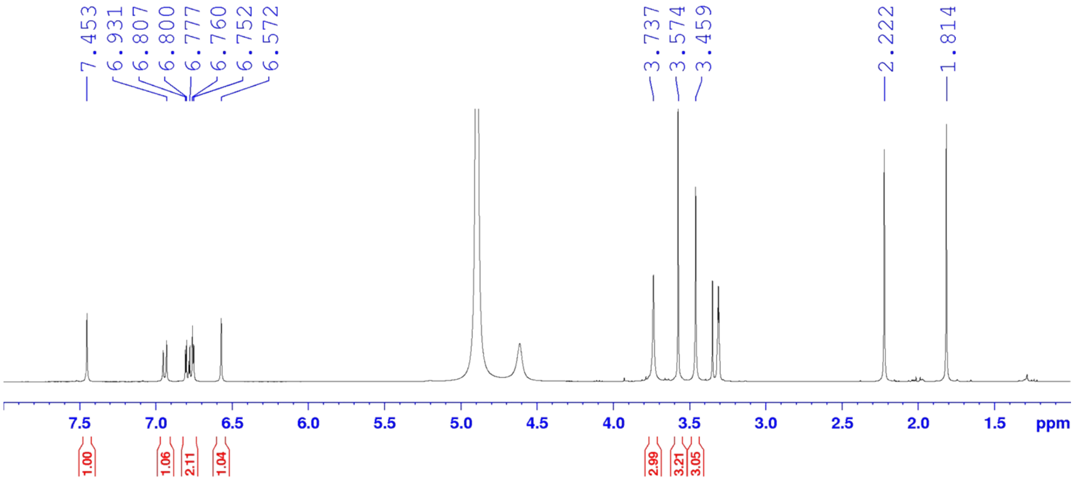

Plate 8a: The ^{1}H NMR (400 MHz, Methanol-d_4) spectrum of 2-[3-(2,5-dimethoxyphenyl)-1*H*-pyrazol-4-yl]-4-methoxy-3,6-dimethylphenol (5a).

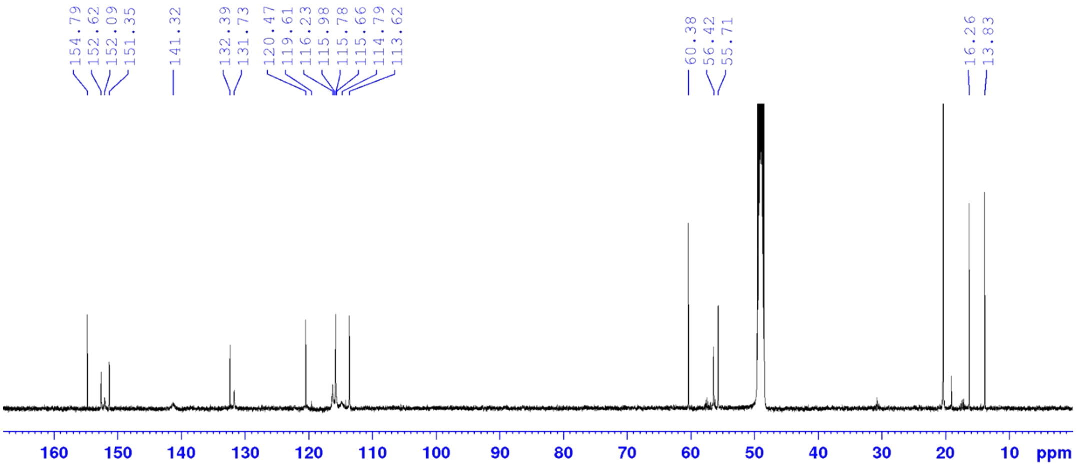

Plate 8b: The ^{13}C NMR (125 MHz, Methanol-d_4) spectrum of 2-[3-(2,5-dimethoxyphenyl)-1*H*-pyrazol-4-yl]-4-methoxy-3,6-dimethylphenol (5a).

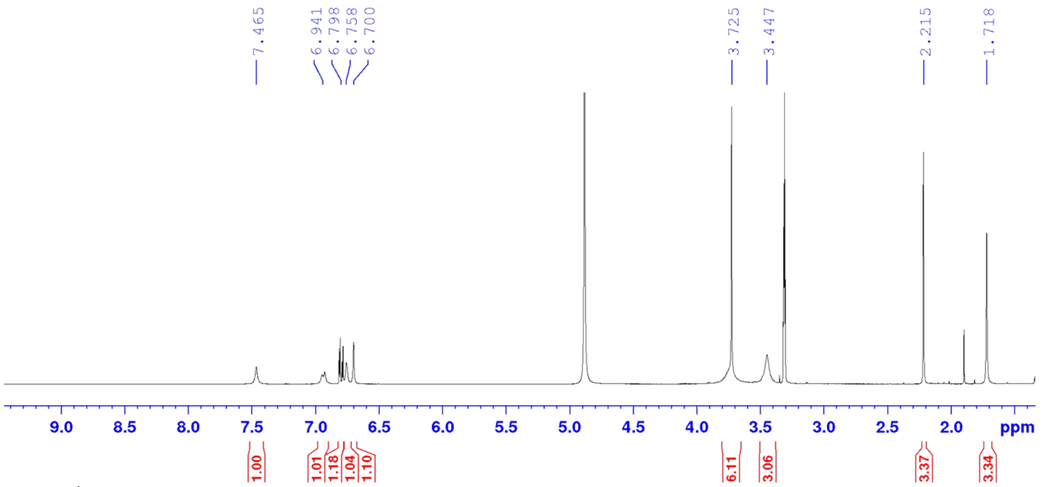

Plate 9a: The ^{1}H NMR (400 MHz, Methanol-*d*$_4$) spectrum for 2-[3-(2,5-dimethoxyphenyl)-1H-pyrazol-4-yl]-4-methoxy-3,5-dimethylphenol (5b).

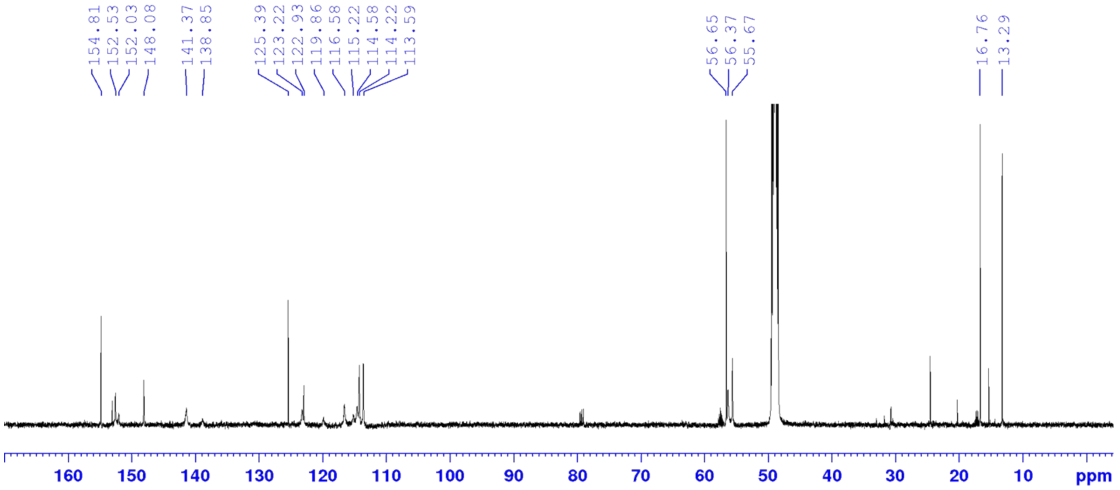

Plate 9b: The ^{13}C NMR (125 MHz, Methanol-*d*$_4$) spectrum of 2-[3-(2,5-dimethoxyphenyl)-1H-pyrazol-4-yl]-4-methoxy-3,5-dimethylphenol (5b).

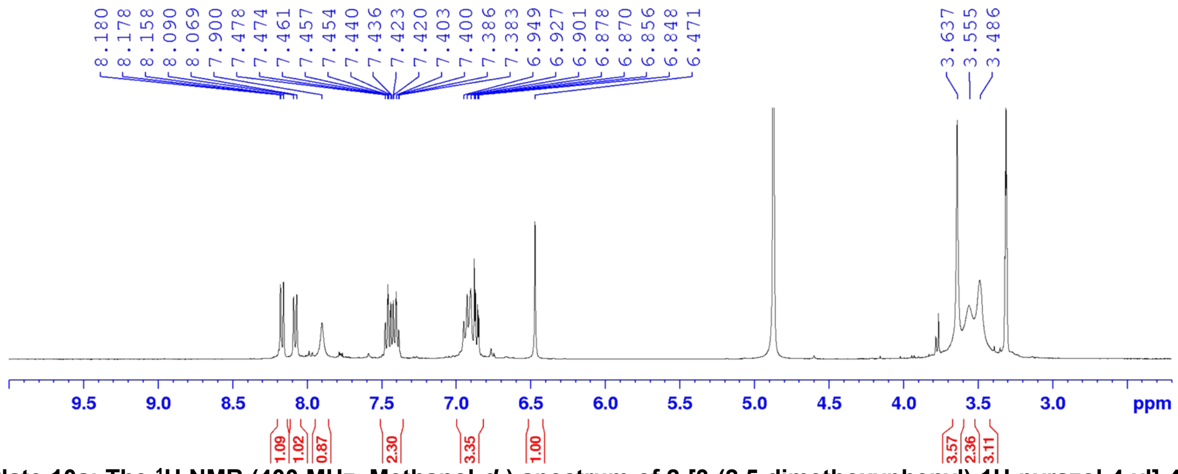

Plate 10a: The ^{1}H NMR (400 MHz, Methanol-*d*$_4$) spectrum of 2-[3-(2,5-dimethoxyphenyl)-1H-pyrazol-4-yl]-4-methoxynaphthalen-1-ol (5c).

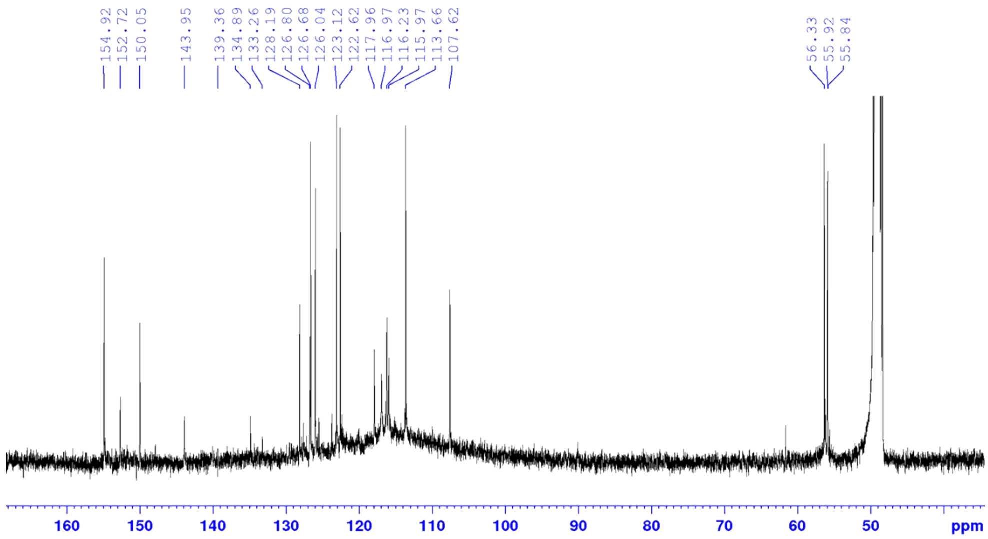

Plate 10b: The ^{13}C NMR (100 MHz, Methanol-d_4) spectrum for 2-[3-(2,5-dimethoxyphenyl)-1H-pyrazol-4-yl]-4-methoxynaphthalen-1-ol (5c).

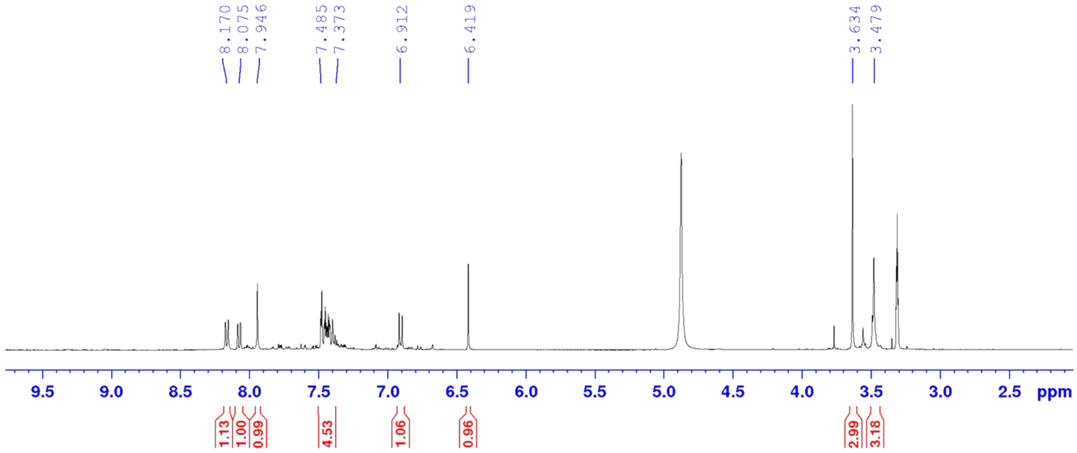

Plate 11a: The ^{1}H NMR (400 MHz, Methanol-d_4) spectrum of 2-[3-(5-bromo-2-methoxyphenyl)-1*H*-pyrazol-4-yl]-4-methoxynaphthalen-1-ol (5d).

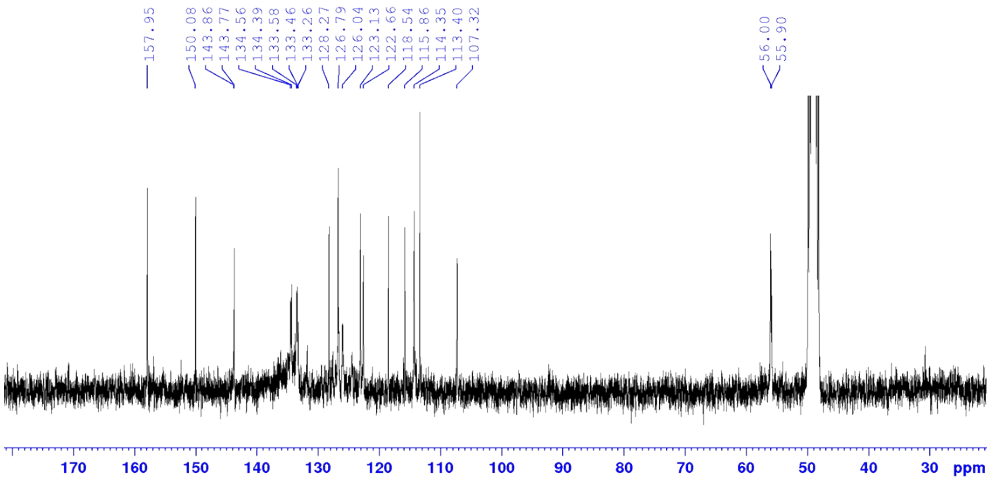

Plate 11b: The ^{13}C NMR (75 MHz, Methanol-d_4) spectrum of 2-[3-(5-bromo-2-methoxyphenyl)-1*H*-pyrazol-4-yl]-4-methoxynaphthalen-1-ol (5d).

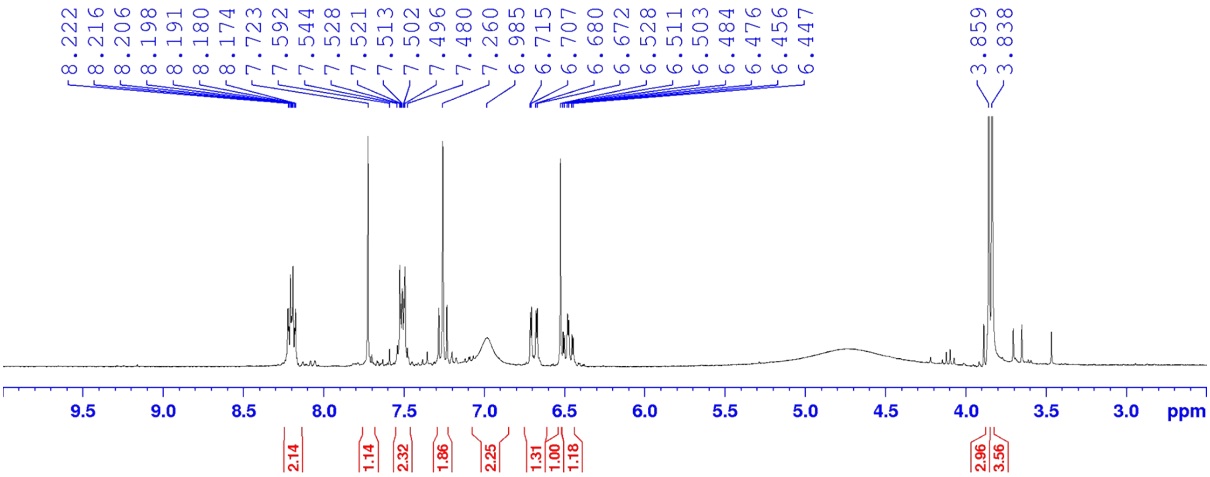

Plate 12a: The ^{1}H NMR (300 MHz, Chloroform-*d*$_1$) spectrum of 2-[3-(4-fluoro-2-methoxyphenyl)-1*H*-pyrazol-4-yl]-4-methoxynaphthalen-1-ol (5e).

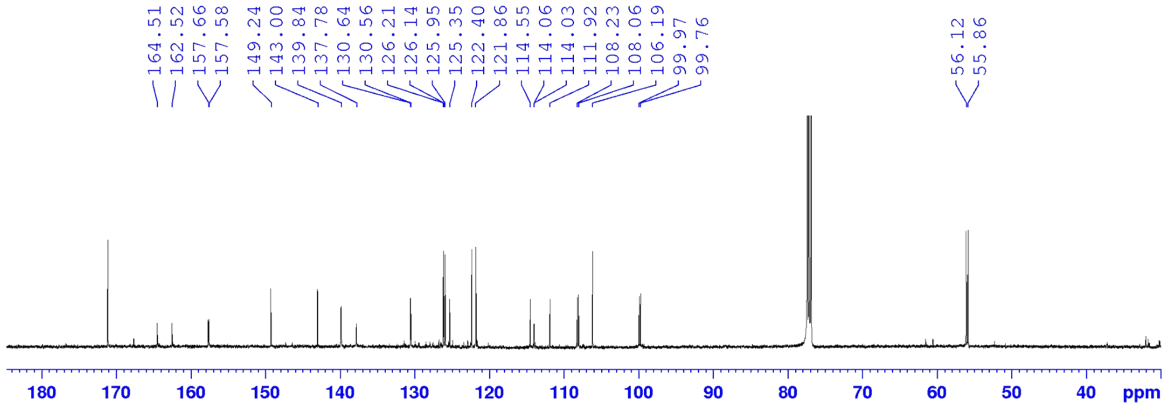

Plate 12b: The ^{13}C NMR (125 MHz, Chloroform-*d*$_1$) spectrum of 2-[3-(4-fluoro-2-methoxyphenyl)-1*H*-pyrazol-4-yl]-4-methoxynaphthalen-1-ol (5e).

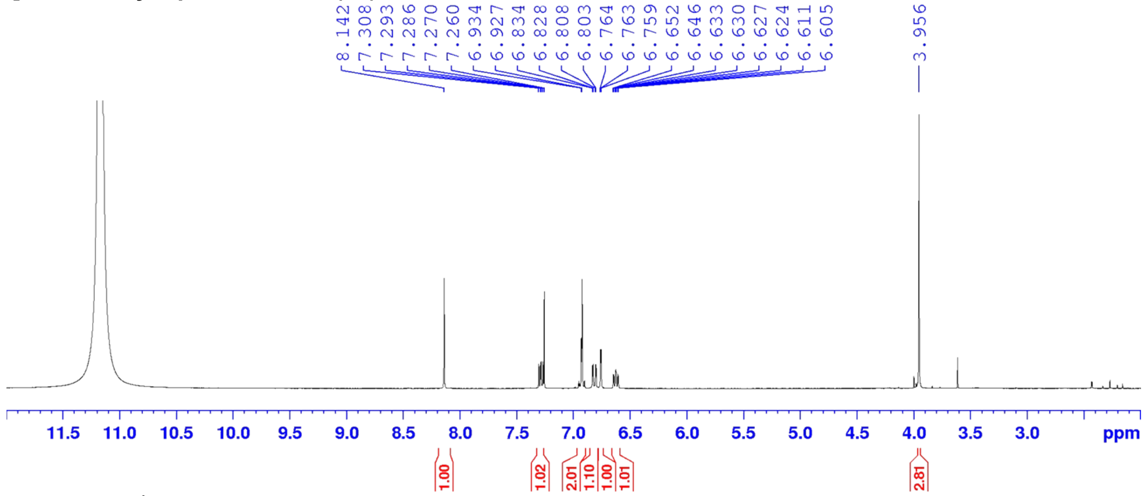

Plate 13a: The ^{1}H NMR (400 MHz, Chloroform-*d*$_1$) spectrum for 2-[3-(4-fluoro-2-methoxyphenyl)-1H-pyrazol-4-yl]benzene-1,4-diol (5f).

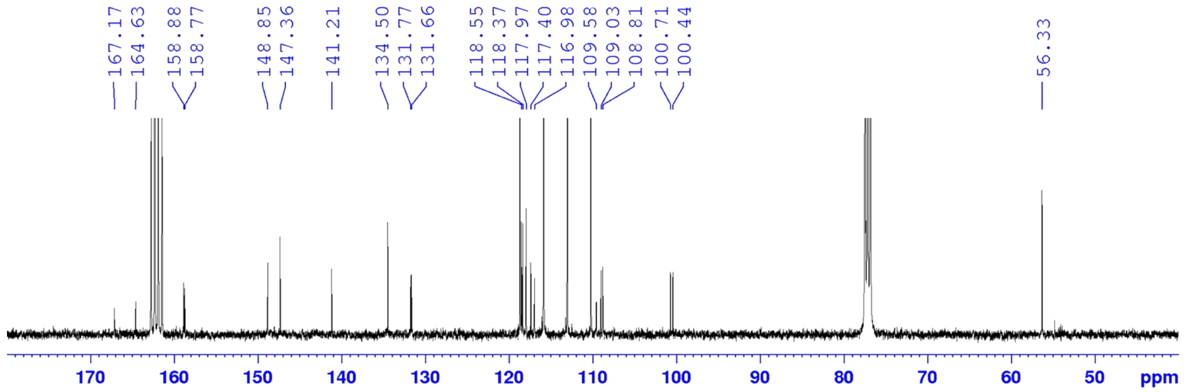

Plate 13b: The ^{13}C NMR (100 MHz, Chloroform-d_1) spectrum for 2-[3-(4-fluoro-2-methoxyphenyl)-1H-pyrazol-4-yl]benzene-1,4-diol (5f).

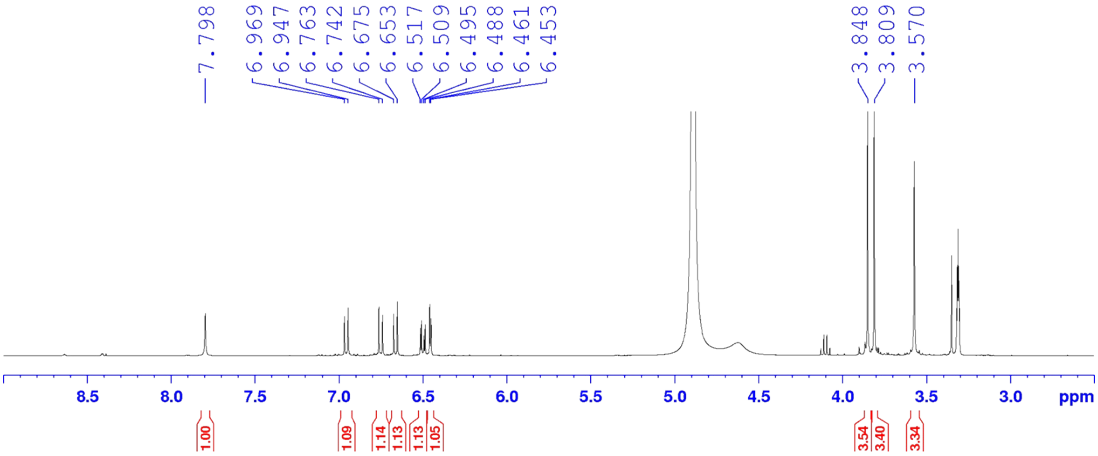

Plate 14a: The ^{1}H NMR (400 MHz, Methanol-d_4) spectrum for 2-[3-(2,3,4-trimethoxyphenyl)-1H-pyrazol-4-yl]benzene-1,4-diol (5g).

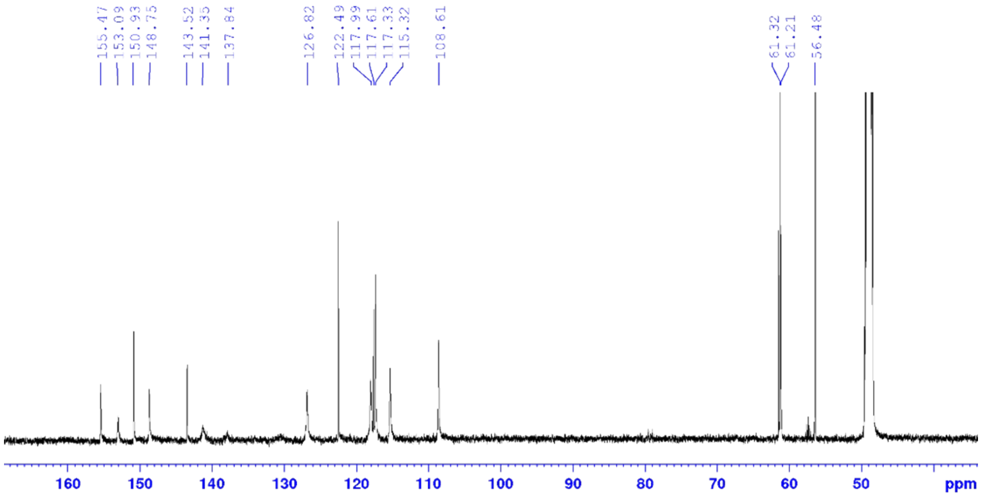

Plate 14b: The ^{13}C NMR (100 MHz, Methanol-d_4) spectrum for 2-[3-(2,3,4-trimethoxyphenyl)-1H-pyrazol-4-yl]benzene-1,4-diol (5g).

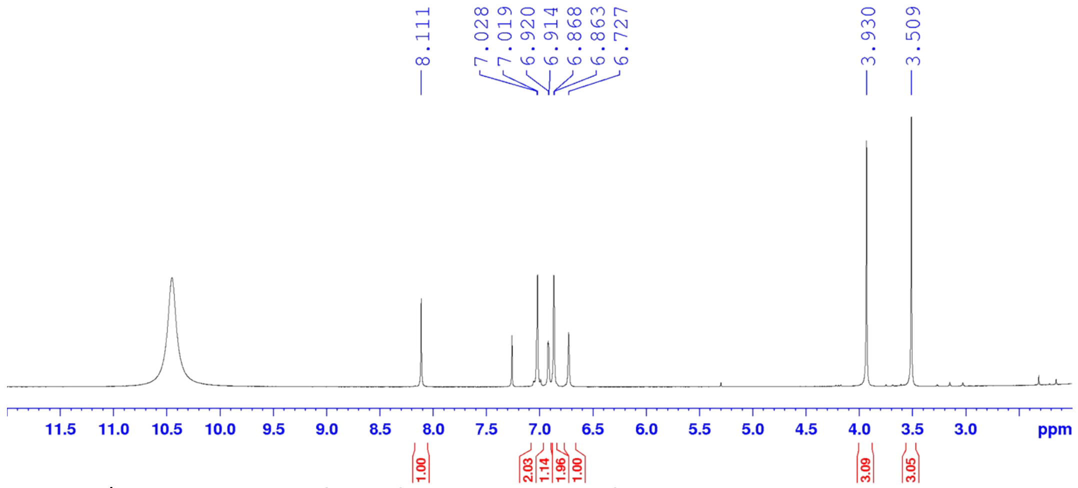

Plate 15a: The ^{1}H NMR (300 MHz, Chloroform-d_1) spectrum for 2-[3-(2,5-dimethoxyphenyl)-1H-pyrazol-4-yl]benzene-1,4-diol (5h).

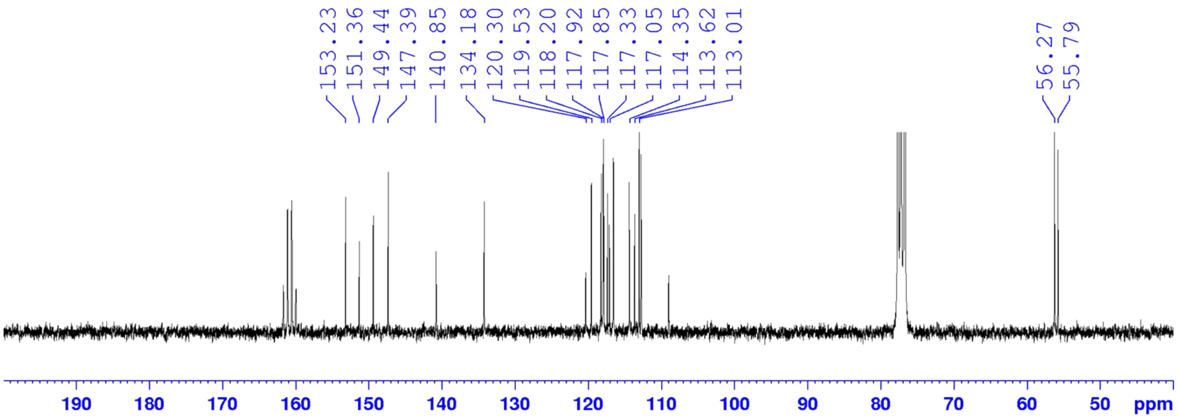

Plate 15b: The ^{13}C NMR (75 MHz, Chloroform-d_1) spectrum for 2-[3-(2,5-dimethoxyphenyl)-1H-pyrazol-4-yl]benzene-1,4-diol (5h).

1.2.2 Chromatograms

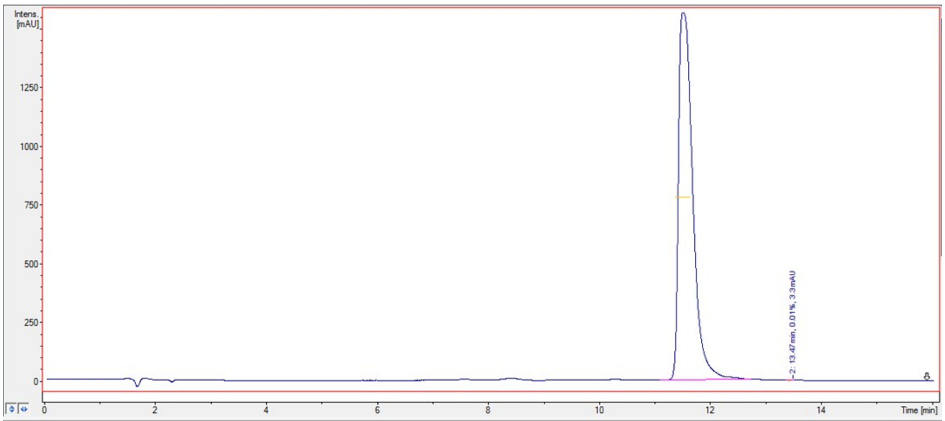

No	Time [min]	Intensity [...	Integral [%]	Integral [mAU]	Annotation	Baselin...	Baseline ...	Valley Sta...	Valley En...	Half Height Line Width [µl]	Half Height Line Wi...
1	11.46	1562.18	99.99	138875.71		11.08	12.73	11.24	0.04	133.59	0.27
2	13.43	3.25	0.01	10.28		13.35	13.50	0.04	0.04	33.66	0.07

Plate 1c: UV chromatogram of (2,5-dimethoxyphenyl)(5-methoxy-4,7-dimethyl-1-benzofuran-3-yl)methanone (4a) measured at 280 nm.

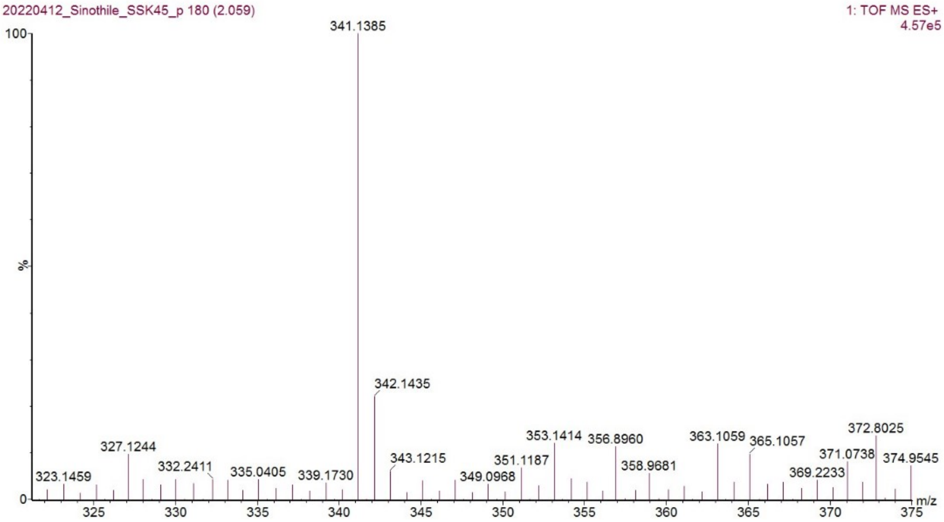

Plate 1d: HR-MS spectrum of (2,5-dimethoxyphenyl)(5-methoxy-4,7-dimethyl-1-benzofuran-3-yl)methanone (4a).

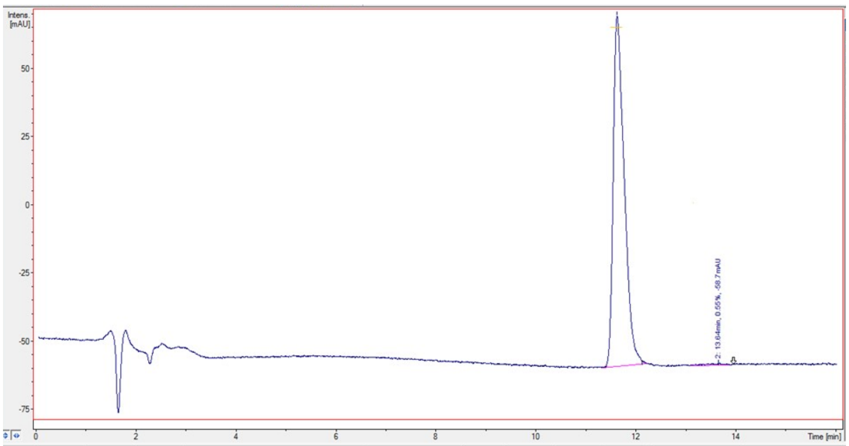

No	Time [min]	Intensity [...	Integral [%]	Integral [mAU]	Annotation	Baselin...	Baseline ...	Valley Sta...	Valley En...	Half Height Line Width [µl]	Half Height Line Width [...
☑ 1	11.57	68.64	99.45	9777.51		11.32	12.22	11.39	12.12	119.55	0.24
☑ 2	13.59	-58.69	0.55	54.46		13.08	13.87	0.05	13.64	8.17	0.02

Plate 2c: UV chromatogram of (2,5-dimethoxyphenyl)(5-methoxy-4,6-dimethyl-1-benzofuran-3-yl)methanone (4b) measured at 280 nm.

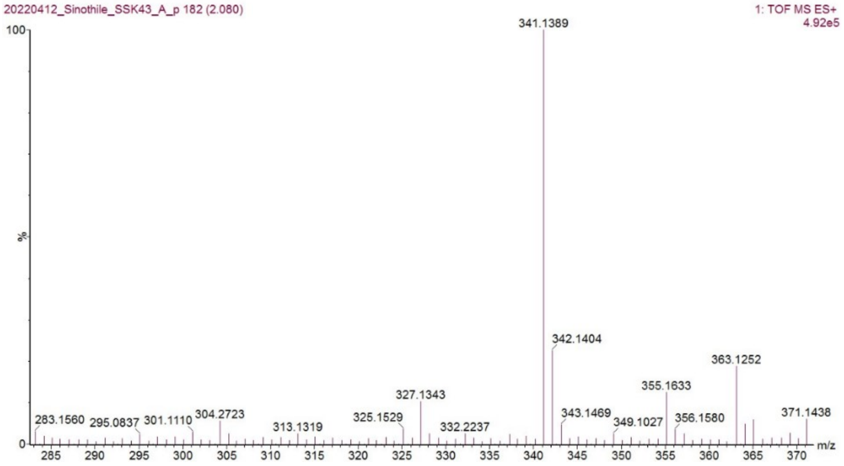

Plate 2d: HR-MS spectrum of (2,5-dimethoxyphenyl)(5-methoxy-4,6-dimethyl-1-benzofuran-3-yl)methanone (4b).

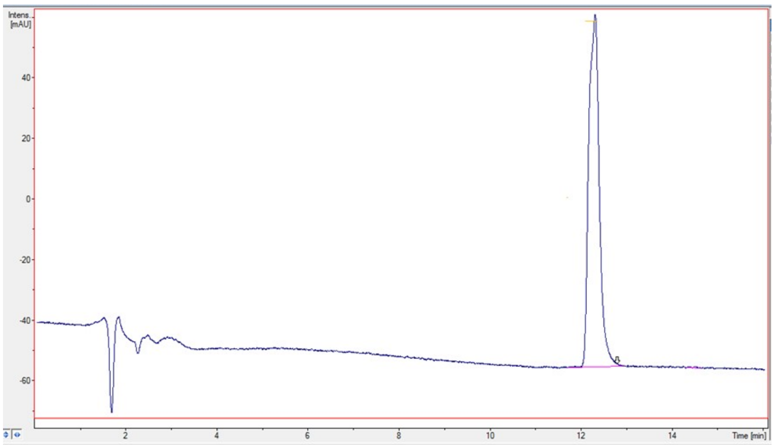

No	Time [min]	Intensity [...	Integral [%]	Integral [mAU]	Annotation	Baselin...	Baseline ...	Valley Sta...	Valley En...	Half Height Line Width [µl
☐ 1	11.95	-55.52	0.19	17.85		11.72	12.94	0.05	12.01	16.51
☐ 2	12.25	60.32	99.58	9317.16		11.72	12.94	12.01	12.92	127.44
☐ 3	14.56	-55.87	0.23	21.79		14.31	14.62	0.05	0.05	7191.21

Plate 3c: UV chromatogram of (2,5-dimethoxyphenyl)(5-methoxynaphtho[1,2-*b*]furan-3-yl)methanone (4c).

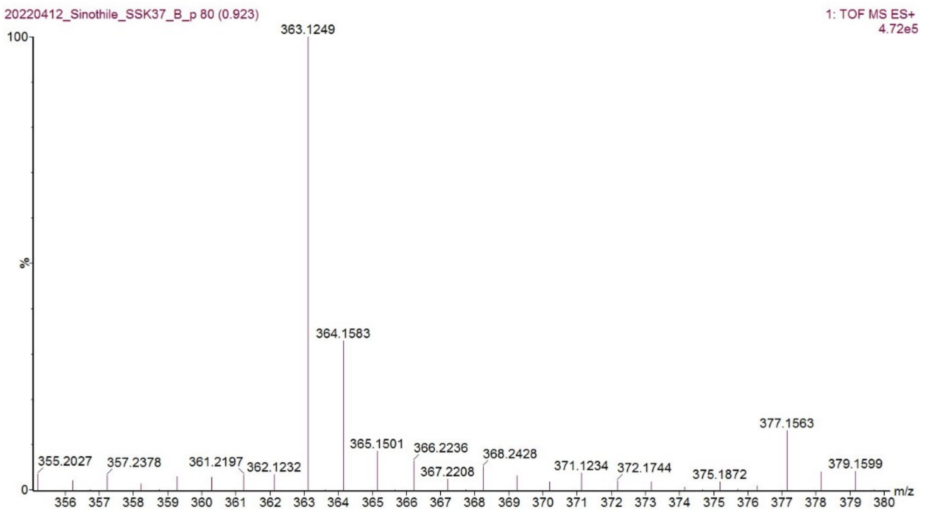

Plate 3d: HR-MS spectrum of (2,5-dimethoxyphenyl)(5-methoxynaphtho[1,2-*b*]furan-3-yl)methanone (4c).

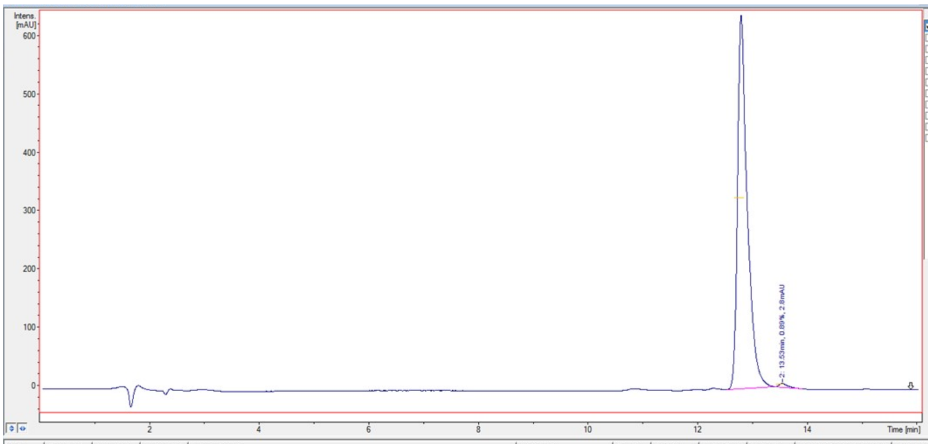

No	Time [min]	Intensity [...	Integral [%]	Integral [mAU]	Annotation	Baselin...	Baseline ...	Valley Sta...	Valley En...	Half Height Line Width [µl]	Half Heig
1	12.74	630.69	99.11	39448.29		12.53	13.38	0.05	0.05	88.42	0.18
2	13.49	2.78	0.89	355.41		13.40	13.91	0.05	0.05	72.93	0.15

Plate 4c: UV chromatogram of (5-bromo-2-methoxyphenyl)(5-methoxynaphtho[1,2-*b*]furan-3-yl)methanone (4d) measured at 280 nm.

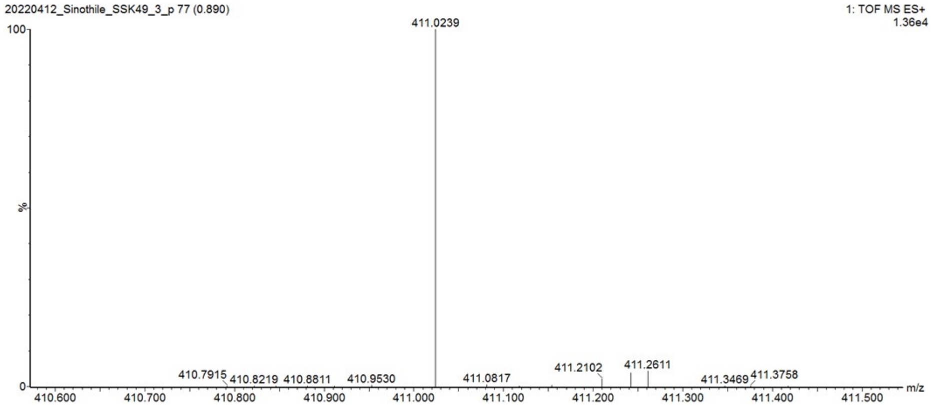

Plate 4d: HR-MS spectrum of (5-bromo-2-methoxyphenyl)(5-methoxynaphtho[1,2-*b*]furan-3-yl)methanone (4d).

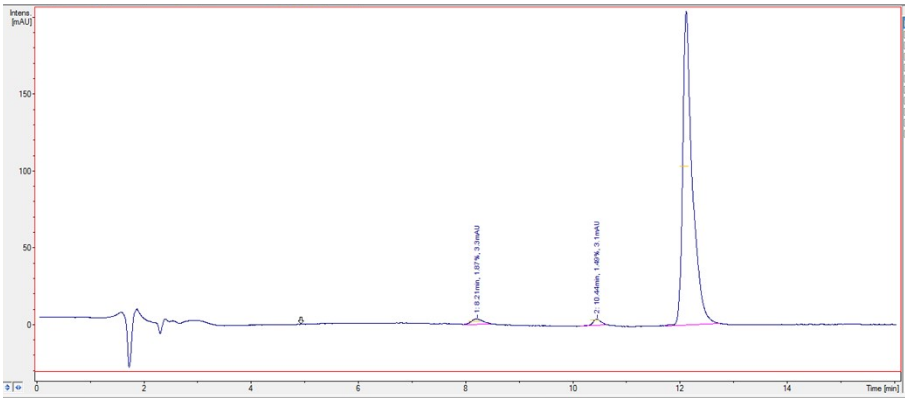

No	Time [min]	Intensity [...	Integral [%]	Integral [mAU]	Annotation	Baselin...	Baseline ...	Valley Sta...	Valley En...	Half Height Line Width [µl]	Half Height Line Width [...	Half Hei
1	8.17	3.32	1.87	244.54		7.96	8.46	0.05	0.05	112.73	0.23	1.67
2	10.40	3.11	1.49	194.27		10.14	10.62	10.31	0.05	81.81	0.16	1.95
3	12.06	202.77	96.65	12642.82		11.72	12.74	11.88	0.05	83.33	0.17	101.67

Plate 5c: UV chromatogram of (4-fluoro-2-methoxyphenyl)(5-methoxynaphtho[1,2-b]furan-3-yl)methanone (4e) measured at 280 nm.

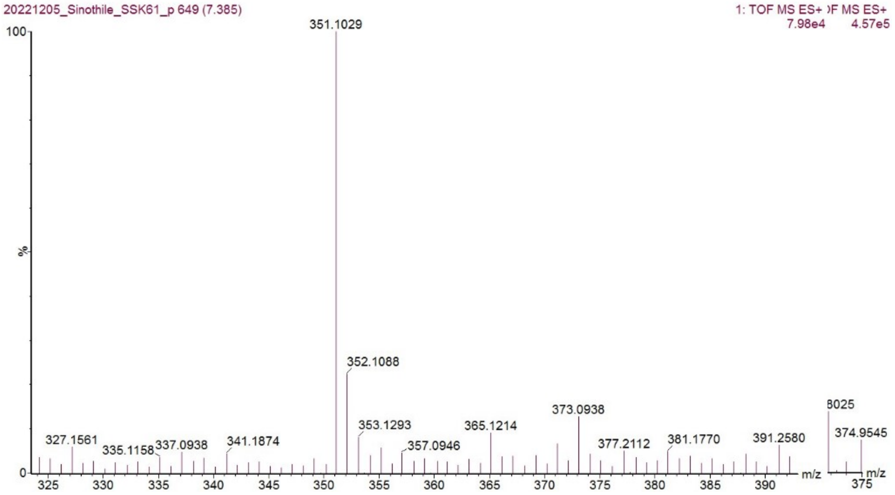

Plate 5d: HR-MS spectrum of (4-fluoro-2-methoxyphenyl)(5-methoxynaphtho[1,2-b]furan-3-yl)methanone (4e).

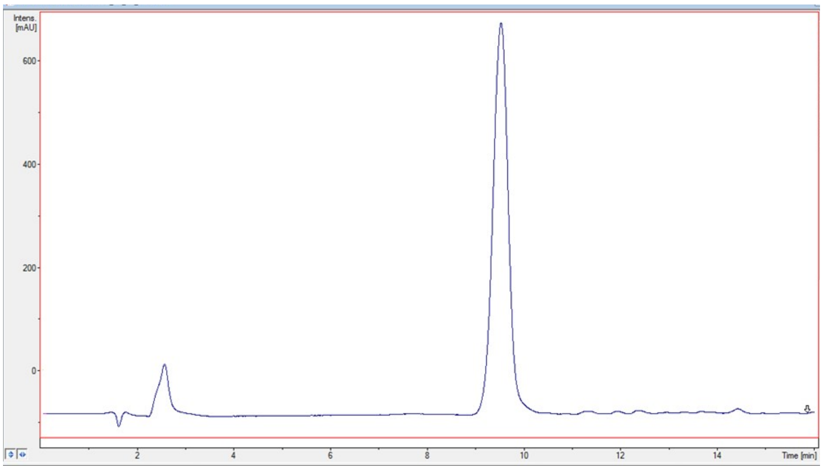

No	Time [min]	Intensity [...	Integral [%]	Integral [mAU]	Annotation	Baselin...	Baseline ...	Valley Sta...	Valley En.
1	0.04	-84.13	100.00	1.12		0.07	0.11	0.05	0.05

Plate 6c: UV chromatogram of (5-hydroxy-1-benzofuran-3-yl)(2,3,4-trimethoxyphenyl)methanone (3g) measured at 280 nm.

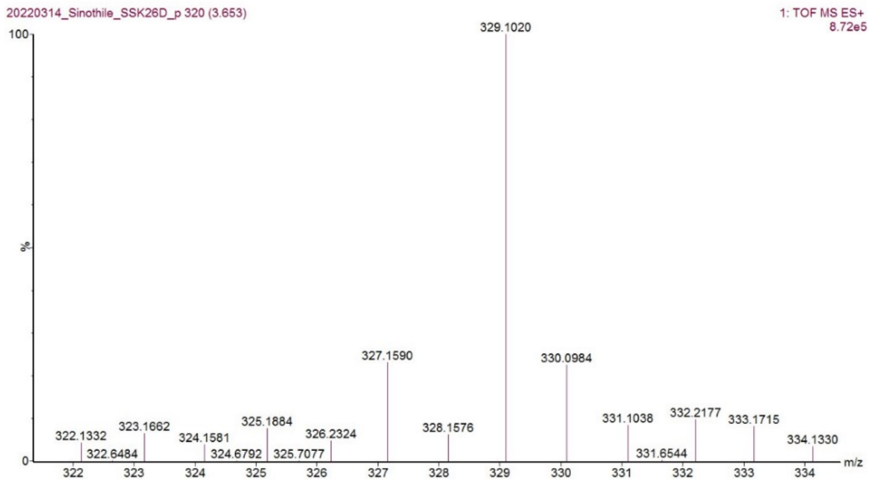

Plate 6d: HR-MS spectrum of (5-hydroxy-1-benzofuran-3-yl)(2,3,4-trimethoxyphenyl)methanone (3g).

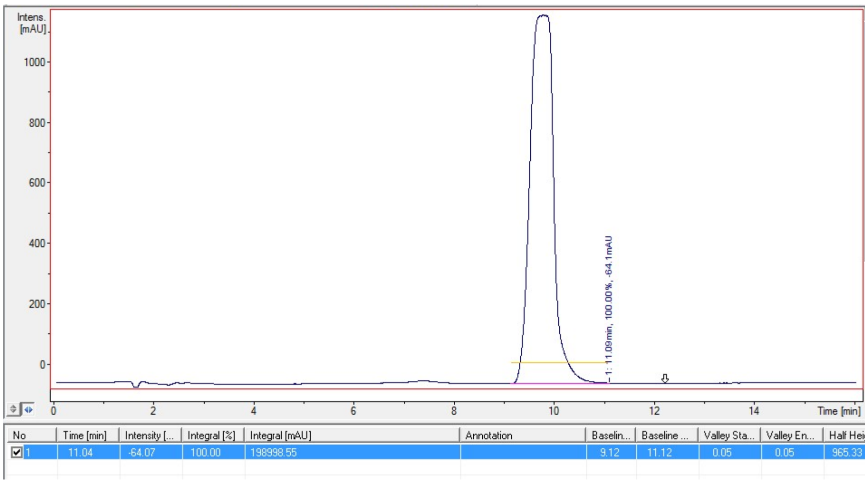

Plate 7c: UV chromatogram of (2,5-dimethoxyphenyl)(5-hydroxy-1-benzofuran-3-yl)methanone (3h) measured at 280 nm.

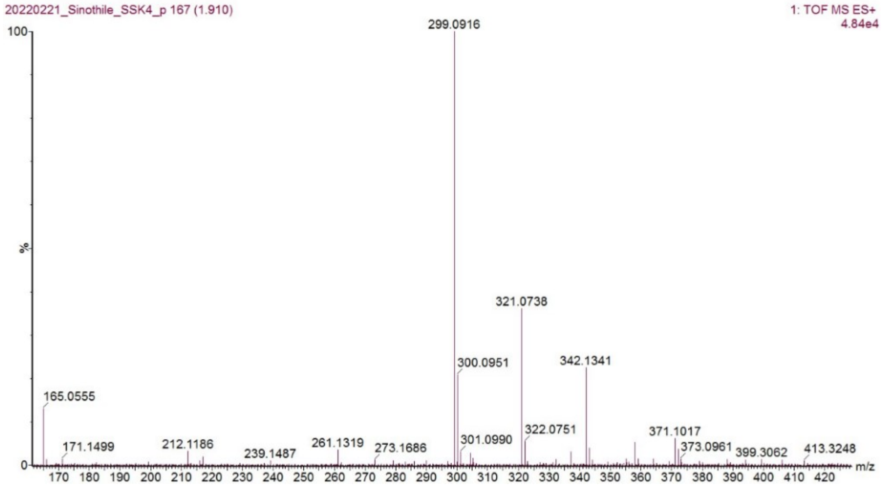

Plate 7d: HR-MS spectrum of (2,5-dimethoxyphenyl)(5-hydroxy-1-benzofuran-3-yl)methanone (3h).

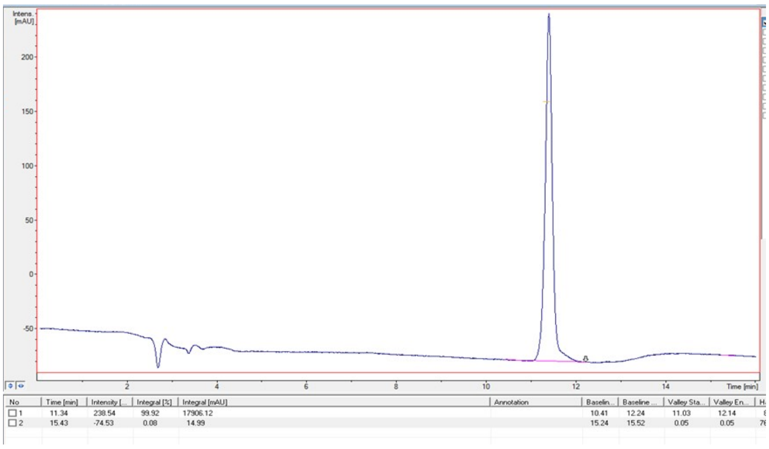

Plate 8c: UV chromatogram of 2-[3-(2,5-dimethoxyphenyl)-1*H*-pyrazol-4-yl]-4-methoxy-3,6-dimethylphenol (5a) measured at 280 nm.

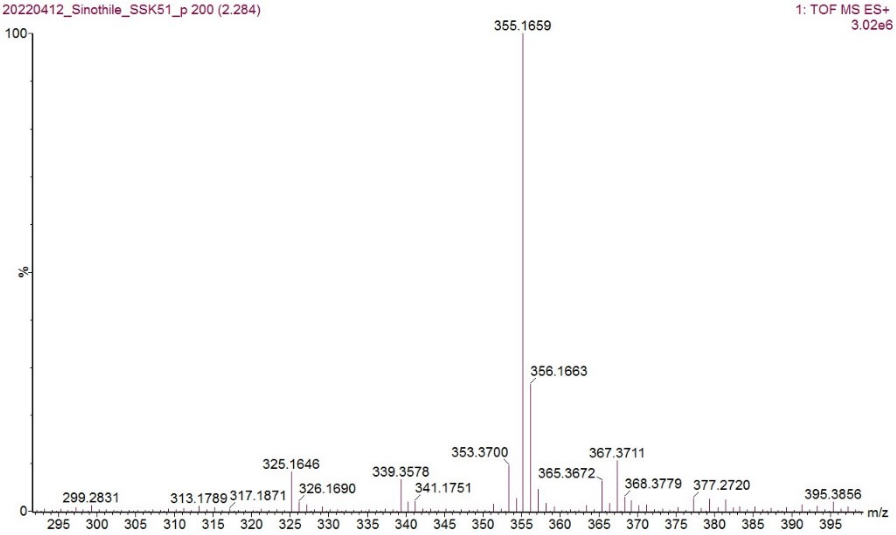

Plate 8d: HR-MS spectrum of 2-[3-(2,5-dimethoxyphenyl)-1*H*-pyrazol-4-yl]-4-methoxy-3,6-dimethylphenol (5a).

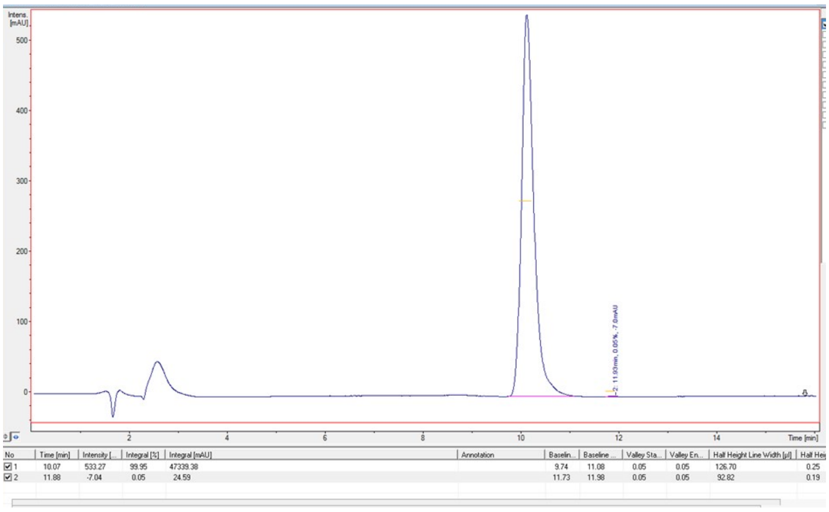

Plate 9c: UV chromatogram of 2-[3-(2,5-dimethoxyphenyl)-1H-pyrazol-4-yl]-4-methoxy-3,5-dimethylphenol (5b) measured at 280 nm.

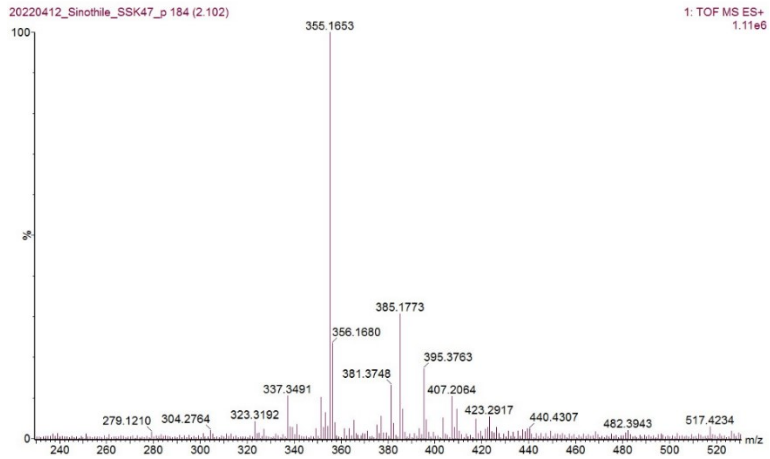

Plate 9d: HR-MS spectrum for 2-[3-(2,5-dimethoxyphenyl)-1H-pyrazol-4-yl]-4-methoxy-3,5 dimethylphenol (5b).

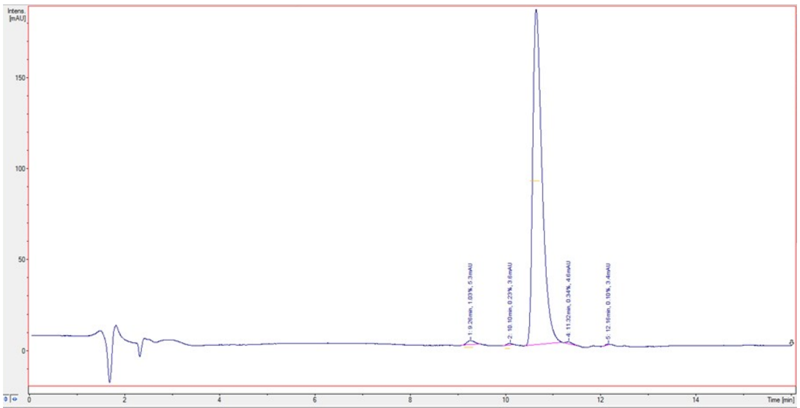

No	Time [min]	Intensity [...	Integral [%]	Integral [mAU]	Annotation	Baselin...	Baseline ...	Valley Sta...	Valley En...	Half Height Line Width [µl]	Half Height Line Width [...	Half Height [mAU
1	9.21	5.28	1.03	131.35		9.06	9.46	0.05	0.05	97.19	0.19	1.16
2	10.05	3.64	0.23	28.93		9.95	10.16	0.05	0.05	57.60	0.12	0.36
3	10.59	186.80	98.30	12502.32		10.41	11.17	0.05	0.05	102.17	0.20	91.91
4	11.27	4.57	0.34	43.19		11.23	11.50	0.05	0.05	5661.79	11.32	0.52
5	12.11	3.38	0.10	12.44		12.04	12.19	0.05	0.05	0.00	0.00	0.19

Plate 10c: UV chromatogram of 2-[3-(2,5-dimethoxyphenyl)-1H-pyrazol-4-yl]-4-methoxynaphthalen-1-ol (5c) measured at 260 nm.

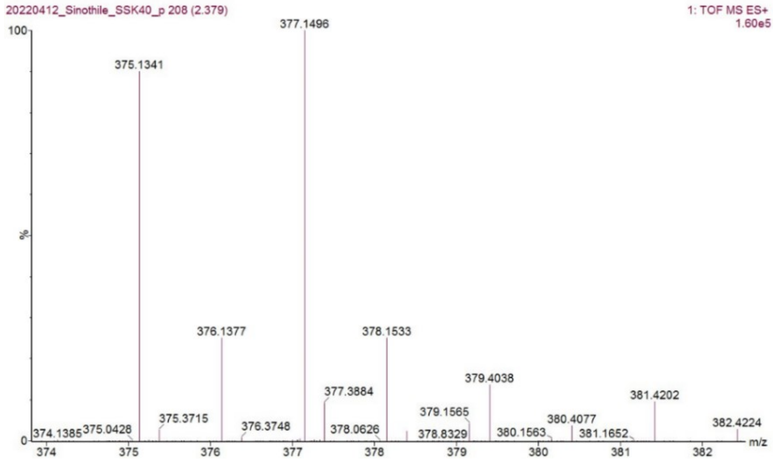

Plate 10d: HR-MS spectrum for 2-[3-(2,5-dimethoxyphenyl)-1H-pyrazol-4-yl]-4-methoxynaphthalen-1-ol (5c).

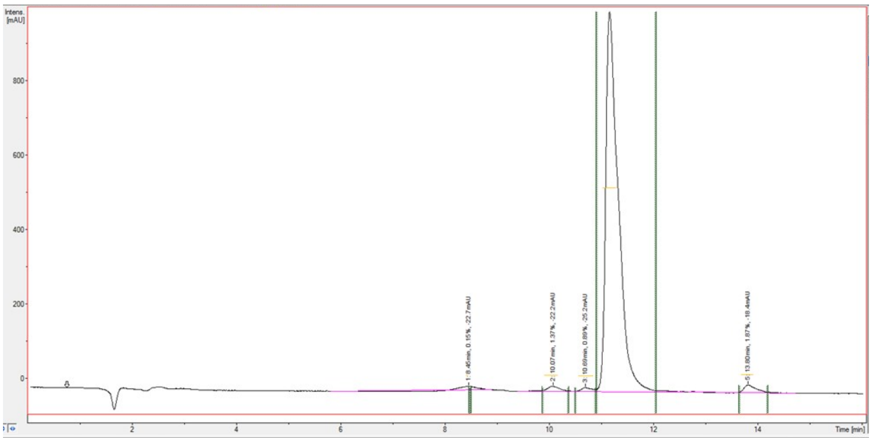

No	Time [min]	Intensity [...	Integral [%]	Integral [mAU]	Annotation	Baselin...	Baseline ...	Valley Sta...	Valley En...	Half Height Line Width [µl]	Half Height Line Width [...
☑ 1	8.41	-22.68	0.15	136.91		5.81	8.90	8.45	8.50	25.00	0.05
☑ 2	10.02	-22.16	1.37	1217.38		9.39	12.59	9.86	10.37	130.78	0.26
☑ 3	10.64	-25.17	0.89	787.33		9.39	12.59	10.48	10.88	138.27	0.28
☑ 4	11.11	981.50	95.71	84847.96		9.39	12.59	10.88	12.05	127.12	0.25
☑ 5	13.76	-18.45	1.87	1658.18		12.66	14.72	13.62	14.19	121.39	0.24

Plate 11c: UV chromatogram of 2-[3-(5-bromo-2-methoxyphenyl)-1*H*-pyrazol-4-yl]-4-methoxynaphthalen-1-ol (5d) measured at 260 nm.

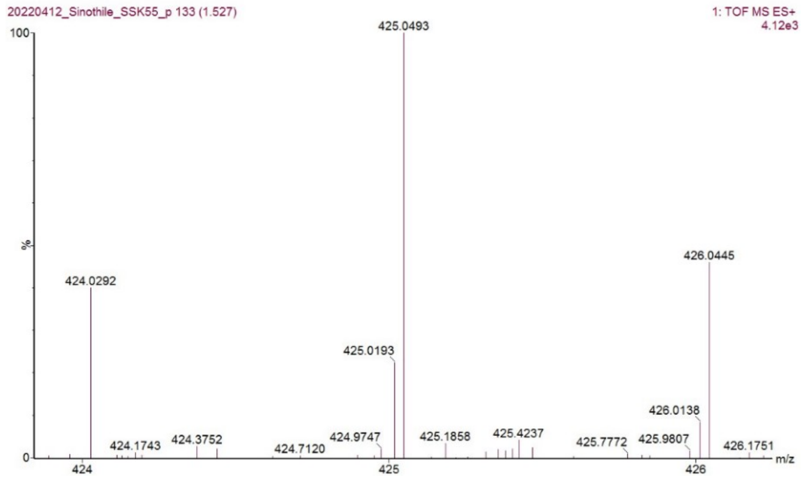

Plate 11d: HR-MS spectrum of 2-[3-(5-bromo-2-methoxyphenyl)-1*H*-pyrazol-4-yl]-4-methoxynaphthalen-1-ol (5d).

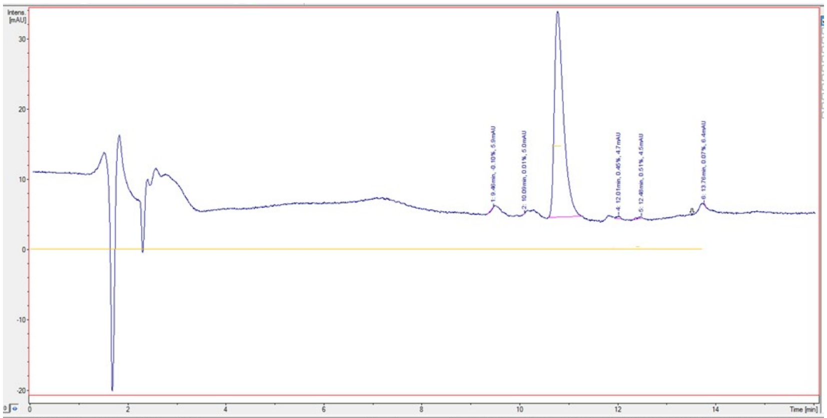

No	Time [min]	Intensity [...	Integral [%]	Integral [mAU]	Annotation	Baselin...	Baseline ...	Valley Sta...	Valley En...	Half Height Line Width [µl]	Half Height Line Width [...	Half Height [mAU]
☑ 1	9.41	5.91	-0.10	-1.82		9.33	9.46	0.05	0.05	0.00	0.00	-0.01
☑ 2	10.05	5.00	0.01	0.23		10.07	10.10	0.05	0.05	0.00	0.00	0.00
☑ 3	10.72	33.66	99.05	1886.01		10.58	11.25	0.05	0.05	95.82	0.19	14.58
☑ 4	11.97	4.69	0.45	8.57		11.94	12.07	0.05	0.05	5.94	0.01	0.20
☑ 5	12.43	4.54	0.51	9.75		12.32	12.50	0.05	0.05	40.33	0.08	0.10
☑ 6	13.71	6.38	0.07	1.41		13.75	13.80	0.05	0.05	6861.90	13.72	0.06

Plate 12c: UV chromatogram of 2-[3-(4-fluoro-2-methoxyphenyl)-1H-pyrazol-4-yl]-4-methoxynaphthalen-1-ol (5e).

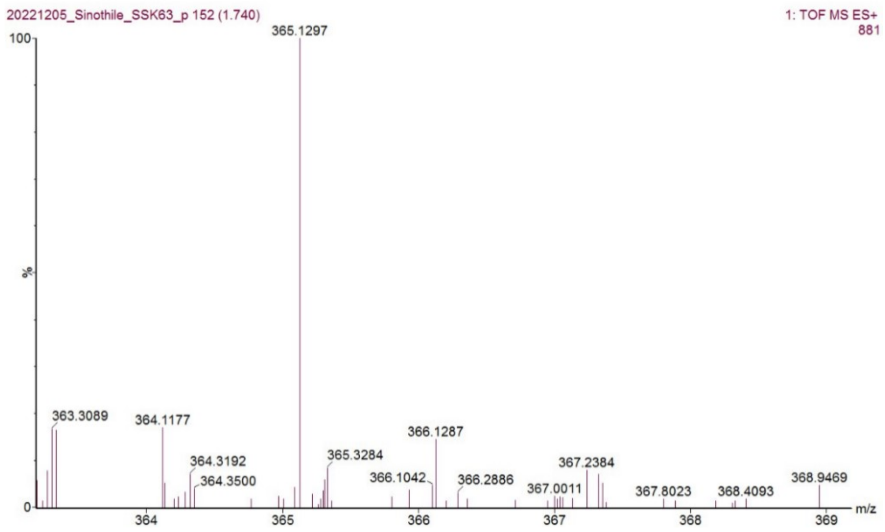

Plate 12d: HR-MS spectrum of 2-[3-(4-fluoro-2-methoxyphenyl)-1*H*-pyrazol-4-yl]-4-methoxynaphthalen-1-ol (5e).

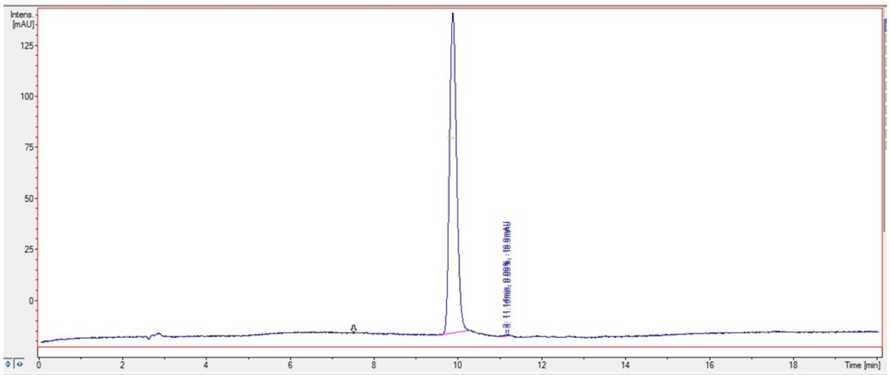

No	Time [min]	Intensity [...	Integral [%]	Integral [mAU]	Annotation	Baselin...	Baseline ...	Valley Sta...	Valley En...	Half Height
1	9.82	139.76	99.82	9126.89		9.51	10.25	9.61	0.05	91.50
2	11.09	-16.91	0.09	8.11		11.02	11.24	11.12	11.17	23.33
3	11.14	-16.95	0.09	8.33		11.02	11.24	11.17	0.05	18.50

Plate 13c: UV chromatogram of 2-[3-(4-fluoro-2-methoxyphenyl)-1H-pyrazol-4-yl]benzene-1,4-diol (5f) measured at 280 nm.

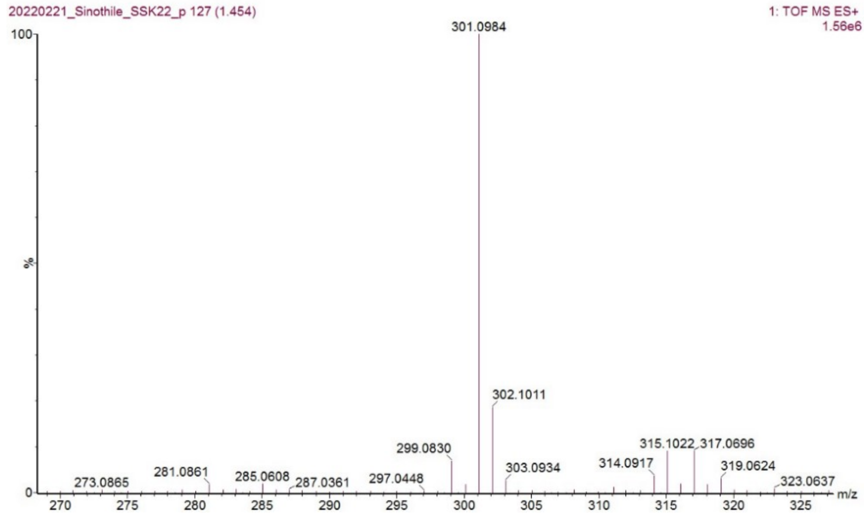

Plate 13d: HR-MS spectrum of 2-[3-(4-fluoro-2-methoxyphenyl)-1H-pyrazol-4-yl]benzene-1,4-diol (5f).

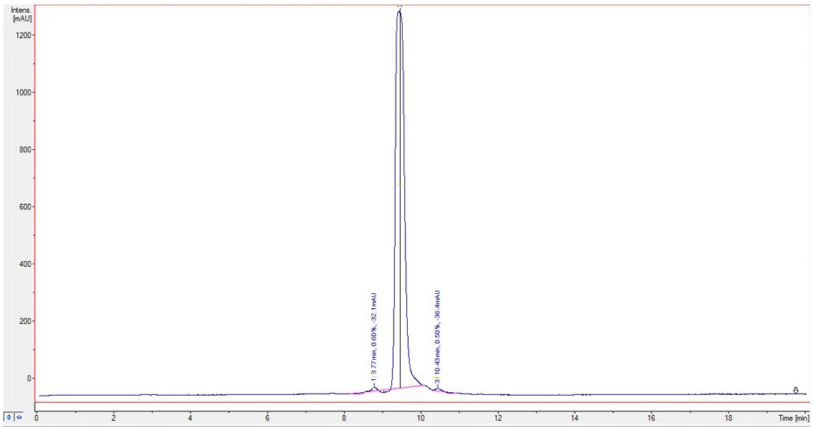

No	Time [min]	Intensity [...	Integral [%]	Integral [mAU]	Annotation	Baselin...	Baseline ...	Valley Sta...	Valley En...	Half Height Line Wi...
1	8.73	-32.10	0.60	336.38		8.15	10.05	8.63	9.02	68.77
2	9.40	1274.21	98.90	55851.42		8.15	10.05	9.44	0.05	63.53
3	10.38	-36.41	0.50	284.60		10.31	10.92	0.05	10.77	75.86

Plate 14c: UV chromatogram of 2-[3-(2,3,4-trimethoxyphenyl)-1H-pyrazol-4-yl]benzene-1,4-diol (5g) at 280 nm.

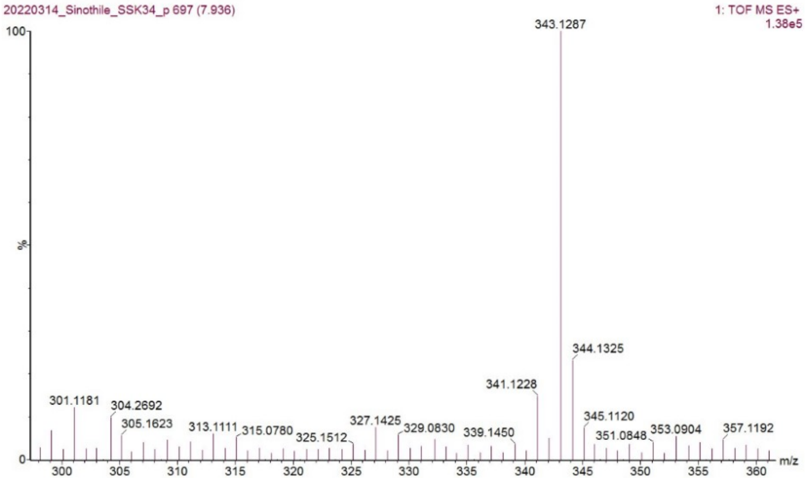

Plate 14d: HR-MS spectrum of 2-[3-(2,3,4-trimethoxyphenyl)-1H-pyrazol-4-yl]benzene-1,4-diol (5g).

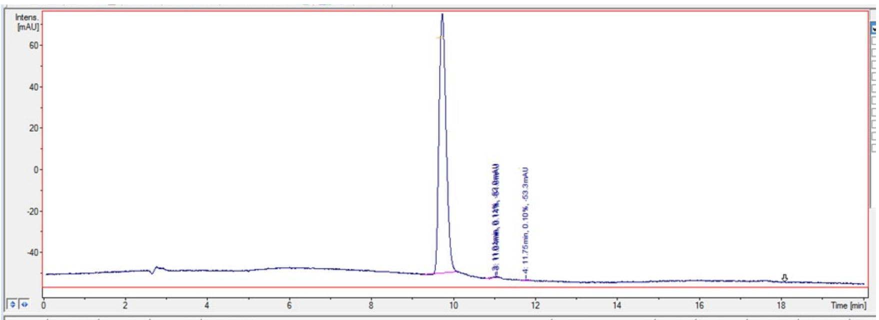

No	Time [min]	Intensity [...	Integral [%]	Integral [mAU]	Annotation	Baselin...	Baseline ...	Valley Sta...	Valley En...	Half H...
1	9.67	74.15	99.65	7473.60		9.24	10.09	9.49	0.05	95.3
2	10.97	-52.00	0.11	8.11		10.78	11.10	0.05	11.02	6.27
3	10.99	-51.58	0.14	10.37		10.78	11.10	11.02	0.05	30.3
4	11.70	-53.32	0.10	7.40		11.63	11.88	0.05	11.78	7.70

Plate 15c: UV chromatogram for 2-[3-(2,5-dimethoxyphenyl)-1H-pyrazol-4-yl]benzene-1,4-diol (5h) measured at 280 nm.

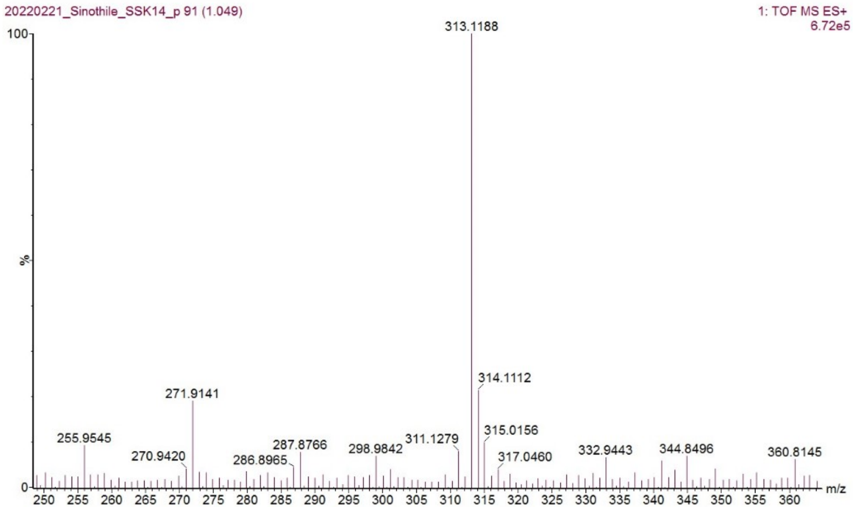

Plate 15d: HR-MS spectrum for 2-[3-(2,5-dimethoxyphenyl)-1H-pyrazol-4-yl]benzene-1,4-diol (5h) measured at 280 nm.
